# Cerebral oxygenation during locomotion is modulated by respiration

**DOI:** 10.1038/s41467-019-13523-5

**Published:** 2019-12-04

**Authors:** Qingguang Zhang, Morgane Roche, Kyle W. Gheres, Emmanuelle Chaigneau, Ravi T. Kedarasetti, William D. Haselden, Serge Charpak, Patrick J. Drew

**Affiliations:** 1 Department of Engineering Science and Mechanics, The Pennsylvania State University, University Park, PA USA; 2 Institut National de la Santé et de la Recherche Médicale, U1128 Paris, France; 3 Laboratory of Neurophysiology and New Microscopies, Université Paris Descartes, Paris, France; 4 Graduate Program in Molecular Cellular and Integrative Biosciences, The Pennsylvania State University, University Park, PA USA; 5 Medical Scientist Training Program and Neuroscience Graduate Program, The Pennsylvania State University, University Park, PA USA; 6 Department of Neurosurgery and Department of Biomedical Engineering, The Pennsylvania State University, University Park, PA USA

**Keywords:** Neuro-vascular interactions, Cortex, Neurophysiology, Respiration

## Abstract

In the brain, increased neural activity is correlated with increases of cerebral blood flow and tissue oxygenation. However, how cerebral oxygen dynamics are controlled in the behaving animal remains unclear. We investigated to what extent cerebral oxygenation varies during locomotion. We measured oxygen levels in the cortex of awake, head-fixed mice during locomotion using polarography, spectroscopy, and two-photon phosphorescence lifetime measurements of oxygen sensors. We find that locomotion significantly and globally increases cerebral oxygenation, specifically in areas involved in locomotion, as well as in the frontal cortex and the olfactory bulb. The oxygenation increase persists when neural activity and functional hyperemia are blocked, occurred both in the tissue and in arteries feeding the brain, and is tightly correlated with respiration rate and the phase of respiration cycle. Thus, breathing rate is a key modulator of cerebral oxygenation and should be monitored during hemodynamic imaging, such as in BOLD fMRI.

## Abstract (web summary)

Understanding mechanisms of cerebral oxygen regulation is critical for healthy brain function. Here the authors show that respiration is a key modulator of cerebral oxygenation, which will be helpful in better resolving neurally-generated functional brain imaging signals, such as BOLD fMRI.

## Introduction

An adequate oxygen supply is critical for proper brain function^1^, and deficiencies in tissue oxygen is a noted comorbidity in human diseases^2^ and aging^3^. For these reasons, there has been a great deal of interest in studying dynamics of cerebral oxygenation^4–8^. However, there is a gap in our understanding of how behavior, such as natural exercises like locomotion, affects cerebral oxygenation. In natural environments, animals and humans have evolved to spend a substantial portion of their waking hours locomoting^9^. As exercise is known to have a positive effect on brain health^10^, a better understanding of the basic brain physiology accompanying these behaviors can give insight into how exercise can improve brain function. During movement, neuromodulator release and neural activity in many brain regions is elevated^11–14^, and there is an increase in cardiac output and respiratory rate. How these changes in local and systemic factors interact to control cerebral oxygenation is a fundamental question in brain physiology but is not well understood. Most cerebral oxygenation studies are performed in anesthetized animals^7,8,15,16^ (but see refs. ^4,17,18^), or non-invasively in humans. Anesthesia causes large disruptions of brain metabolism and neural activity^19^, and non-invasive human studies are impeded by technical issues, making accurate determination of any aspect of brain tissue oxygenation problematic.

In addition to the importance of understanding oxygen dynamics in the brain to basic physiology, a better grasp of natural oxygen dynamics in the brain will greatly aid in the interpretation of functional MRI signals^20^, which allow non-invasive imaging of neural activity. Previous work in awake primates has shown that tissue oxygen signals correspond well with changes observed with blood-oxygen level dependent (BOLD) fMRI^5^. Neurally-driven BOLD signals are generated by vessel dilation^21^. However, in addition to the BOLD fMRI signals of a neural origin (i.e., those generated by neurovascular coupling), BOLD signals can arise from sources that are not directly linked to underlying neural activity, such as pure vascular effects, respiration^22,23^, cardiac pulse rate, and autonomous hemodynamic regulation. Resolving the nature of non-neural sources of BOLD contrast has been an area of active research^23^, and will be helpful in better spatially resolving the neurally-generated BOLD signals^24^.

Here we investigated how and by what mechanisms voluntary exercise impacts brain tissue oxygenation. We used intrinsic optical signal (IOS) imaging^11,25^, electrophysiology, Clark-type polarography^5,16^, and two-photon phosphorescent dye measurement^4,7,8,17,18^ to elucidate how vasodilation, neural activity, and systemic factors combine to generate changes in brain oxygenation. All experiments were performed in awake mice that were head-fixed on a spherical treadmill^11,12^ or rotating disk^4,18,26^ that allowed them to voluntarily locomote. We find that cerebral oxygenation rises during locomotion in cortical regions that do not experience vasodilation, as well as when vasodilation is blocked. Oxygen levels increase in the arteries that supply the cortex during exercise, consistent with an increase in systemic oxygenation. Finally, oxygen fluctuations are correlated with spontaneous and locomotion-evoked changes in respiration rate, as well as the phase of the respiration cycle, also consistent with a dynamic regulation in systemic oxygenation.

## Results

### Locomotion drives localized cortical vasodilation

**Fig. 1:**
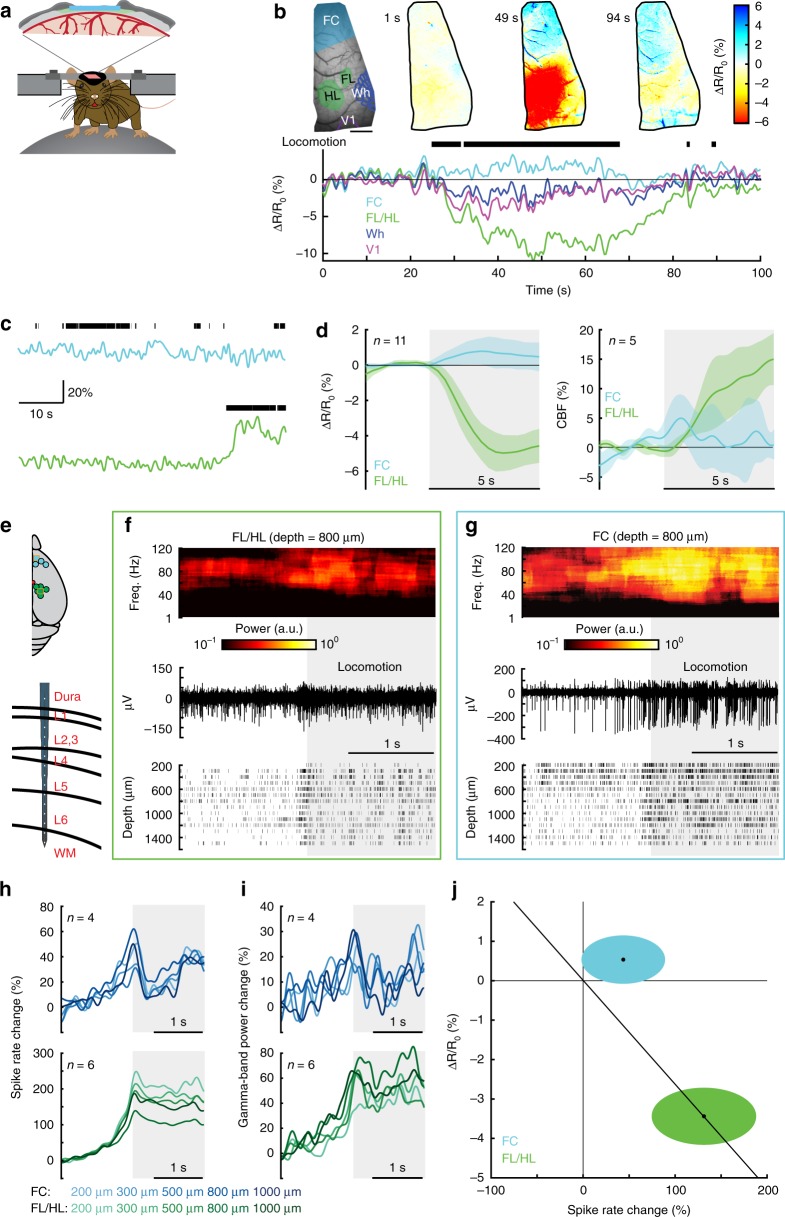
Locomotion drives cortical region specific hemodynamic and neural responses. **a** Experimental setup for IOS imaging. **b** Example showing CBV change during voluntary locomotion. Top left, an image of thin-skull window and corresponding anatomical reconstruction; scale bar = 1 mm. Top right, reflectance map before (1 s), during (49 s) and after (94 s) a voluntary locomotion event. Bottom, percentage change in reflectance (∆*R*/*R*_0_) during locomotion events for each brain region. Wh, vibrissae cortex; V1, visual cortex. **c** Example showing locomotion-evoked changes of CBF in FC (top) and FL/HL (bottom) in the same animal. **d** Population average of locomotion-triggered average of CBV (*n* = 11 mice, left) and CBF (*n* = 5 mice, right) responses in both FL/HL (green) and FC (blue). Data are shown as mean ± SD. **e** Top, all laminar electrophysiology measurement sites in FC (*n* = 4 mice) and FL/HL (*n* = 6 mice). The squares indicate the measurement sites showing in (**f**) and (**g**). Bottom, layout of the electrodes and measurement depth. **f** Example trial showing the large increase in gamma-band LFP power (top), raw signal (middle), and spike raster (bottom) during locomotion from a site 800 µm below the pia in FL/HL. Shaded area indicates the time of locomotion. **g** As in (**f**) but for FC. **h** Group average of locomotion-evoked spike rate responses in both FC (top, *n* = 4 mice) and FL/HL (bottom, *n* = 6 mice). **i** As in (**h**) but for locomotion-evoked gamma-band LFP power responses. **j** Changes of ∆*R*/*R*_0_, 2–5 s after the onset of locomotion plotted against spike rate change 0–2 s after the onset of locomotion in FL/HL (green ellipse) and FC (blue ellipse). For each ellipse, the radius along the vertical axis is the SD of ∆*R*/*R*_0_ across all 11 mice; the radius along the horizontal axis is the SD of spike rate across all animals (*n* = 4 for FC and *n* = 6 for FL/HL). The black dot in the center of each ellipse represents the average value of ∆*R*/*R*_0_ and spike rate response. The diagonal line shows the prediction of linear coupling.

We first assessed the spatial extent of cortical hemodynamic responses and their relationship to voluntary locomotion using IOS imaging^11,25^ (Fig. 1a). Imaging was done through a thin-skull window over the right-hemisphere (Fig. 1b). When the brain is illuminated with 530 nm light, reflectance decreases report dilations of arteries, capillaries and veins, which correspond with increases in cerebral blood volume (CBV). During locomotion, we observed region-specific changes in reflectance. There was a pronounced decrease in reflectance (corresponding to an increase in CBV) in forelimb/hindlimb representation of somatosensory cortex (FL/HL), while in frontal cortex (FC) there was no change, or a slight increase in reflectance (Fig. 1b). To better localize the area of decreased CBV, we used a smaller region of interest (ROI, 2–4 mm rostral and 0.5–2.5 mm lateral from bregma, ~4 mm^2^) more rostral in FC than in our previous study^11^. We also assayed cerebral blood flow (CBF) using laser Doppler flowmetry, which will evaluate flow changes in a ~1 mm^2^ area. The locomotion-evoked CBF showed similar spatial pattern of responses (Fig. 1c) as CBV. We quantified how locomotion affected both CBV and CBF in two complimentary ways. We calculated the locomotion-triggered average, generated by aligning the IOS or laser Doppler signals to the onset of locomotion (see “Methods”) using only locomotion events ≥ 5 s in duration (Fig. 1d). The locomotion-triggered average showed no significant change in CBF in FC (*n* = 5 mice, Wilcoxon signed-rank test, *p* = 0.22), and a large increase in FL/HL (*n* = 5 mice, Wilcoxon signed-rank test, *p* = 0.03). The locomotion-triggered average showed significant increase in optical intensity in FC (*n* = 11 mice, Wilcoxon signed-rank test, *p* < 0.001) while decrease in optical intensity in FL/HL (*n* = 11 mice, Wilcoxon signed-rank test, *p* = 0.0122). We also calculated the hemodynamic response function (HRF)^25,27^, which is the linear kernel relating locomotion events to observed changes in CBV and CBF (Supplementary Fig. 1), using all locomotion events. When we quantified the net CBF using the HRFs, locomotion actually drove a significant decrease in flow in FC and increase in FL/HL (Supplementary Fig. 1). Using the HRFs to quantify the net CBV, we obtained same conclusions as derived from locomotion-triggered average. This shows that locomotion and the accompanying cardiovascular changes do not drive global increases in CBF/CBV, rather CBF/CBV increases are under local control. This lack of non-specific flow increase in the cortex during locomotion is likely because of autoregulation of the feeding arteries at the level of the circle of Willis and larger resistance arteries, as well as increased blood flow to the muscles^28^.

To assess neural activity during locomotion, we measured the local-field potential (LFP) and multi-unit activity (MUA) in a separate group of seven mice (six sites in FL/HL and four sites in FC) using multi-channel linear electrodes (Fig. 1e). We used electrophysiological measures of neural activity, as they are more sensitive than calcium indicators (which fail to detect about half of the spikes even under ideal conditions^29^), and do not disrupt normal neural activity as genetically encoded calcium indicators can do. Since gamma-band (40–100 Hz) LFP power has been observed to be the strongest neural correlate of hemodynamic signals in rodents^25,30^, primates^5^ and humans, and increases in gamma-band activity are also closely associated with the increases in metabolic demand^31^, we quantified how locomotion affects neural activity by generating locomotion-triggered averages of gamma-band LFP power and spiking (see “Methods”). We observed that both the gamma-band LFP power and spike rate increased during locomotion across all layers in both FL/HL (Fig. 1f, h, i) and FC (Fig. 1g–i). The slow rise in neural activity a few hundred milliseconds before the onset of locomotion is due to low-pass filtering of the MUA signal (5 Hz, see “Methods”) and windowing (1 s duration) required to estimate the LFP power^21^, as well as the ramping up of neural activity due to arousal changes seen before voluntary locomotion^32^. As optogenetic stimulation of fast spiking inhibitory neurons has been shown to induce large increases in blood oxygenation in somatosensory cortex^33^, we sorted recorded spikes into fast spiking (FS, putatively inhibitory) and regular spiking (RS, putatively excitatory) spikes (see “Methods”). We found that FS and RS neurons exhibited a similar degree of rate increases during locomotion in both FL/HL and FC areas (Supplementary Fig. 2).

Taken together, our results show that a short bout of locomotion increases neural activity, which is followed by an increase in CBV and CBF in FL/HL, and a small decrease or no change in CBV and CBF in FC. Together with our previous work^11,34^, these results suggest that coupling between neural activity and hemodynamics are brain region-specific (Fig. 1j), as seen in many other neurovascular coupling studies in the cortex and other brain regions^35^. The lack of observed vasodilation in the FC is not due to a lack of sensitivity of our IOS imaging paradigm, as if the vasodilation in FC had the same relationship to neural activity as in FL/HL, we would expect to see a 2% decrease in the reflectance (Fig. 1j), which is easily detectable with our IOS setup^25^. As tissue oxygenation reflects the balance between oxygen supply and utilization^36^, we would expect that in FL/HL, increased activity of the neurons will be more than matched by an increased blood supply, leading to an increase in tissue oxygenation. However, increased neural activity in FC during locomotion will not be matched by an increase in the blood supply and should lead to a decrease in oxygenation in FC.

### Locomotion drives cortex wide increases in brain oxygenation

**Fig. 2:**
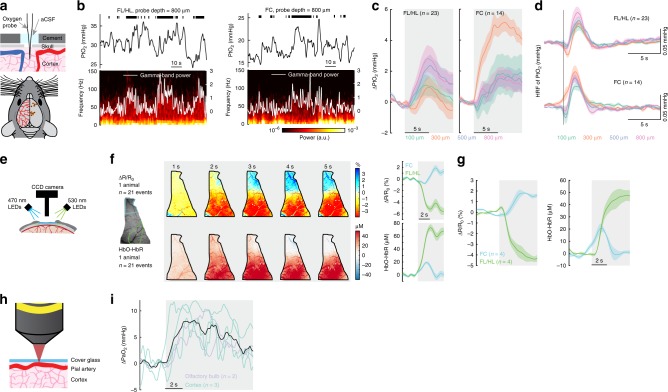
Cortex-wide increases in oxygenation during locomotion. **a** Top, experimental setup. Bottom, measurement sites. **b** Example traces showing PtO_2_ responses to locomotion at sites 800 µm below brain surface in FL/HL (left) and FC (right). Top, black ticks denote binarized locomotion events; Middle, PtO_2_ responses to locomotion; Bottom, example of data showing spectrogram of LFP (white trace showing the gamma-band LFP power). **c** Locomotion-evoked cortical tissue oxygenation increases (∆PtO_2_) at all measured depths in both FL/HL (left, *n* = 23 mice) and FC (right, *n* = 14 mice). Gray shaded area indicates locomotion. **d** HRF of tissue oxygenation at different depths in both FL/HL (top, *n* = 23 mice) and FC (bottom, *n* = 14 mice). Vertical black line showing the start of a brief impulse of locomotion. **e** Schematic showing the optical imaging spectroscopy setup. **f** Left, example data showing spatial distribution of locomotion-evoked response of ∆*R*/*R*_0_ and HbO-HbR in an example mouse. Right, locomotion-triggered average of ∆*R*/*R*_0_ and HbO-HbR for the same mouse in FC (blue) and FL/HL (green). **g** Group average of locomotion-evoked response of ∆R/R_0_ and HbO-HbR in FC (*n* = 4 mice) and FL/HL (*n* = 4 mice). **h** Schematic showing the measurement of PaO_2_ using 2PLM. **i** Locomotion induced PaO_2_ increases in five arteries (three in the cortex (green) and two in the olfactory bulb (purple)) from a total of four mice. Mean response of all arteries is shown as a black line. Solid lines and shaded area in (**c**), (**d**), (**f**) and (**g**) denote mean ± SEM, respectively.

To test if the brain region-dependent differences in neurovascular coupling drove regional differences in brain oxygen dynamics during locomotion, we measured partial pressure of tissue oxygen (PtO_2_) in awake, behaving mice (*n* = 37 mice, 23 in FL/HL, and 14 in FC; 148.2 ± 28.3 min of recording for each mouse) using Clark-type polarographic electrodes (Fig. 2a). Signals from these electrodes are similar to those obtained with BOLD fMRI^5^, but with sub-second response time (Supplementary Fig. 3a, b), long-term stability (Supplementary Fig. 3c, d) and higher spatial resolution. We measured oxygen dynamics at different cortical depths by sequentially advancing the probe from the cortical surface into deeper layers. We observed a laminar-dependence of resting PtO_2_ in awake mice, with lower oxygenation in layer I than other layers in both FL/HL and FC (Supplementary Fig. 4a, b, see also refs. ^4,17^), though this is probably too thin a section of brain to be distinguishable with laminar fMRI. Resting PtO_2_ levels were similar at each cortical depth in both FL/HL and FC (Supplementary Fig. 4a, b). No difference in onset time or peak time was observed in the HRFs of PtO_2_ across layers, though the onset time was shorter in FC (Supplementary Fig. 4d, e), consistent with fMRI measurements using ultrashort stimuli^37^. Note that because we are measuring tissue oxygenation in this case, any oxygen dynamics in the vasculature will be temporally blurred by the diffusion dynamics of oxygen in the tissue. These results, together with the observation that resting PtO_2_ is similar in somatosensory cortex and olfactory bulb glomerular layer^4^, indicate that the spatial distribution of oxygen in the brain under normal (non-anesthetized) physiological condition is relatively homogenous. This quantification of laminar tissue oxygen dynamics should aid interpreting the complicated dynamics of laminar fMRI signals^38^.

Locomotion produces large, sustained dilation of arteries^39^ and increases in CBF and CBV^11,34^ in somatosensory cortex. These locomotion-induced dilations were not due to systemic effects, as they have been shown to be unaffected by drugs that do not cross the blood brain barrier (BBB) that increase or decrease the heart rate^40^ and are blocked by the suppression of local neural activity^25^. The locomotion-induced dilations are comparable in magnitude to those elicited by episodic whisker stimulation^25^ which is known to elevate oxygenation, so one would expect increases in tissue oxygenation in FL/HL during locomotion. As anticipated, we observed increases in PtO_2_ during locomotion in FL/HL in all layers (Fig. 2b–d). Because the supply of blood to FC does not increase, but neural activity does, one would expect a decline in tissue oxygenation during locomotion. Surprisingly, we also observed a very similar PtO_2_ increase in FC (Fig. 2b–d) to that observed in FL/HL, despite small decreases in CBV or CBF, and an increase in neural activity. The elevation of PtO_2_ in FC during locomotion suggests that other factors can increase oxygenation in the brain.

Polarographic probes provide measures of oxygen tension over a small region of brain tissue, and the response may be affected by vasculature type and density^41,42^ surrounding the probe. To distinguish compartment-specific oxygen tension in the tissue, arterial and venous blood spaces, we then mapped the spatial distribution of locomotion-evoked brain oxygenation response using optical imaging spectroscopy^43^ (Fig. 2e). Taking advantage of differences in the optical absorption spectra of oxyhemoglobin (HbO) and deoxyhemoglobin (HbR)^43^, we collected reflectance images during rapid alternating green (530 nm) and blue (470 nm) illumination. Note that spectroscopic measurements report oxygen concentrations in red blood cells, while polarography reports average oxygen concentration in tissue near the electrode. The oxygen levels in the tissue will differ from that in the blood somewhat due to the constraints of oxygen diffusion from the blood into the tissue and ongoing metabolic processes in neurons and glial cells. Using cerebral oxygenation index (HbO-HbR)^44^, the spectroscopic measures of hemoglobin oxygenation were similar to measurements from the tissue using polarographic probes: both methods yielded an increase in oxygenation during locomotion in both FC and FL/HL (Fig. 2f, g). These oxygenation changes persisted even when the heart rate increase associated with locomotion was pharmacologically blocked or occluded (Supplementary Fig. 5b, d, e), indicating they were not driven by increased cardiac output during locomotion.

Moreover, locomotion-induced elevations in oxygenation were present in the parenchyma, arterial and venous blood (Fig. 2f). As oxygen levels in the brain strongly depends on the arterial oxygen content^8^, we made direct measurements of oxygen partial pressure in the center of pial arteries (PaO_2_) using two-photon phosphorescence lifetime microscopy (2PLM, Fig. 2h)^4,7,8,17,18^, with a new phosphorescent probe (Oxyphor 2P) which has a very high brightness, improving measurement speed and imaging depth^45^. We asked if the oxygen levels increased in the center of large pial arteries that supply blood to the brain. As blood in these arteries will have minimal time to exchange oxygen in their transit through the heart and carotid artery to the brain, oxygen levels in these arteries will track systemic oxygenation levels. We measured PaO_2_ in cortical and olfactory bulb arteries and found that PaO_2_ increased during locomotion (Fig. 2i). Taken together, these measurements are consistent with an increase in systemic blood oxygenation that leads to a brain-wide increase of oxygenation in the tissue and vascular compartments during locomotion. The increase in oxygenation accompanying the decrease in CBV and CBF in FC suggests that neurovascular coupling is not the only process controlling brain oxygenation^46^ during locomotion.

### Cortical oxygen increases even when vasodilation is blocked

**Fig. 3:**
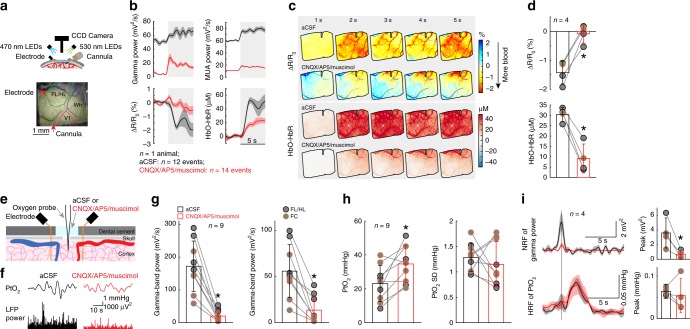
Locomotion-evoked cortical oxygenation increases persist when vasodilation is blocked. **a** Experimental setup for spectroscopy. **b** Locomotion-evoked gamma-band LFP power (top left), MUA power (top right), ∆R/R_0_ (bottom left) and HbO-HbR (bottom right) in one representative mouse following aCSF (black, 12 locomotion events) and CNQX/AP5/muscimol (red, 14 locomotion events) infusion. **c** Locomotion-evoked spatial distribution of ∆R/R_0_ (top) and HbO-HbR (bottom) for the same mouse shown in (**a**) and (**b**) following aCSF and CNQX/AP5/muscimol infusion. **d** Group average of locomotion-evoked ∆R/R_0_ (top, * paired *t*-test, *t*(3) = 7.4235, *p* = 0.0051) and HbO-HbR (bottom, * paired *t*-test, *t*(3) = 8.0007, *p* = 0.0041) after aCSF or CNQX/AP5/muscimol infusion in four mice. The orange circle denotes the mouse shown in (**b**) and (**c**). **e** Experimental setup for simultaneous PtO_2_ and LFP measurements. **f** Example of resting PtO_2_ fluctuations (top) and resting gamma-band LFP fluctuations (bottom) in FL/HL in a single mouse. **g** Comparison of spontaneous LFP activity (left, * Wilcoxon signed-rank test, *p* = 0.0039) and fluctuations (SD, right, * paired *t*-test, *t*(8) = 5.0246, *p* = 0.0010) before (black) and after (red) application of CNQX/AP5/muscimol in FL/HL (4 mice, black circle) and FC (five mice, orange circle). **h** As (**g**) but for spontaneous PtO_2_ activity (left, * paired *t*-test, *t*(8) = 3.2712, *p* = 0.011) and fluctuations (SD, right, * paired *t*-test, *t*(8) = 0.7542, *p* = 0.4723). **i** Top left, NRF of gamma-band LFP power (*n* = 4 mice, 1 in FL/HL and 3 in FC) before and after application of CNQX/AP5/muscimol. Vertical black line indicates the start of a brief impulse of locomotion. Bottom left, as in top left but for HRF of PtO_2_. Top right, peak amplitude of NRF of gamma-band LFP power before and after application of CNQX/AP5/muscimol (*paired *t*-test, one sided, *t*(3) = 3.4299, *p* = 0.0208). Bottom right, as in top right but for peak amplitude of HRF of PtO_2_ (paired *t*-test, *t*(3) = 0.5861, *p* = 0.599). Solid lines and shaded area in (**b**) and (**i**) denote mean ± SEM, respectively. Data are shown as mean ± SD in all other graphs.

Our observation that locomotion induced localized blood flow/volume increases, but cortical-wide increases in brain oxygenation, led us to hypothesize that activity-dependent vasodilation may not be necessary for an increase in oxygenation. To test this, we pharmacologically blocked glutamatergic and spiking activity by infusing/superfusing a cocktail of 6-cyano-7-nitroquinoxaline-2,3-dione (CNQX, 0.6 mM), (2R)-amino-5-phosphonopentanoic acid (AP5, 2.5 mM) and muscimol (10 mM) to suppress local neural activity. We first infused a cocktail of CNQX/AP5/muscimol via a cannula into FL/HL^25^, while concurrently monitoring neural activity, CBV and blood oxygenation (*n* = 4 mice, Fig. 3a). The cocktail infusion suppressed resting gamma-band LFP power by 80 ± 12% and spiking activity by 82 ± 3% relative to vehicle infusions. Similarly, the standard deviation (SD) of these neural activity measurements during resting periods, an indicator of spontaneous neural activity levels, was decreased by 75 ± 18% in the gamma-band LFP power and by 85 ± 6% in the MUA amplitude. To quantify CBV responses, we selected a semicircular ROI centered on the cannula and with a radius specified by the distance between the electrode and cannula (Fig. 3a), to ensure the ROI only included suppressed cortex^25^. Accompanying this neural activity blockade, baseline reflectance from the ROI increased (indicating decreased CBV), and the locomotion-evoked decrease in reflectance (vasodilation) was almost completely suppressed (Fig. 3b–d), consistent with our previous study^25^ showing that intracerebral infusion of a cocktail of CNQX/AP5/muscimol suppressed sensory-evoked CBV increase. However, the block of neural activity was less effective during locomotion (Fig. 3b), likely due to the large increases of neural and modulatory drive to the cortex that occur during locomotion^13,14^. Nevertheless, this is conducive for testing our hypothesis, as a complete block of vasodilation and an incomplete block of neural activity increases should lead to a decrease in oxygenation. However, if there is no oxygenation decrease, or the oxygenation increases, this would indicate that the oxygenation of the inflowing blood is elevated during locomotion. When locomotion-induced vasodilation was blocked, the locomotion-evoked increase in HbO-HbR persisted, though the increase was smaller (Fig. 3b–d, Supplementary Fig. 5b, c, e). This increase was surprising, as we were able to completely block the locomotion-induced vasodilation, and there was still a small locomotion-induced increase in neural activity, which should result in a net decrease in oxygenation.

We further studied the effects of the suppressed vasodilation on oxygen responses in the tissue in a separate set of mice using polarographic electrodes (*n* = 9 mice, 5 in FC and 4 in FL/HL). We topically applied a cocktail of CNQX/AP5/muscimol to the cortex, while measuring spontaneous and locomotion-evoked neural activity and PtO_2_ in the superficial cortical layers (100–200 µm below the pia). The efficacy of the cocktail in suppressing neural activity was monitored with two electrodes spanning the oxygen measurement site^25,30^ (Fig. 3e). Similar to intracortical infusions, superfusing the cocktail potently suppressed resting gamma-band LFP power by 89 ± 8% and the SD by 77 ± 21% (Fig. 3g). Resting PtO_2_ increased by ~70% following the suppression of neural activity and vasodilation (before: 23.09 ± 10.60 mmHg; after: 34.64 ± 11.11 mmHg; Fig. 3h), consistent with neural signaling being a major component of metabolic demand^47^. To quantitatively assay locomotion-evoked oxygen and neural responses, we calculated the linear kernels (i.e., the HRF) relating PtO_2_ and the gamma-band LFP power to locomotion. To ensure that vasodilation was blocked, we only analyzed those animals (*n* = 4 mice, 3 in FC and 1 in FL/HL) that showed >50% suppression of locomotion-evoked neural activity. In these animals, application of CNQX/AP5/muscimol reduced peak amplitude of gamma-band LFP neural response function (NRF) by 81 ± 8% (Fig. 3i). If activity-dependent vasodilation is the only determinate of tissue oxygenation, we would expect the HRF of PtO_2_ shows profound reductions, since the vasodilation was blocked by the suppression of neural activity (Fig. 3b–d). However, the peak amplitude of PtO_2_ HRF was not changed (82 ± 51% of before cocktail application, Fig. 3i). Taken together, these results show that suppressing vasodilation does not block the locomotion-evoked oxygen increases.

### Respiration drives changes in tissue and blood oxygenation

**Fig. 4:**
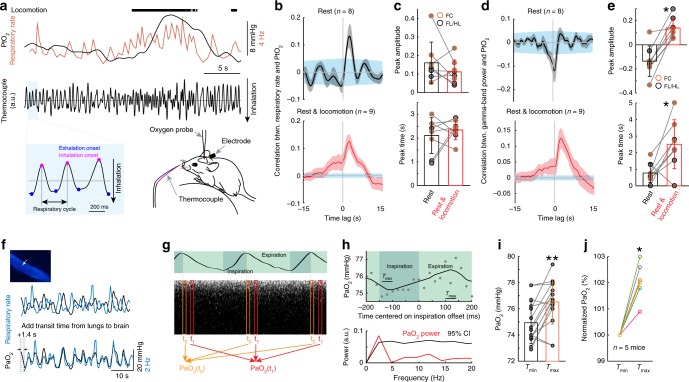
Respiration drives changes in cerebral tissue and arterial blood oxygenation. **a** Measuring respiration using a thermocouple. **b** Cross-correlation between PtO_2_ and respiratory rate during rest (top) and periods including rest and locomotion (bottom). **c** Peak amplitude (top, Wilcoxon signed-rank test, *p* = 0.3125) and peak time delay (bottom, Wilcoxon signed-rank test, *p* = 0.7422) of cross-correlation between PtO_2_ and respiratory rate during rest (black) and periods including rest and locomotion (red). **d** As (**b**) but for correlation between PtO_2_ and gamma-band LFP power. **e** As (**c**) but for peak time (* paired *t*-test, *t*(7) = 6.1918, *p* < 0.001) and peak time delay (* Wilcoxon signed-rank test, *p* = 0.0234) of cross-correlation between PtO_2_ and gamma-band LFP power. **f** Example data showing the temporal relationship between respiratory rate (black) and PaO_2_ (blue) in the center of one artery (white arrow) in somatosensory cortex during rest. **g** Schematic showing measurement of PaO_2_ fluctuations driven by respiration cycle. **h** Top, PaO_2_ change in one artery during the respiratory cycle at rest. Each filled circle denotes averaged PaO_2_ over a 20 ms window aligned to a specific phase of the respiration cycle and averaged over 15 recordings. The solid curve shows filtered data (first-order binomial filter, five repetitions). *T*_min_ and *T*_max_ denote the time period (40 ms) PaO_2_ reaches minimum and maximum, respectively. Bottom, power spectrum of PaO_2_ (red) and 95% confidence interval (CI, black) given by randomizing the phase of the PaO_2_ signal. The PaO_2_ power at the respiratory frequency (~2.5 Hz) is significantly greater than the 95% CI level. **i** PaO_2_ at *T*_max_ and *T*_min_ for the 15 recordings from the artery shown in (**h**). ** Wilcoxon signed-rank test, *p* < 0.01. **j** Normalized PaO_2_ at *T*_min_ and *T*_max_ for six vessels (three in cortex, and three in olfactory bulb, *n* = 5 mice) with statistically significant PaO_2_ power spectrum peaks at the respiratory frequency. * Wilcoxon signed-rank test, *p* = 0.0313. Data are shown as mean ± SEM in (**b**) and (**d**), and ± SD in all other graphs. Blue shaded region in (**b**) and (**d**) shows 95% CI of cross-correlation.

One possible driver of the increases in cerebral oxygenation is the increase in respiration during locomotion. Changes in respiration affect blood oxygen levels in the carotid artery^48,49^ in anesthetized animals, and in humans, inhalation of 100% oxygen can elevate brain oxygen levels^50^. However, it is not known if normal fluctuations in respiration rate can impact cerebral oxygenation during normal behaviors. We tested whether respiration was correlated with oxygenation during locomotion by simultaneously measuring cortical tissue oxygenation and respiration (Fig. 4a). Locomotion was accompanied by a robust increase in respiratory rate (Fig. 4a, Supplementary Fig. 6), and fluctuations in respiratory rate on the time scale of seconds were linked to fluctuations in PtO_2_ (Fig. 4a). We quantified how well the fluctuations of respiratory rate and gamma-band LFP power (which has been shown to be the LFP band most correlated with vasodilation^25,30^) correlated with the fluctuations in PtO_2_ by calculating the cross-correlation. During periods of rest, increases in gamma-band LFP power were correlated with decreased oxygenation (Fig. 4d, e), which was unexpected as gamma-band power increases during rest are correlated with vasodilation^25,30,51^. Because the decrease takes place with near zero time lag (Fig. 4d, e), it seems as though the dilation induced by spontaneous neural activity are insufficient relative to the metabolic demand. In contrast, respiration rate increases were correlated with increased oxygenation with a slight delay, consistent with the transit time of the blood from the lungs to the brain (Fig. 4b, c). When periods of locomotion were included, the correlation between gamma-band LFP power and oxygenation was positive, suggesting that the coupling depends on animal’s state^51^ (Fig. 4d, e). The coupling between other frequency bands of the LFP and oxygen increases was negative (Supplementary Fig. 7), consistent with previous reports showing decreases in the power of these bands during voluntary locomotion^32^ (see also Supplementary Fig. 8). Because cortical excitability and respiratory rate are correlated during locomotion (likely due to the reciprocal connections between respiratory and modulatory regions^52^), we sought to disentangle their respective contributions to cerebral oxygenation using partial coherence analysis^53^. We found that the coherence between respiratory rate and PtO_2_ was not due to the co-varying neural component (Supplementary Fig. 9b), nor was the coherence between gamma-band power and PtO_2_ affected by removing the respiratory rate contribution (Supplementary Fig. 9c). Thus, the partial coherence analysis indicates that respiration and neural activity (and likely vasodilation) affect tissue oxygenation independent of each other.

The correlated fluctuations in respiratory rate and PtO_2_ suggests that the oxygen tension of arterial blood should also track the respiratory rate. To test this, we simultaneously monitored respiration and PaO_2_ in the pial arteries using 2PLM. In mice with irregular respiration, where respiratory rate transients of a few seconds occurred without locomotion, PaO_2_ followed respiration rate fluctuations (Fig. 4f), showing that changes in respiration rate can alter the oxygenation of the arterial blood entering the cortex.

We then asked if PaO_2_ tracked the phase of respiration, that is, whether the concentration of oxygen in the blood entering the brain fluctuated in phase with the inspiration-expiration cycle. This requires measuring PaO_2_ at rates high enough (>5 Hz) to capture fluctuation in PaO_2_ due to respiration (nominally 2.5 Hz). As measurement of PaO_2_ with the 2PLM method is based on the lifetime of the phosphoresce decay of the dye, accurate quantification of the oxygen concentration requires averaging of decays^45^, which amounted to ~3000 decays at our laser power (corresponding to ~0.75 s of data), too slow to capture inspiration-expiration linked changes in PaO_2_. Therefore, we took advantage of the respiration periodicity. When the respiratory rate is very regular, the phosphorescence decays can be aligned and binned according to their place in the phase of the respiratory cycle (Fig. 4g), analogous to how erythrocyte-related transients can be detected in capillaries^4,7^, or analyzing the signal in the frequency domain. In animals (*n* = 5) with long bouts of highly regular respiration rate (average frequency 2.5 Hz, SD ≤ 0.6 Hz, average frequency to SD ratio > 4), we tested whether fluctuations of PaO_2_ tracked the respiratory cycle with a phase randomization test (see “Methods”). Six arteries (three in cortex, and three in olfactory bulb) out of nine had significant PaO_2_ fluctuations synchronized with the respiratory cycle (Fig. 4h–j). Note that even though three arteries did not pass the phase randomization test and were excluded, the plot in Fig. 4j remained significant when including all nine arteries (Wilcoxon signed-rank test, *p* = 0.0039). These arteries showed oscillations in PaO_2_ at the frequency of respiration that were significantly larger than would be expected by chance (reshuffling test, see “Methods”). This shows that arterial blood flowing to the brain is not saturated at rest. It also shows that oxygen tension in blood tracks sub-second respiration dynamics, so increase in respiration can drive rapid increases in systemic blood oxygenation that will impact brain oxygenation.

These results above indicate that the blood was not saturated with oxygen at rest. This suggests that changing the availability of oxygen could change the saturation of arterial oxygen, even without locomotion or a change in respiration. In humans, arterial oxygenation can be elevated through an oxygen challenge, where the subject inhales a gas mixture with elevated oxygen levels^54^. We addressed this using an oxygen challenge (see “Methods”). During periods of rest, in both FL/HL (*n* = 2 mice) and FC (*n* = 2 mice), inhaling 100% oxygen substantially increased oxygenation of cerebral blood (Supplementary Fig. 10), as seen in human fMRI. Importantly, during oxygen challenge, there were no significant changes in respiration rate or behavior of the mouse (Supplementary Fig. 10). Taken together, these results show that systemic blood oxygenation increases in mice, from either respiration increase or environmental oxygen availability, can drive increases in brain oxygenation.

### Modeling respiratory contribution to cortical oxygenation

**Fig. 5:**
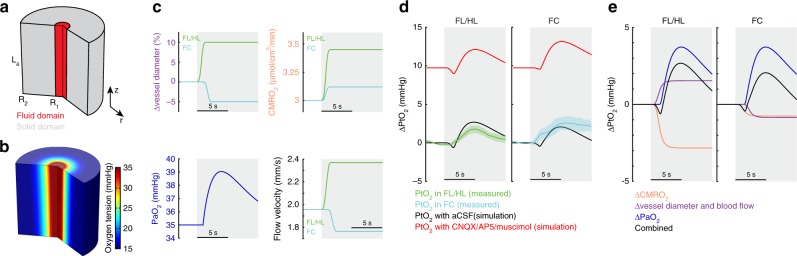
Tissue oxygenation during locomotion depends on the interplay of arterial oxygenation, CMRO_2_ and vasodilation. **a** Schematic showing the model geometry and boundary conditions. The 2D axisymmetric model consists of two domains, the fluid (or blood) domain, and the solid (or tissue) domain. The axial (z) length of both the domains is L_a_. The fluid domain has an outer radius of R_1_ and the solid domain has an outer radius of R_2_. A pressure difference across the length of the blood vessel and an imposed vasodilation profile are used to change the vessel diameter and the fluid velocity simultaneously. The arterial oxygen concentration is imposed as a boundary condition at the vessel inlet. **b** The oxygen tension in the blood vessel and surrounding tissue under rest (stationary) conditions. **c** Simulated locomotion-induced changes in vessel diameters in FL/HL and FC (top left), cerebral metabolic rate of oxygen consumption (CMRO_2_) in FL/HL and FC (top right), blood flow velocity at the centerline of the vessel (bottom left), and arteriole oxygen tension (PaO_2_, bottom right). **d** Simulated effects of inhibiting neural activity using CNQX/AP5/muscimol on locomotion-evoked PtO_2_ change (∆PtO_2_) in both FL/HL (left) and FC (right). Green and cyan shaded area denote one SEM of measured PtO_2_ change in FL/HL and FC, respectively. **e** Decomposition of locomotion-evoked oxygen changes in FL/HL (left) and FC (right). In both regions, changes in arterial oxygenation strongly influence tissue oxygenation.

We then asked what the relative contributions of increased arterial oxygenation and vasodilation were to changes in PtO_2_. Recent work has shown that substantial oxygenation exchange occurs not only at capillaries, but also around penetrating arterioles in the cortex^6,8,17^. To better understand how increases in blood oxygenation impact tissue oxygenation around arterioles, where the simple geometry of the vasculature allows us to better capture the dynamics of oxygenation changes due to vasodilation and systemic oxygenation changes, we simulated oxygen transport and consumption in blood and the brain parenchyma (Fig. 5a). In this model, we dynamically varied arterial oxygenation, cerebral metabolic rate of oxygen (CMRO_2_), vessel diameter and blood flow. For this model, we used experimentally-determined quantities for the values of arterial oxygenation, and vessel diameter dynamics. We used published values for CMRO_2_ (Supplementary Table 1) for these simulations (Fig. 5c). The free parameters were chosen such that tissue oxygenation predicted by the model matched our oxygen measurements in FC and FL/HL (Fig. 5d). Consistent with our data (Fig. 3h), the model also showed an increase in tissue oxygenation when neural activity (and metabolism) were suppressed (Fig. 5d). Moreover, using this model, we were able to tease out the relative contributions of vasodilation and increased arterial oxygenation to tissue oxygenation changes in both FC and FL/HL. In FL/HL, the large increase in CMRO_2_ during locomotion were counteracted by approximately equal increases in arterial oxygenation due to vasodilation and increase in arterial oxygenation. In FC, the small increase in CMRO_2_ and vasoconstriction was totally offset by the increase in arterial oxygenation (Fig. 5e). These simulations show that both increases in respiration and vasodilation (with the accompanying increase in blood flow) contribute to modulating tissue oxygenation in the cortex during behavior. Next, we asked if the increase in FC oxygenation could be caused by a decrease in CMRO_2_ during locomotion. In this case, the increase in oxygenation could be alternatively explained by a 15% reduction in CMRO_2_ (Supplementary Fig. 11), which is only likely in deep sleep or under anesthesia^19^. A recent study has shown that elevations of neural activity are highly correlated with increases of CMRO_2_^55^, which suggests that a decrease in CMRO_2_ accompanying an increase in neural activity is highly unlikely. These simulations show that increased arterial oxygenation that accompanies increases in respiration can lead to increases in tissue oxygenation, even in brain regions that lack a marked hyperemic response. In addition, the increase in arterial oxygenation will also increase the oxygen tension in the tissue around the capillary bed^56^, though the actual changes will depend on the details of the capillary geometry and the movement of individual red blood cells, which is hard to capture without detailed anatomical models, and will depends on the details of flow dynamics. Taken together, the experimental data and the simulation support the notion that increases in respiratory rate play an important role in regulating cerebral oxygenation.

## Discussion

Respiration is not the only physiological change that accompanies exercise, and it bears considering other mechanisms that could account for the cerebral and arterial oxygenation changes seen here. Exercise causes large changes in cardiac output and blood pressure, and can be accompanied by changes in blood CO_2_ and lactate levels, but we think they are unlikely to be the cause of the nonspecific increase in cerebral oxygenation that we saw here. First, for increases in cardiac output to raise global oxygenation in the cortex (independent of any changes in systemic oxygenation), it would need to drive an increase in CBF. Our laser Doppler experiments show that CBF does not rise in FC, as they are likely buffered by resistance arterioles and autonomic regulation of the circle of Willis^57^ (but see ref. ^58^). Additionally, when heart rate and blood pressure increases during locomotion were blocked (with the beta blocker atenolol, which does not cross the BBB) or occluded (with the muscarinic receptor antagonist glycopyrrolate which also does not cross the BBB), there was no change in the locomotion-evoked CBV change (Supplementary Fig. 5, see also ref. ^40^). Therefore, systemic cardiac output increase cannot explain the increases in cerebral oxygenation seen during locomotion. Second, while CO_2_ is a strong vasodilator, and can drive increases in cerebral oxygenation under hypercapnia conditions by dilating blood vessels, rodents become hypocapnic during sustained exercise^59^. Exercise-evoked changes in CO_2_ would tend to cause cerebral vasoconstriction and would tend to drive a deoxygenation. Again, this mechanism could not drive the observed increase in blood and tissue oxygenation in FC without corresponding flow increases and vasodilation. Sustained, high intensity exercise can cause increases in blood lactate over tens of minutes^60^, but there is no way that these lactate changes could drive changes in cerebral oxygenation seen in our experiments on the time scale of seconds. So, while many systemic variables change during voluntary locomotion, with the exception of increases in respiration rate, none would be able to increase oxygen in the arteries or in tissue within a few seconds of locomotion onset, nor could they explain the breathing cycle-locked oscillations in the blood oxygenation or respiration-related fluctuations at rest. Thus, unless there is some heretofore unknown physiological process taking place during exercise, the most parsimonious explanation is that increases in respiration are the origin of the oxygenation increase in the brain observed here.

While our studies were performed in mice, there are respiration-driven fluctuations in the arterial blood of ungulates^48,49^, suggesting it is a general property of mammals. Our finding may have useful implications for human fMRI work. Though the effects of respiration rate on CO_2_ levels (which will cause changes in arterial diameter and blood flow on the scale of tens of seconds to minutes) have been appreciated in human neuroimaging^22,23^, changes in systemic oxygenation due to respiration changes will be more rapid (of the order of a few seconds). While it is generally presumed that arterial blood is saturated in humans^36^ (but see ref. ^15^), arterial oxygen tension decreases substantially with age^61^ and acutely during sleep^62^, and oxygen challenge in humans and monkeys^63,64^ raises blood oxygenation just as in our mice (Supplementary Fig. 10). This suggests that respiration may play a more important role in cerebral oxygenation in humans than is currently appreciated, particularly as respiration rate is actively modulated during cognitive tasks^65^. Respiration in humans is known to be increased following auditory or visual stimulation, and patterns of respiration differ from individual to individual, which might play a role in cerebral oxygen dynamics^65^. Recent work has shown that respiration is actively modulated during cognitive tasks in humans, and respiration dynamics predict task performance^66^. There is an emerging consensus that global brain activity is coordinated with respiration phase and rate in both animals and humans^67,68^. Because respiration will be modulated by tasks, there may be spurious, spatially distributed, non-neuronal BOLD signals locked to the stimulus driven by respiration changes. Fortunately, these artifacts can be removed by regressing out the effects of respiration, either using measures of systemic blood oxygenation, or measures of respiration itself^22,23^.

The role of increased respiration in increasing brain oxygenation during behavior observed here is likely facilitated by the reciprocal connections between respiratory centers and the locus coeruleus^52^ and other brain regions involved in arousal^69^. Consistent with a tight interplay between respiration and metabolic demand in the brain, activation of the locus coeruleus, which will cause increases in alertness, and also causes concomitant increases in neural activity and blood flow in the cortex^70^. This tight interplay at the behavioral and anatomical levels between cortical arousal and respiration may help maintaining healthy oxygenation for optimal cortical function.

## Methods

### Experimental design

Cerebral oxygenation, laminar electrophysiology, CBF and CBV data were acquired from separate groups of awake, behaving mice during voluntary locomotion. All experimental procedures were approved by the Pennsylvania State University and INSERM Institutional Animal Care and Use Committee guidelines.

### Animals

A total of 78 C57BL/6J mice (58 male and 20 female, 3–8 months old, 25–35 g, Jackson Laboratory) and 5 Thy1-GCaMP6f mice (4 male and 1 female, 3–12 months old, 25–35 g, Jackson Laboratory) were used. Recordings of laminar cortical tissue oxygenation were made from 37 mice [23 (13 male and 10 female) in FL/HL and 14 (7 male and 7 female) in FC] using Clark-type polarographic microelectrode. Simultaneous measurements of cortical tissue oxygenation using polarographic electrodes, respiration and local field potential were conducted in 9 mice [5 (4 male and 1 female) in FL/HL and 4 (2 male and 2 female) in FC]. Six of these mice were also used for laminar cortical tissue oxygenation measurements. Local field potential and spiking activity of different cortical layers were measured using laminar electrodes in a separate set of 7 male mice (4 in FC and 6 in FL/HL, 3 mice were measured in both FL/HL and FC simultaneously). CBV measurements using IOS imaging (with 530 nm illumination) were conducted in 11 male mice. CBF measurements using laser Doppler flowmetry were performed in 5 male mice. Tissue oxygenation measurements using spectroscopy (using alternating 470 nm and 530 nm illumination) were conducted in 11 male mice (4 mice were implanted with cannula and electrode). Oxygen measurements with 2PLM were conducted in adult Thy1-GCaMP6f (GP5.11) mice (*n* = 5). Tissue oxygenation measurements using spectroscopy during oxygen challenge (using alternating 470 nm, 530 nm and 660 nm illumination) were conducted in 4 mice (2 male and 2 female, 2 in FL/HL and 2 in FC). Mice were given food and water ad libitum and maintained on 12-h (7:00–19:00) light/dark cycles. All experiments were conducted during the light period of the cycle.

### Surgery

With the exception of mice imaged with 2PLM, all surgeries were performed under isoflurane anesthesia (in oxygen, 5% for induction and 1.5–2% for maintenance). A custom-machined titanium head bolt was attached to the skull with cyanoacrylate glue (#32002, Vibra-tite). The head bolt was positioned along the midline and just posterior to the lambda cranial suture. Two self-tapping 3/32” #000 screws (J.I. Morris) were implanted into the skull contralateral to the measurement sites over the frontal lobe and parietal lobe. A stainless-steel wire (#792800, A-M Systems) was wrapped around the screw implanted in the frontal bone for use as an electrical ground for cortical tissue oxygenation and neural recordings. For CBF measurement using laser Doppler flowmetry (*n* = 5 mice), CBV (*n* = 11 mice) measurement using IOS imaging or brain oxygenation measurement using spectroscopy (*n* = 11 mice), a polished and reinforced thin-skull (PoRTS) window was made covering the right hemisphere^11,25,34,40,71^. For simultaneous measurement of tissue oxygenation and neural activity (*n* = 9 mice), we implanted two electrodes to measure LFP signals differentially. Electrodes were made from Teflon-coated tungsten wire (#795500, A-M Systems) with a ~1 mm length of insulation stripped from the tip. The electrodes were inserted into the cortex to a depth of 800 µm at 45° angle along the rostral/caudal axis using a micromanipulator (MP-285, Sutter Instrument) through two small burr holes made in the skull. The two holes for the electrodes were made ~1–1.5 mm apart to allow insertion of the oxygen probe between the two electrodes in following experiments. The holes were then sealed with cyanoacrylate glue. For spectroscopy imaging experiments with intracortical infusion (*n* = 4 mice), two small craniotomies were made at the edge of the thinned area of skull, and a cannula (dummy cannula: C315DCS; guide cannula: C315GS-4, Plastic One) was inserted into the upper layers of cortex at a 45° angle via one craniotomy. The stereotrode was placed 1.75 ± 0.5 mm away from the cannula through the other craniotomy. The screws, ground wire, electrodes and cannula were connected to the head-bolt via the midline suture using cyanoacrylate glue and black dental acrylic resin (#1530, Lang Dental Manufacturing Co.) to minimize skull movements. For tissue oxygenation (*n* = 37 mice) and laminar electrophysiology (*n* = 7 mice) experiments, the measurement sites were marked with ink and covered with a thin layer of cyanoacrylate glue. For oxygenation measurements using 2PLM, we used mice chronically implanted with a cranial window over FL/HL (*n* = 3 mice) or the olfactory bulb (*n* = 2 mice)^4,18^. Following the surgery, mice were then returned to their home cage for recovery for at least one week, and then started habituation on experimental apparatus.

### Habituation

Animals were gradually acclimated to head-fixation on a spherical treadmill^11,19,39^ or a rotating disk^4,18,26^ with one degree of freedom over at least three habituation sessions. The spherical treadmill was covered with nonabrasive anti-slip tape (McMaster-Carr) and attached to an optical rotary encoder (#E7PD-720-118, US Digital) to monitor locomotion. Mice were acclimated to head-fixation for ~15 min during the first session and were head-fixed for longer durations (>1 h) in the subsequent sessions. Mice were monitored for any signs of stress during habituation. In all cases, the mice exhibited normal behaviors such as exploratory whisking and occasional grooming after being head-fixed. Heart-rate related fluctuations were detectable in the IOS^40^ and varied between 7 and 13 Hz for all mice after habituation, which is comparable to the mean heart rate (~12 Hz) recorded telemetrically from mice in their home cage^72^. For oxygen measurements using 2PLM, a rotating disk treadmill was added to the cage a week prior to the surgery and restraint-habituation sessions started 3-4 days after surgery recovery. For habituation for 2PLM experiments, the animals were place head-fixed below the microscope and free to run on the treadmill. During each habituation session, a thermocouple (same as used for imaging) was placed close to the nostril in order to acclimate the mouse with its presence. Habituation sessions were performed 2–4 times during each day over the course of one week, with the duration increasing from 5 to 45 min.

### Physiological measurements

Data from all experiments were collected using custom software written in LabVIEW (version 2014, National Instruments).

### Behavioral measurements

The treadmill movements were used to quantify the locomotion events of the mouse. The animal was also monitored using a webcam (Microsoft LifeCam Cinema®) as an additional behavioral measurement.

### CBV measurements using IOS imaging

Reflectance images were collected during periods of green LED light illumination at 530 nm (M530L3, Thorlabs). A CCD camera (Dalsa 1M60) was operated at 30 Hz with 4 × 4 binning (256 × 256 pixels). This reflectance change observed with IOS closely tracks measurements of vessel diameters made with two-photon microscopy^34^. The consistency with microscopic measurements of vessel diameter, combined with its very high signal-to-noise ratio^25^, and spatial resolution (less than 200 µm^73^), makes IOS suitable for detecting hemodynamic responses to locomotion. While neurally-evoked dilations initiate in the deeper layers of the cortex, the dilations propagate up the vascular tree to the surface arteries^74^, where they can be easily detectable with IOS. Finally, local changes in CBF are intimately linked with vessel diameter^34^, as dilations of vessels will reduce the resistance of the vascular network, increasing the blood flow through it.

### PtO_2_ measurements using polarographic electrode

On the day of measurement, the mouse was anesthetized with isoflurane (5% for induction and 2% for maintenance) for a short surgical procedure (~20 min). A small (~100 × 100 μm) craniotomy was made over FC (1.0–3.0 mm rostral and 1.0–2.5 mm lateral from bregma) or FL/HL (0.5–1.0 mm caudal and 1.0–2.5 mm lateral from bregma), and dura was carefully removed (Fig. 2a). The craniotomy was then moistened with warm artificial cerebrospinal fluid (aCSF) and porcine gelatin (Vetspon). The mouse was then moved to and head-fixed on the spherical treadmill. Oxygen measurements started at least one hour after the mouse woke up from anesthesia to minimize the effects of anethesia^19^.

Cerebral tissue oxygenation was recorded with a Clark-type oxygen microelectrode (OX-10, Unisense A/S, Aarhus, Denmark). A total of nine probes were used in this study, with an average response time of 0.33 ± 0.11 s (*n* = 9 probes, Supplementary Fig. 3a, b). No compensation for the delay was performed. The oxygen electrodes were calibrated in air-saturated 0.9% sodium chloride (at 37 °C) and oxygen-free standard solution [0.1 M sodium hydroxide (SX0607H-6, Sigma-Aldrich) and 0.1 M sodium ascorbate (A7631, Sigma-Aldrich) in 0.9% sodium chloride] before and after each experiment. The linear drift of the oxygen electrode signal (1.86 ± 1.19% during each hour, Supplementary Fig. 3c, d) was corrected by linearly interpolating between pre- and post-experiment calibrations. The oxygen electrode was connected to a high-impedance picoammeter (OXYMeter, Unisense A/S, Aarhus, Denmark), whose output signals were digitalized at 1000 Hz (PCI-6259, National Instruments). Current recordings were transformed to millimeters of mercury (mmHg) using the calibrations with air-saturated and oxygen-free solutions.

For oxygen polarography measurements, the oxygen microelectrode was positioned perpendicular to the brain surface and advanced into the cortex with a micromanipulator (MP-285, Sutter Instrument). The zero depth was defined as when the tip of the oxygen microelectrode touches the brain surface under visual inspection. The probe was then advanced to depth of 100, 300, 500, and 800 μm below the pia at the rate of 0.2 μm for each step, and 30–40 min data were recorded for each depth. The tissue was allowed to recover for at least 5 min before the start of each recording.

In experiments investigating effects of suppressing vasodilation on cortical tissue oxygenation dynamics (Fig. 3e), a cocktail of ionotropic glutamate receptor antagonists 6-cyano-7-nitroquinoxaline-2,3-dione (CNQX, 0.6 mM), NMDA receptor antagonist (2R)-amino-5-phosphonopentanoic acid (AP5, 2.5 mM) and GABA_A_ receptor agonist muscimol (10 mM) were applied to suppress neural activity. All drugs were applied topically over the craniotomy and were allowed to diffuse into the cortical tissue for at least 90 min before the oxygen measurements. The efficacy of the CNQX/AP5/muscimol cocktail was monitored with simultaneously recorded neural activity. Neural data were amplified 1000x and filtered (0.1–10 kHz, DAM80, World Precision Instruments) and then sampled at 30 kHz (PCI-6259, National Instruments). The oxygen signal in these experiments was recorded at a depth of ~100–200 μm.

At the end of the experiment, the mouse was deeply anesthetized, and a fiduciary mark was made by advancing an electrode (0.005” stainless steel wire, catalog #794800, A-M systems) into the brain with a micro-manipulator to mark the oxygen measurement site.

### Respiration measurements using thermocouple

We conducted simultaneous respiration recordings in a subset of mice (*n* = 28) along with cortical oxygen measurements. Measurements of breathing were taken using 40-guage K-type thermocouples (TC-TT-K-40-36, Omega Engineering) placed near the mouse’s nose (~1 mm), with care taken to not contact the whiskers. Data were amplified 2000×, filtered below 30 Hz (Model 440, Brownlee Precision), and sampled at 1000 Hz (PCI-6259, National Instruments). Downward and upward deflections in respiration recordings correspond to inspiratory and expiratory phases of the respiratory cycle, respectively (Fig. 4a). We identified the time of each expiratory peak in the entire record as the zero-crossing point of the first derivative of the thermocouple signal.

### Laminar electrophysiology

Laminar electrophysiology recordings were performed in a separate set of mice (*n* = 7, Fig. 1e). On the day of measurement, the mouse was anesthetized using isoflurane (in oxygen, 5% for induction and 2% for maintenance). Two small (1 × 1 mm^2^) craniotomies were performed over FC (1.0–2.5 mm rostral and 1.0–2.5 mm lateral from bregma) and FL/HL (0.5–1.0 mm caudal and 1.0–2.5 mm lateral from bregma) over the contralateral hemisphere (Fig. 1e), and the dura was carefully removed. The craniotomies were then moistened with warm saline and porcine gelatin (Vetspon). After this short surgical procedure (~20 min), the mouse was then transferred to the treadmill where it was head-fixed. Measurements started at least one hour after the cessation of anesthesia^19^.

Neural activity signals were recorded using two linear microelectrode arrays (A1x16-3mm-100-703-A16, NeuroNexus Technologies). The electrode array consisted of a single shank with 16 individual electrodes with 100 µm inter-electrode spacing. The signals were digitalized and streamed to SmartBox^TM^ via a SmartLink headstage (NeuroNexus Technologies). The arrays were positioned perpendicular to the cortical surface, one was in FL/HL and the other one was in FC on the contralateral side. Recording depth was taken from the manipulator (MP-285, Sutter Instrument) recordings. The neural signals were filtered (0.1-10k Hz bandpass), sampled at 20 kHz using SmartBox 2.0 software (NeuroNexus Technologies).

### CBF measurements using laser Doppler flowmetry

We measured CBF responses to voluntary locomotion in a separate set of mice (*n* = 5) using laser Doppler flowmetry (OxyLab, Oxford Optronix)^40^. The probe was fixed 0.3 mm above the PoRTS window at a 45° angle. Data were sampled at 1000 Hz (PCI-6259, National Instruments).

### Brain oxygen measurements using spectroscopy

We mapped the spatiotemporal dynamics of oxyhemoglobin and deoxyhemoglobin concentrations using their oxygen-dependent optical absorption spectra^43^. Reflectance images were collected during periods of green LED light illumination at 530 nm (equally absorbed by oxygenated and deoxygenated hemoglobin, M530L3, Thorlabs) or blue LED light illumination at 470 nm (absorbed more by oxygenated than deoxygenated hemoglobin, M470L3, Thorlabs). For these experiments, a CCD camera (Dalsa 1M60) was operated at 60 Hz with 4 × 4 binning (256 × 256 pixels), mounted with a VZM300i optical zoom lens (Edmund Optics). Green and blue reflectance data were converted to changes in oxy- and deoxyhemoglobin concentrations using the modified Beer-Lambert law with Monte Carlo-derived wavelength-dependent path length factors^43^. We used the cerebral oxygenation index^44^ (i.e., HbO-HbR) to quantify the change in oxygenation, as calculating the percentage change requires knowledge of the concentration of hemoglobin on a pixel-by-pixel basis, which is not feasible given the wide heterogeneity in the density of the cortical vasculature^41^.

In a subset of mice (*n* = 4), intracortical drug infusion were conducted via a cannula. Mice were placed in the imaging setup, and we then acquired 40 min of imaging, neural and behavioral data with the dummy cannula in place. The dummy cannula was then slowly removed and replaced with an infusion cannula. The interface between the infusion cannula and the guide cannula was sealed with Kwik-Cast (World Precision Instruments). A cocktail of CNQX (0.6 mM)/AP5 (2.5 mM)/muscimol (10 mM), or L-NAME (100 µM), or aCSF was locally infused at a rate of 25 nL min^−1^ for a total volume of 500 nL. Drugs and vehicle controls were infused in a counterbalanced order. The efficacy of the CNQX/AP5/muscimol cocktail was monitored with simultaneously recorded neural activity using two tungsten electrodes. Neural data were amplified 1000× and digitally filtered (0.1–10 kHz, DAM80, World Precision Instruments) and then sampled at 30 kHz (PCI-6259, National Instruments). To verify that the dynamics observed after drug infusion were not due to changes of peripheral cardiovascular system^75,76^, we also injected water, atenolol (2 mg kg^−1^ body weight) and glycopyrrolate (0.5 mg kg^−1^ body weight)^40^ intraperitoneal in the same mouse, and the hemodynamic response was measured described as above (Supplementary Fig. 5).

### Oxygen challenge experiments

The mouse was head-fixed on a spherical treadmill, and a nose cone was fixed ~2 cm in front of the nose, with care taken to not contact the whiskers. Two gases were administered during a 5-min spectroscopy trial in the following order: 1 min compressed air (21% O_2_), 3 min 100% oxygen, and then 1 min compressed air. The flow rate of both gas mixtures was regulated by a flowmeter to be 1 L min^−1^. Mice were breathing compressed air (1 L min^−1^) for at least 2 min between trials, to ensure physiological parameters return to baseline. Reflectance images were collected during periods of green LED light illumination at 530 nm (equally absorbed by oxygenated and deoxygenated hemoglobin, M530L3, Thorlabs) or blue LED light illumination at 470 nm (absorbed more by oxygenated than deoxygenated hemoglobin, M470L3, Thorlabs) or red LED light illumination at 660 nm (absorbed more by deoxygenated than oxygenated hemoglobin, M660L2, Thorlabs).

### Brain oxygen measurements using 2PLM

A complete description of 2PLM can be found in previous reports^4,8,18^. In brief, the oxygen sensor Oxyphor 2P^45^ was injected intravenously (final plasma concentration of 5 µM) under a brief isoflurane anesthesia (4%, <3 min). The animals were allowed to recover for at least 1.5 h and then placed below the objective of a custom-built microscope. An acousto-optic modulator (AOM) was placed on the light path from Ti:Sapphire laser (Mira, Coherent; pulse width 250 fs, 76 MHz) to gate the 970 nm light excitation beam. Light was focused onto the center of pial arteries with a water-immersion objective (Olympus LUMFLN 60XW, NA 1.1) and collected emission was forwarded to a red-sensitive photomultiplier tube (PMT, R10699, Hamamatsu) after passing through a dichroic mirror (FF560-Di01, SEMROCK) and a band-pass filter (FF01-794/160, SEMROCK). PMT signals were amplified and sampled at 1.25 MHz by an acquisition card. PaO_2_ was estimated from the signal acquired during the AOM off-phase^8^, after discarding the first 5.6 µs following the end of the AOM on-phase. 200000 decays (50 s) were collected for each acquisition and 3000 decays were used for each lifetime measurement of PaO_2_. During the whole imaging session, respiration and locomotion were constantly monitored with the nasal thermocouple and a velocity encoder connected to the running wheel^26^.

### Depth specificity of different physiological measurements

Note that our measures have different depth specificity. Visible light will primarily assay the upper few hundred micrometers of cortex, while laser Doppler uses infrared light which should sample CBF flow through the upper millimeter or so of cortex. The entire depth of cortex was sampled with oxygen-sensitive and neural activity electrodes. However, we found that tissue oxygen dynamics were relatively homogenous across layers. This is consistent with previous work showing the dilation signal initiates in the parenchyma causing a nearly instantaneous, electrically conducted dilation of the arteriolar tree^26^, so that the dilations of surface vessels in general reflect the dilation dynamics within the brain. Compared to polarographic oxygen electrodes, the spectroscopic imaging samples from arteries, capillaries, and veins. As the veins will be deoxygenated by the increased metabolic rate during periods of sustained neural activity, this will tend to reduce the measured oxygen change in the spectroscopic studies as compared to the polarography measurements, which primarily report tissue oxygen concentrations.

### Drugs

All drugs were purchased from Sigma-Aldrich except aCSF (#3525, Tocris) and sterile water (USP). Muscimol (M1523, 10 mM), CNQX (C127, 0.6 mM), AP5 (A5282, 2.5 mM) and L-NAME (N5751, 100 µM) were diluted in aCSF. Atenolol (A7655, 2 mg kg^−1^ body weight) and glycopyrrolate (SML0029, 0.5 mg kg^−1^ body weight) were diluted in sterile water. All drug solution was stored at −20 °C and warmed up using a water bath (WB05A12E, PolyScience) immediately before application. Oxyphor 2P was kindly provided by Sergei Vinogradov.

### Histology

At the conclusion of the experiment, mice were deeply anesthetized with 5% isoflurane, transcardially perfused with heparinized saline, and then fixed with 4% paraformaldehyde. The brains were extracted and sunk in a 4% paraformaldehyde with 30% sucrose solution. The flattened cortices were sectioned tangentially (60 µm thick sections) using a freezing microtome and stained for the presence of cytochrome-oxidase^25,42^. The anatomical locations of the oxygen measurement sites were then reconstructed using a combination of vascular images taken during surgery and the stained brain slices using Adobe Illustrator CS6 (Adobe Systems).

### Data analysis

All data analyses were performed in Matlab (R2015b, MathWorks) using custom code (by Q.Z., K.W.G., and P.J.D.).

### Locomotion events identification

Locomotion events^11,25,39^ from the spherical treadmill were identified by first applying a low-pass filter (10 Hz, 5th order Butterworth) to the velocity signal from the optical rotary encoder, and then comparing the absolute value of acceleration (first derivative of the velocity signal) to a threshold of 3 cm s^−2^. Periods of locomotion were categorized based on the binarized detection of the treadmill acceleration:

1 $$\delta \left( t \right) = H\left( {\left| {a_t} \right| - a_c} \right) = \left\{ {\begin{array}{*{20}{c}} {1,\;\left| {a_t} \right| \ge a_c} \\ {0,\;\left| {a_t} \right| \;< \;a_c} \end{array}} \right.$$

where *a*_*t*_ is the acceleration at time *t*, and *a*_*c*_ is the treadmill acceleration threshold.

### Spontaneous and evoked activity

To characterize spontaneous (non-locomotion-evoked) activity, we defined resting periods as periods started 4 s after the end of previous locomotion event and lasting more than 10 s. Locomotion-evoked events were defined as segments with at least 3 s of resting prior to the onset of locomotion and followed by at least 5 s of locomotion. For oxygen measurements using polarographic electrode and 2PLM, the locomotion segments need to be at least 10 s in duration.

### Oxygen data preprocessing

Oxygen data from polarographic electrodes were first low-pass filtered (1 Hz, 5th order Butterworth). The oxygen data were then down-sampled to 30 Hz to align with binarized locomotion events for calculation of locomotion-triggered average and HRF.

### Laminar neural activity

The neural signal was first digital filtered to obtain the local field potential (LFP, 0.1–300 Hz, 5th order Butterworth) and multiunit activity (MUA, 300-3000 Hz, 5th order Butterworth)^11,25^. Time-frequency analysis of LFP signal was conducted using multi-taper techniques (Chronux toolbox version 2.11, http://chronux.org/)^77^. The power spectrum was estimated on a 1 s window with ~1 Hz bandwidth averaged over nine tapers. MUA signals were low-pass filtered (5 Hz, Bessel filter). The locomotion-evoked LFP power spectrum was converted into relative power spectrum by normalizing to the 3 s resting period prior to the onset of locomotion. Spike rate was obtained by counting the number of events that exceed an amplitude threshold (three SDs above background) in each 1 millisecond bin.

### Spike sorting

Sortable spike waveforms were extracted from MUA recordings using spike times identified from threshold crossings at four SDs of the mean. Spike waveforms were interpolated using a cubic spline function (MATLAB function: interp1) and were normalized by the amplitude of the peak of the action potential. We classified waveforms as fast spiking (FS) or regular spiking (RS) neurons based on the peak-to-trough-duration of the normalized waveform of each spike. Peak-to-trough times of all spikes across all layers were binned at 0.05 ms intervals (the minimal temporal resolution at 20 kHz sampling rate). A histogram of peak-to-trough times was fitted as a sum of two Gaussian distributions (Supplementary Fig. 2a, f), and a receiver operator characteristic curve was used to segregate spikes in a given bin as either FS or RS waveforms using a 95% probability of belonging to a group as the inclusion threshold. Spikes not reaching the inclusion threshold for either group were not included in the analysis. Fast spiking waveforms (Supplementary Fig. 2b, g) were characterized by short durations between action potential peak and peak of hyperpolarization, peak-to-trough-duration as described previously^32^. We characterized the RS and FS activity across different cortical layers during both resting and locomotion periods. To directly compare locomotion-related changes between FS and RS neurons, we calculated the percentage change of FS (∆FS) and RS (∆RS) spike rates (Supplementary Fig. 2c–e, h–j), which normalizes for absolute rate differences.

### HRF and NRF

We considered the neurovascular relationship to be a linear, time-invariant system^27^. To provide a model-free approach to assess the relationship between laminar tissue oxygenation and laminar neural activity, the HRF and NRF were calculated by deconvoluting tissue oxygenation signal or neural activity signal to locomotion events, respectively, using the following equation:

2 $$H_{\left( {k + 1} \right) \times 1} = \left( {L^TL} \right)^{ - 1}L^TV_{\left( {m + k} \right) \times 1}$$

*H* is the HRF or NRF, *V* is the tissue oxygenation signal or neural activity signal. L is a Toeplitz matrix of size (m + k) × (k + 1) containing measurements of locomotion events (*n*):

3 $$L\left( {\mathop{{\mathrm{n}}}\limits^{\rightharpoonup}} \right) = \left( {\begin{array}{*{20}{c}} 1 & {n_1} & 0 & 0 & \cdots & 0 \\ 1 & {n_2} & {n_1} & 0 & \cdots & 0 \\ \vdots & \vdots & {n_2} & {n_1} & \cdots & \vdots \\ \vdots & {n_k} & \vdots & {n_2} & \cdots & {n_1} \\ \vdots & 0 & {n_k} & \vdots & \cdots & {n_2} \\ \vdots & \vdots & \vdots & {n_k} & \ddots & \vdots \\ 1 & 0 & 0 & 0 & \cdots & {n_k} \end{array}} \right)$$

### Cross-correlation analysis

Cross-correlation analysis was performed between simultaneously recorded neural/respiration and oxygen signals to quantify the relationship between fluctuations. For spontaneous correlations, only periods of rest lasting more than 30 s were used, with a four-second buffer at the end any locomotion event. One mouse was excluded from resting correlation analysis as there were no resting segments long enough to meet the selection criteria. We also calculated the correlations using all the data including periods with locomotion. To check the spatiotemporal distribution of the correlation, we calculated cross-correlogram between PtO_2_ and LFP power in each frequency band (Supplementary Fig. 7). Briefly, LFP signals were separated into frequency bands (~1 Hz resolution with a range of 0.1–150 Hz) by calculating the spectrogram (mtspecgramc, Chronux toolbox)^77^, and then we calculated the temporal cross-correlation between power in each frequency band and the oxygen concentration (xcorr, MATLAB). Positive delays denote the neural signal lagging the oxygen signal. The oxygen tension and neural activity were both low-pass filtered below 1 Hz before calculating the cross-correlation. The temporal cross-correlation between respiratory rate and oxygen signals was also calculated over a similar interval (xcorr, MATLAB). Statistical significance of the correlation was computed using bootstrap resampling from 1000 reshuffled trials.

### Arterial oxygenation changes during the respiration cycle

To evaluate the arterial oxygen tension change within the respiration cycle, we selected oxygen measurements during periods with regular respiratory rate (average frequency 2.5 Hz, SD ≤ 0.6 Hz, selected based on the criterion that the maximum respiration frequency was 5 Hz). For each recording, the phosphorescent decays were aligned according to their place in the phase of the respiratory cycle before being pooled into 20 ms bins, and PaO_2_ was then calculated for each of the bins, which gives PaO_2_ over the respiratory cycle for a recording (Fig. 4g). The average PaO_2_ over the respiratory cycle was then calculated by averaging PaO_2_ over the respiratory cycle across multiple trials (Fig. 4h, top). A curve was fitted to the PaO_2_ data over the respiration cycle using a first-order binomial filter (five repetitions). To quantify the changes of PaO_2_ over one respiration cycle, we defined a 40 ms time period when PaO_2_ reaches minimum (*T*_min_) and maximum (*T*_max_), respectively (Fig. 4h, top). To further determine whether the fluctuations of oxygen tension was induced by respiration, we performed a phase randomization test: we calculated the power spectrum of arterial oxygen tension, and determined the peak frequency in the power spectrum, and checked that it was within the respiration frequency range (Fig. 4h, bottom). Statistical significance of this peak was calculated by reshuffling the arterial oxygen measurements, and the 95% confidence interval was calculated using 10,000 reshuffled trials.

### Ordinary coherence and partial coherence

We used coherence analysis to reveal correlated oscillations and deduce functional coupling among different signals. The ordinary coherence between two signals *x* and *y* are defined as

4 $$C_{xy}^2\left( f \right) = \frac{{S_{xy}^2\left( f \right)}}{{S_x\left( f \right)S_y\left( f \right)}},$$

where *S*_*x*_ (*f*) and *S*_*y*_ (*f*) are the auto-spectra of the signals, and *S*_*xy*_ (*f*) is the cross-spectrum. For ordinary coherence analysis between two signals (*x* and *y*), highly coherent oscillations can occur if they are functionally connected or because they share a common input. To differentiate between these possibilities, we also computed the partial coherence, i.e., coherence between two signals (*x* and *y*) after the removal of the components from each signal that are predictable from the third signal (*z*). The partial coherence function measuring the relationship of *x* and *y* at frequency *f* after removal of *z* is defined as

5 $$PC_{xy - z}^2 = \frac{{S_{xy - z}S_{yx - z}}}{{S_{xx - z}S_{yy - z}}}$$

where *S*_*xx−z*_ and *S*_*yy-z*_ is the auto-spectra associated with the residual part of *x* and *y* after removing the part coherent with *z*, respectively. *S*_*xy−z*_ is the cross-spectrum between the residual part of *x* and *y* after removing the part coherent with *z*. If all the networks are connected, partial coherence will be between zero and the level of the ordinary coherence. If the connection behaves in an asymmetric manner, i.e., signal *z* affects *x* and *y* differentially, the coherence between two signals may increase after partialization (Supplementary Fig. 9a).

### Statistical analysis

Statistical analysis was performed using Matlab (R2015b, Mathworks). All summary data were reported as the mean ± SD unless stated otherwise. Normality of the samples were tested before statistical testing using Anderson-Darling test (adtest). For comparison of multiple populations, the assumption of equal variance for parametric statistical method was also tested (vartest2 and vartestn). If criteria of normality and equal variance were not met, parametric tests (*t* test, one-way ANOVA) were replaced with a nonparametric method (Mann-Whiteney *U*-test, Wilcoxon signed-rank test, Kruskal-Wallis ANOVA). All *p* values were Bonferroni corrected for multiple comparisons. Significance was accepted at *p* < 0.05.

### Computational modeling

We used two-dimensional, axi-symmetric, time-dependent finite element models to simulate the coupled physics of blood flow and oxygen diffusion/consumption. All the simulations were performed in COMSOL (COMSOL Inc.) using the weak form partial differential equation module. The model was divided into two domains: the fluid domain and the solid domain. The fluid domain, which represents the arterial lumen, was assumed to be a cylinder of radius R_1_ and a length of L_a_. The solid domain, which represents the surrounding tissue that is oxygenated by the arteriole, was assumed to be a cylinder of radius R_2_ and of the same length L_a_. To account for the dilation of the blood vessel, the equations of transport in the fluid domain were written in an arbitrary Lagrangian-Eulerian formulation. Therefore, the computational mesh inside the fluid domain can move arbitrarily to optimize the shapes of the elements. The equations in the solid domain were implemented in their Lagrangian formulation, which is more appropriate for simulating solid mechanics. We presented all the equations in their more familiar physical (Eulerian) coordinates for simplicity.

In the fluid domain, the blood flow was modeled by Stokes law (Eq. 6) to simulate the low Reynolds' number flow^78^ in the arterioles.

6 $$\nabla {p} = {\boldsymbol{\mu }}\nabla ^2{\boldsymbol{v}},\nabla \cdot {\boldsymbol{v}} = 0$$

where ***v*** is the fluid velocity, *p* is the fluid pressure and *μ* is the dynamic viscosity of blood.

The oxygen transport in blood was modeled by the advection-diffusion equation (Eq. 7), and subject to the equilibrium of free and hemoglobin-bound oxygen.

7 $$\frac{{\partial c_T}}{{\partial t}} = - {\boldsymbol{v}} \cdot \nabla c_T + \nabla \cdot \left[ {D_{\mathrm{O}_2}\nabla c_F} \right]$$

In Eq. 7, *c*_*T*_ is the total concentration of oxygen in blood and can be represented by the sum of concentration of free (*c*_*F*_) and hemoglobin-bound (*c*_*B*_) oxygen; and $D_{\mathrm{O}_2}$ is the diffusion coefficient of oxygen. The equilibrium relation between free and bound oxygen (Eq. 8) is given by the oxygen saturation-dissociation curve^79^ ($S_{\mathrm{O}_2}$, a function of free oxygen) and the hemoglobin concentration *C*_Hb_.

8 $$c_B = 4C_{\mathrm{Hb}}S_{\mathrm{O}_2}\left( {c_F} \right)$$

The mesh movement in the dilating arteriole was modeled using a linear model (Eq. 9) since the dilation occurs in only one direction (radially).

9 $$u_{\mathrm{mr}} = \frac{r}{{R_1}}\mathrm{dilation}\left( t \right)\,\mathrm{and}\,u_{\mathrm{mz}} = 0$$

where, *u*_mr_ and *u*_mz_ are the radial and axial components of the mesh displacement, respectively.

In the solid domain, we calculate the tissue displacement, ***u***_*s*_, and the concentration of free oxygen *c*_*F*_. The transport of oxygen in the tissue was modeled by Fick's law of diffusion (Eq. 10).

10 $$\frac{{\mathrm{d}c_F}}{{\mathrm{d}t}} = \nabla \cdot \left[ {D_{O_2}\nabla c_F} \right] - {\mathrm{CMRO}}_2$$

where, $\frac{\mathrm{d}}{{\mathrm{d}t}}$ is the material time derivative of *c*_*F*_, which is different from the partial time derivative, $\frac{\partial }{{\partial t}}$, seen in Eq. 7. The time-profile of CMRO_2_ used in the model was given in Fig. 5c.

The deformation of brain tissue was modeled by a nearly incompressible (Poisson’s ratio = 0.45) linear elastic model. Though the volume of the brain tissue in the model will decrease slightly, we kept the integrated CMRO_2_ of the tissue unchanged, so these deformations will not change CMRO_2_. We calculated the displacement of the tissue (***u***_*s*_) based on the equations for linear elasticity (Eq. 11).

11 $$\nabla \cdot {{\boldsymbol{\sigma }}_s} = {\mathbf{0}}$$

where, ***σ***_S_ is the Cauchy stress, and is given by the elastic coefficients *μ*_*s*_ and *λ*_*s*_ and the linearized elastic strain ***ϵ***_s_ using Eqs. 12 and 13.

12 $${\boldsymbol{\sigma }}_s = 2{\mu}_s {\boldsymbol{\epsilon}}_s + {\lambda}_s Tr \left[ {\boldsymbol{\epsilon}}_s \right]{\boldsymbol{I}}$$

13 $${\boldsymbol{\epsilon}} _s = \frac{1}{2} \left(\nabla {\boldsymbol{u}}_s + \nabla {\boldsymbol{u}}_s^T \right)$$

All the calculations were performed for an initial stationary (steady-state) case and the solution was used as the initial condition for the dynamic simulation that followed. ***I*** is the identity tensor. *Tr* is the trace.

We modeled a cylinder with length L_a_ of 100 μm. to simulate measurements in layer I. As arterial dilation can cause a decrease in the flow resistance and result in an increase in blood flow velocity, we dictated the fluid flow by a pressure difference across the two ends of the arteriole. This was achieved by applying a traction equal to p_1_ mmHg at the inlet (Fig. 5a, top) of the arteriole and zero traction at the outlet (Fig. 5a, bottom). The pressure difference p_1_ was chosen so that the blood velocity was approximately 2 mm s^−1^ ^80^. The concentration of free oxygen (and consequently the hemoglobin-bound oxygen) was fixed at the inlet of the arteriole. A no-flux boundary condition was used at the three open ends (*z* = 0; *z* = L_a_ and *r* = R_2_) of the tissue for symmetry. The displacement at the tissue-artery interface followed the same time course as the arterial dilation (Eq. 9).

In this model, we used a value of the diffusion coefficient for O_2_ of 2800 µm^2^ s^−1^. Resting arterial oxygen tension in the penetrating vessels was taken to be 35 mmHg. The arterial and tissue radius was assumed to be 9 and 50 µm, respectively. The oxygen consumption rate was uniform outside the arteriole, with a resting CMRO_2_ of 3 µmole cm^−3^ min^−1^. The model was initialized at steady state. We assumed that locomotion induced a 15% increase in CMRO_2_ in FL/HL and a 4% increase in FC (proportionally scaled based on our neural recordings in Fig. 1f–i). We took the optical reflectance changes in FL/HL and FC to be 10% dilation and 5% constriction in vessel diameter, respectively, based on the measured relationship between reflectance and arteriole diameter in our previous study^34^. As ~75% of neural tissue oxygen consumption is activity dependent^47^, we simulated effects of CNQX/AP5/muscimol application by reducing the neuronally dependent portion of CMRO_2_ by 82%, yielding a CMRO_2_ of 1.2 µmole cm^−3^ min^−1^. Details of the model parameters are shown in Supplementary Table 1.

### Reporting summary

Further information on research design is available in the Nature Research Reporting Summary linked to this article.

## Supplementary information

Supplementary Information

Peer Review

Reporting Summary

## Data Availability

The datasets generated and analyzed during the current study are available at https://psu.app.box.com/v/Zhang-O2-during-behavior.

## Code availability

The code used to generate the figures in this paper is available at https://psu.app.box.com/v/Zhang-O2-during-behavior.
