# Supplementary material for: Cerebral oxygenation during locomotion is modulated by respiration
Source: Nat Commun. 2019 Dec 4;10:5515. doi: 10.1038/s41467-019-13523-5 (PMC6893036; doi:10.1038/s41467-019-13523-5)
Supplement: Supplementary file 1 — Supplementary Information [file 41467_2019_13523_MOESM1_ESM.pdf]

Cerebral oxygenation during locomotion is modulated by respiration

Zhang et al.

Supplementary Figures

Supplementary Fig. 1

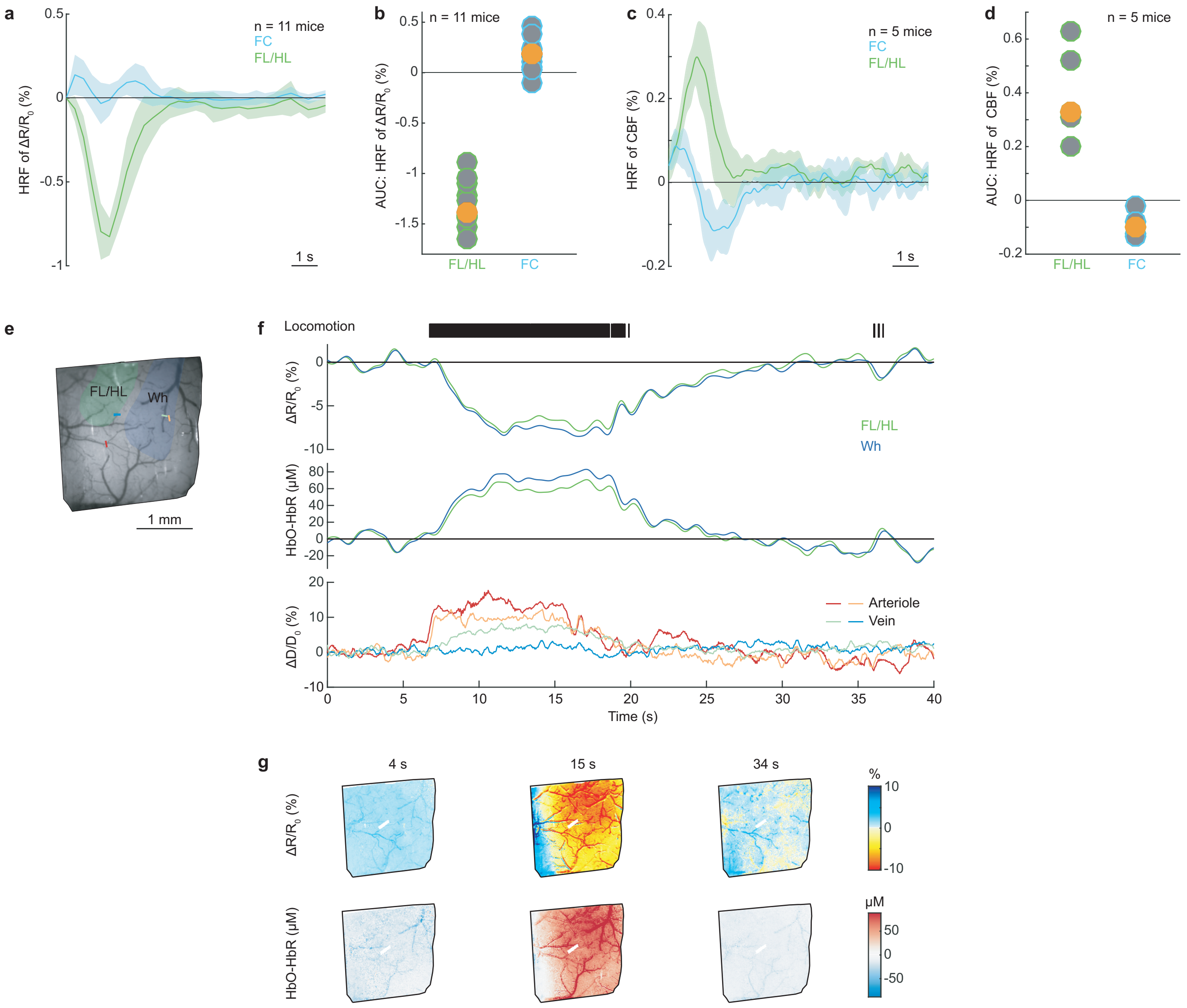

Cerebral blood flow (CBF) and volume (CBV) hemodynamic response functions.

Related to **Fig.1**a-d. (**a**) Hemodynamic response function (HRF) of reflectance change ($\Delta R/R_0$) in FL/HL (green) and FC (blue). (**b**) Integrated area under the curve (AUC) for HRF of ($\Delta R/R_0$) from 0 to 3 seconds. Each circle represents the HRF from a single mouse. The orange circle represents population median. AUC of the CBV HRF was less than zero in FL/HL (Wilcoxon signed-rank test, $p < 0.0001$) and greater than zero in FC (Wilcoxon signed-rank test, $p = 0.002$), indicating a dilation and constriction, respectively. (**c**) As in (**a**) but for cerebral blood flow (CBF). (**d**) As in (**b**) but for cerebral blood flow. AUC of HRF is greater than zero in FL/HL (Wilcoxon signed-rank test, $p = 0.03$) and less than zero in FC (Wilcoxon signed-rank test, $p = 0.03$), indicating increased and decreased flow, respectively. (**e**) Example figure showing a thin-skull window over the somatosensory cortex

under 530 nm illumination. (**f**) Simultaneously measured optical intensity change ($\Delta R/R_0$), HbO-HbR and vessel diameter change ($\Delta D/D_0$) during locomotion. Vessel diameters were calculated from the normalized full width at half minimum of the image intensity along a line perpendicular to the vessel at each point shown in (**e**). (**g**) Images of the $\Delta R/R_0$ (top) and HbO-HbR (bottom) during locomotion. Data in (**a**) and (**c**) are shown as mean ± SD.

Supplementary Fig. 2

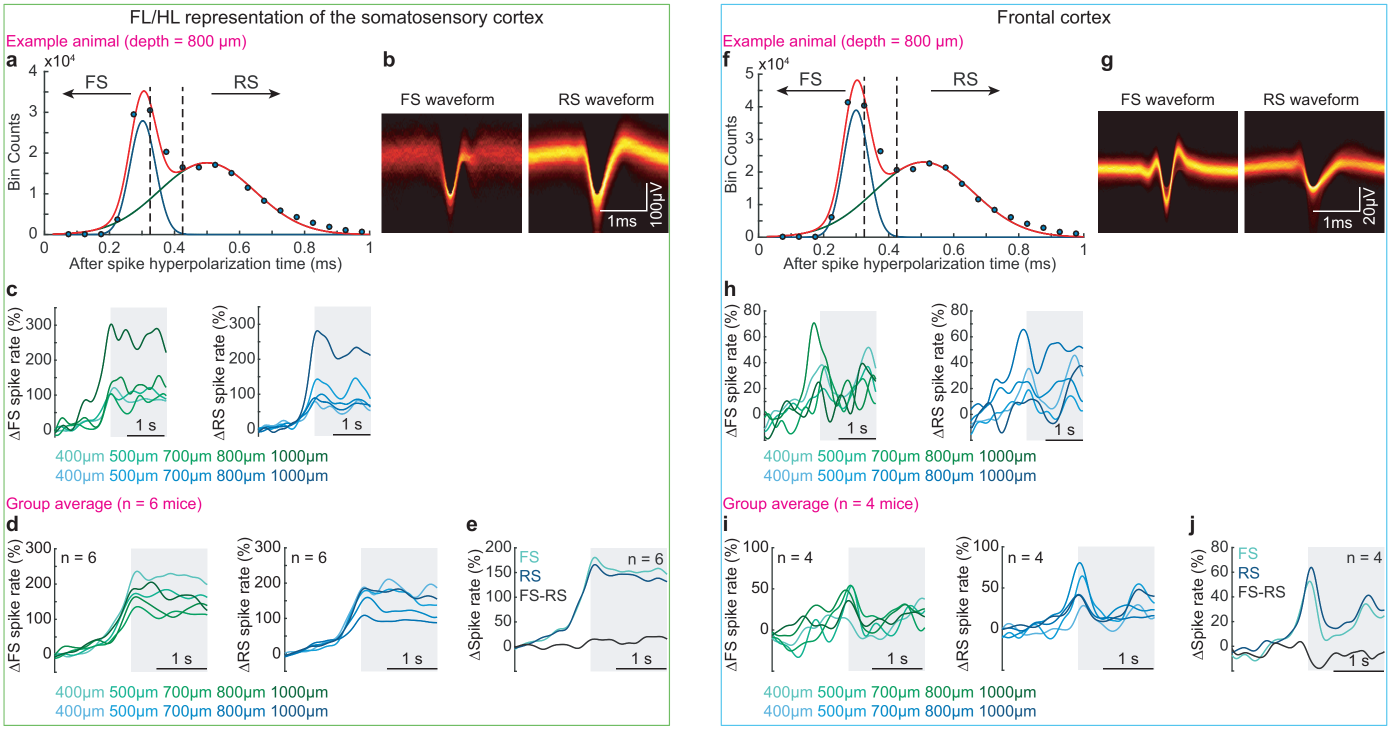

Classification of regular-spiking and fast-spiking neurons based on action potential waveforms and their rate modulations during locomotion in both frontal and somatosensory cortices.

Related to **Fig.1**e-i. We performed spike sorting for neural activity signals acquired using laminar electrodes in both the forelimb/hindlimb representation of the somatosensory cortex (FL/HL, **a-e**) and the frontal cortex (FC, **f-j**). The results shown in (**a**)-(**c**) and (**f**)-(**h**) were from the same mouse shown in **Fig.1**f, g. (**a**) and (**f**) Histogram of action potential (AP) peak-to-trough durations. (**b**) and (**g**) The waveforms of regular spiking (RS) and fast spiking (FS) neurons for an example animal. (**c**) and (**h**) Locomotion-evoked spike rate changes for fast spiking neurons (ΔFS, left) and regular spiking neurons (ΔRS, right) across different cortical layers for an example animal. Gray shaded area denotes the locomotion period. (**d**) and (**i**) Group average of locomotion-evoked spike rate changes for fast spiking neurons (ΔFS, left) and regular spiking neurons (ΔRS, right) across different cortical layers. Gray shaded area denotes locomotion. (**e**) and (**j**) Group average of locomotion-evoked spike rate changes for FS and RS neurons, as well as the difference between changes of FS and RS spike rates. Gray shaded area denotes locomotion.

Supplementary Fig. 3

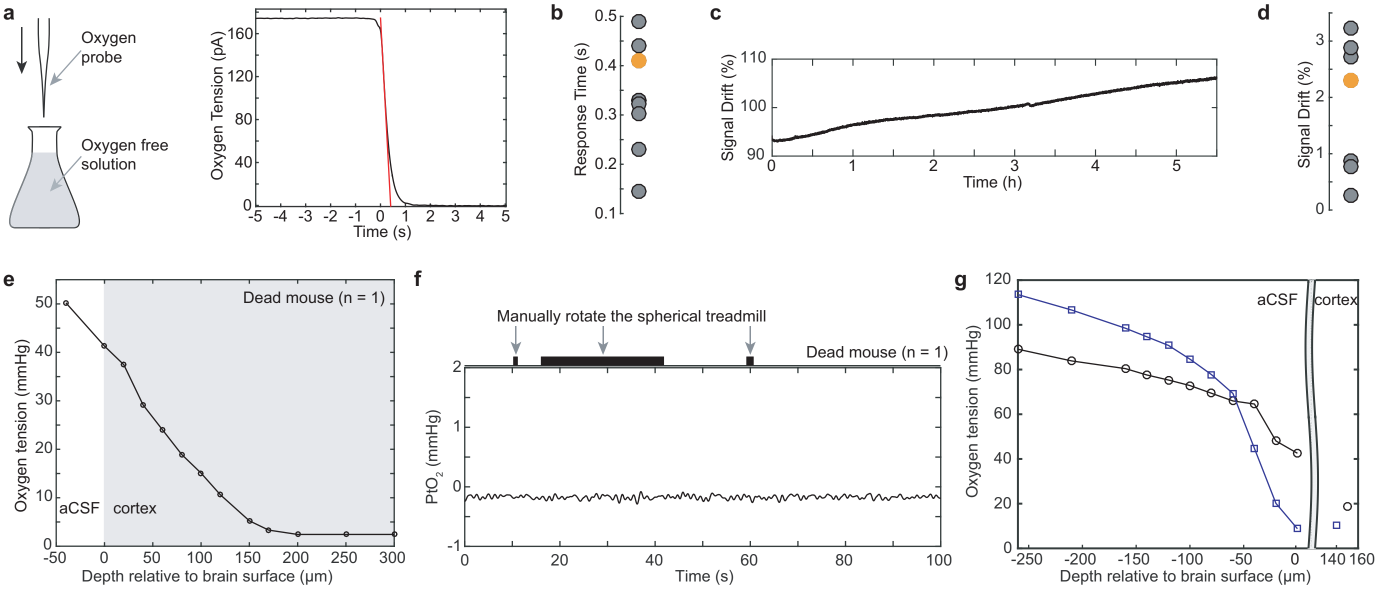

Calibration and properties of oxygen-sensitive electrodes.

Related to **Fig.2** and **Fig.3**. (**a**) Example trace showing the oxygen electrode signal in response to a step change in oxygen concentration. The oxygen electrode response curve was measured by rapidly immersing the electrode into oxygen-free solution (0.1 M sodium hydrochloride and 0.1 M sodium ascorbate solution). The response time of oxygen electrodes was calculated from this curve. (**b**) Average response time for all electrodes used in this study (n = 9). The orange circle indicates the response time of the probe showing in (**a**). (**c**) Example trace showing the oxygen signal change over a 5-hour time period. The stability of the oxygen electrode was tested by quantifying the signal drift while the electrode was immersed in room temperature water for at least 3 hours. (**d**) Average signal drift for a subset of the electrodes (n = 7, 1.86 ± 1.19% during each hour) used in this study. The orange circle indicates the probe showing in (**c**). (**e**) Diffusion of oxygen from the air into the cortex of a dead mouse. To verify the observed oxygen response to locomotion was driven by local perfusion, we also measured oxygen responses in a dead mouse. A similar surgical procedure was applied as described before, and mouse was sacrificed by lung puncture using a 27-gauge needle under deep anesthesia. Oxygen measurements started ~1 hour after the procedure. The oxygen level in the superficial cortex layers of the dead mouse brain were elevated by the oxygen dissolved in the aCSF bathing the craniotomy. (**f**) An example trace showing the oxygen response in the dead mouse brain to ball rotation at 300 μm below the pia. No noticeable changes of PtO_2 were observed during manual ball rotation, showing oxygen signal are not due to electrical noise generated by movement. (**g**) Oxygen levels in the aCSF bathing the craniotomy plotted as a function of distance from the pia in two awake mice during rest. Note that oxygen levels drop near the brain due to its metabolic activity.

Supplementary Fig. 4

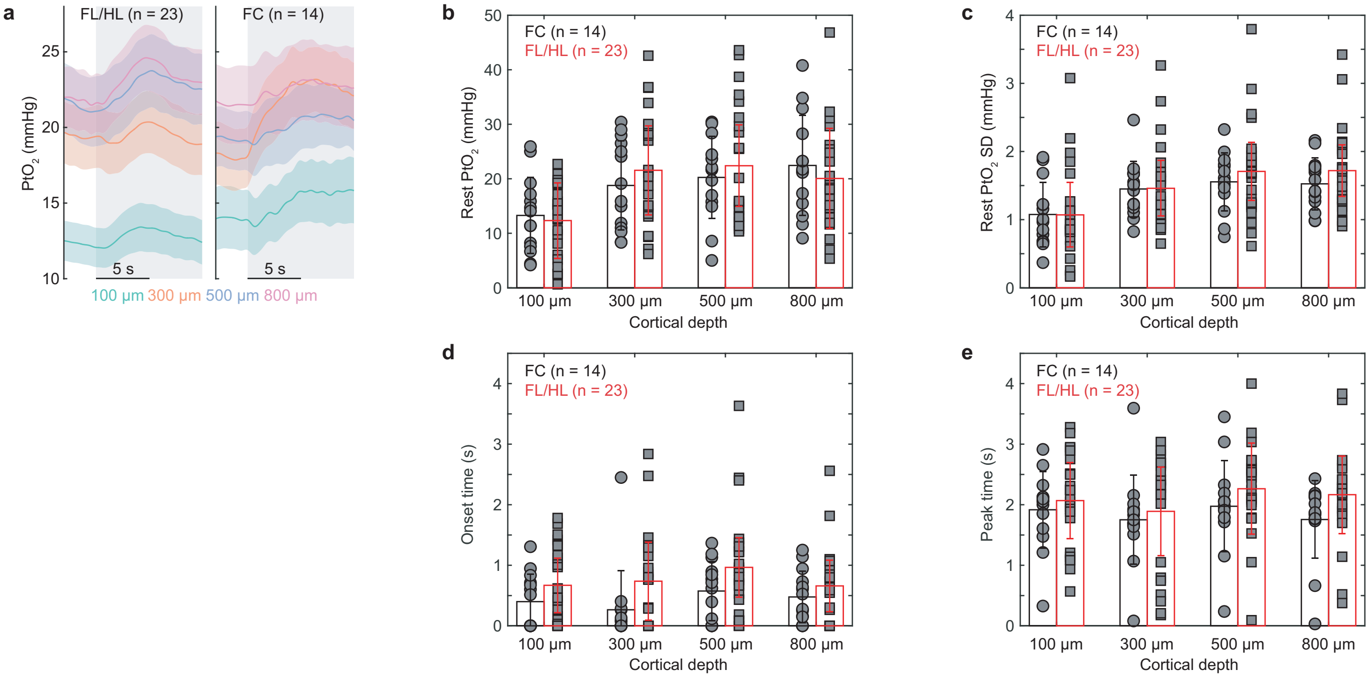

Depth dependence of tissue oxygenation.

Related to **Fig.2**a-d. (**a**) Locomotion-evoked oxygen increases at all measured depths in both FL/HL (left, n = 23 mice) and FC (right, n = 14 mice). Gray shaded area indicates locomotion. (**b**) PtO_2 varies across cortical depth at rest in both FL/HL (n = 23 mice, squares) and FC (n = 14 mice, circles). Each square/circle represents data from a single mouse. Resting PtO_2 was lower at 100 μm compared to 300 μm, 500 μm and 800 μm below the pia (two-way ANOVA, $F(3,147) = 8.1561$, $p < 0.0001$, MATLAB function anovan). Resting PtO_2 were similar at each cortical depth between FL/HL and FC (two-way ANOVA, $F(1,147) = 0.0697$, $p = 0.7921$). (**c**) As in (**b**) but for SD of resting PtO_2. SD was smaller at 100 μm compared to 300 μm, 500 μm and 800 μm below the pia (two-way ANOVA, $F(3,147) = 7.8521$, $p < 0.0001$). Resting fluctuations of PtO_2 were similar at each cortical depth between FL/HL and FC (two-way ANOVA, $F(1,147) = 0.7727$, $p = 0.3808$). (**d**) Onset time of the oxygen HRF. We calculated SD of the HRF during baseline period, and onset time was defined as the time where HRF was more than 1 SD above the baseline mean. The onset time in FC was smaller compared to FL/HL (two-way ANOVA, $F(1,147) = 8.4929$, $p = 0.0041$). No difference were detected across cortical depths (two-way ANOVA, $F(3,147) = 1.2756$, $p = 0.2851$). (**e**) Peak time of the oxygen HRF. The peak time in FC was not significantly different from the FL/HL (two-way ANOVA, $F(1,147) = 3.6$, $p = 0.0598$). No difference were detected across cortical depths (two-way ANOVA, $F(3,147) = 1.05$, $p = 0.3739$). Data are shown as mean ± SEM in (**a**) and ± SD in (**b**), (**c**), (**d**) and (**e**).

Supplementary Fig. 5

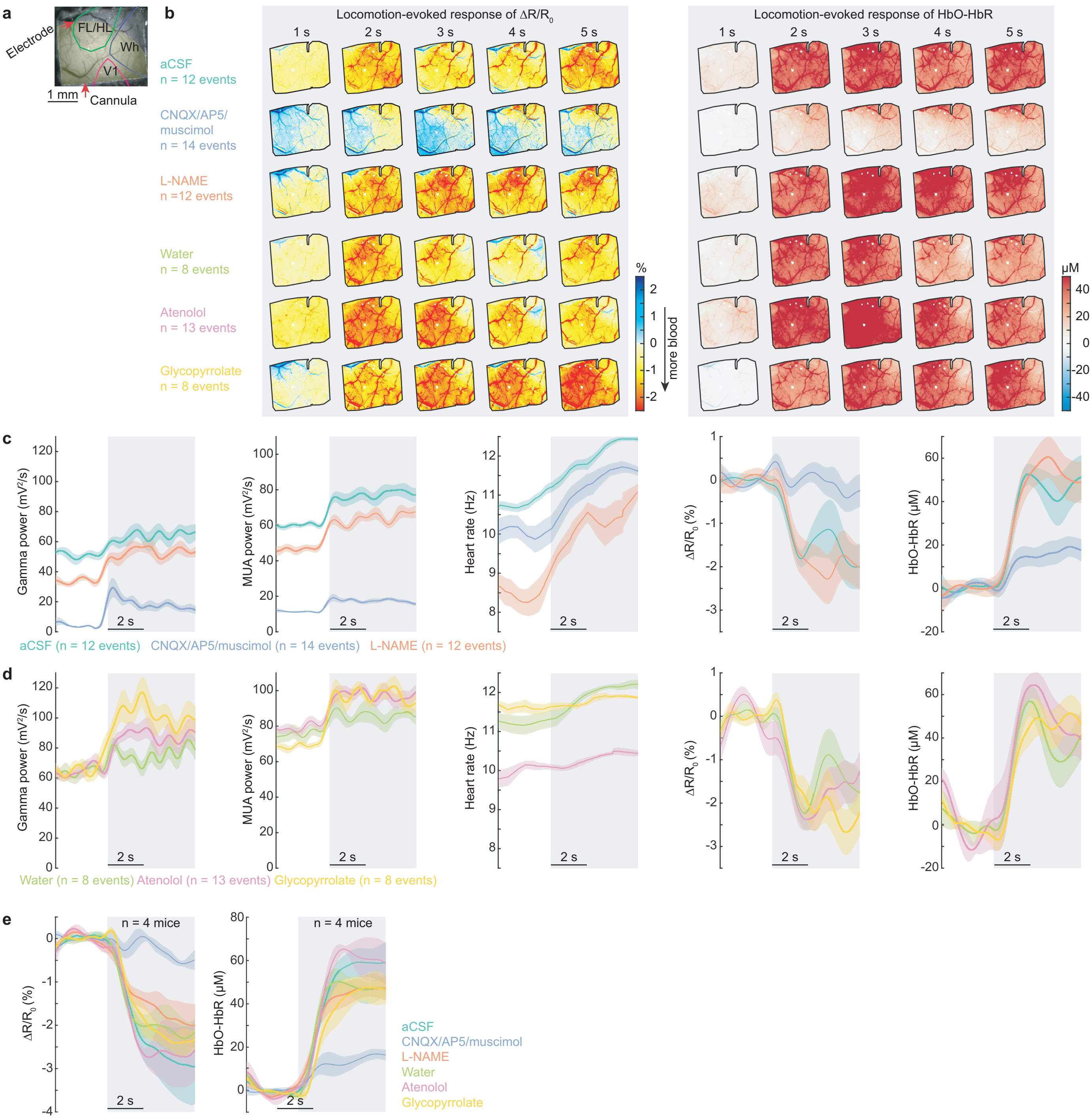

Locomotion-evoked hemodynamic responses depend on local neural activity, not cardiovascular responses.

Related to **Fig.3**a-d. (**a**) An image of a polished thin-skull window with cannula and electrode implants. The yellow shaded area indicates the area affected by the drug infusion as determined from electrical recording. This area is used as the ROI for quantification of hemodynamic signals. (**b**) Locomotion-evoked spatial distribution of $\Delta R/R_0$ (left) and HbO-HbR (right) for the same mouse shown in **Fig.3**a-c, following intracerebral infusion of aCSF

(n = 12 locomotion events), CNQX/AP5/muscimol (n = 14 locomotion events) and L-NAME (n = 12 locomotion events), as well as intraperitoneal injection of water (n = 8 locomotion events), atenolol (n = 13 locomotion events) and glycopyrrolate (n = 8 locomotion events). (**c**) Locomotion-evoked response of gamma-band power, MUA power, heart rate, $\Delta R/R_0$ and HbO-HbR following intracerebral infusion of aCSF, CNQX/AP5/muscimol and L-NAME in the same animal shown in (**b**). (**d**) As (**c**) but for responses following intraperitoneal injection of water, atenolol and glycopyrrolate. (**e**) Group average (n = 4 mice) of locomotion-evoked $\Delta R/R_0$ (left) and HbO-HbR (right) following different drug administration. Note that only disruption of neural activity (CNQX/AP5/muscimol infusion) caused changes in locomotion-evoked vasodilation or oxygenation, while glycopyrrolate and atenolol had large effects on heart rate. This shows that the oxygenation responses observed here are not affected by cardiovascular changes. Data are shown as mean ± SEM in (**c**), (**d**) and (**e**).

Supplementary Fig. 6

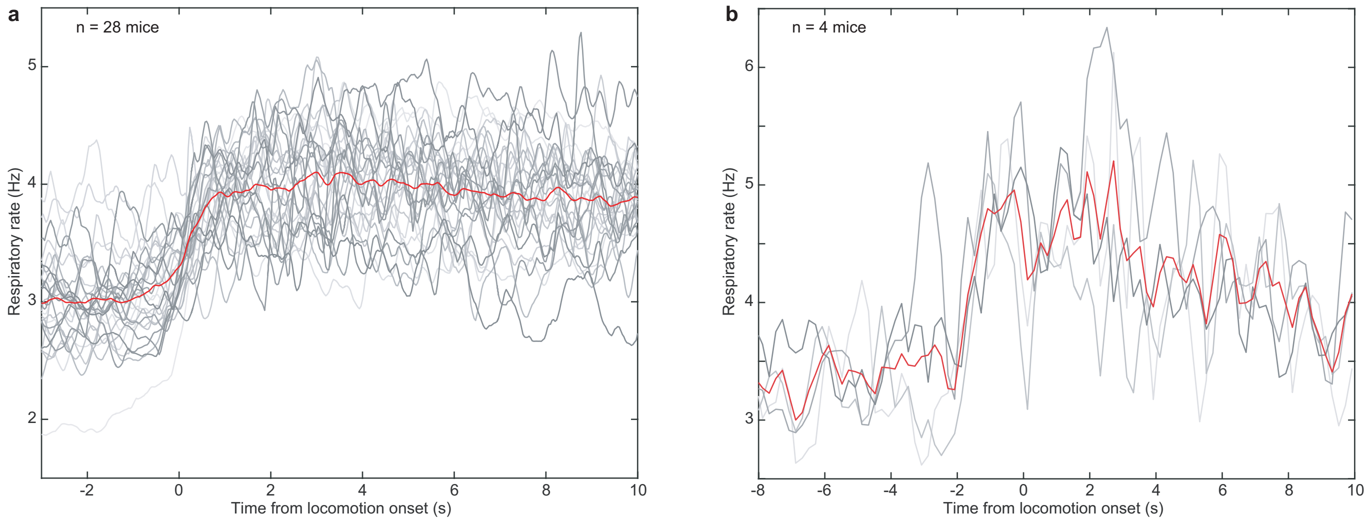

Measuring respiration with a thermocouple.

Related to **Fig.4**. (**a**) Respiratory rate at rest and during locomotion (n = 28 mice) for mice running on the spherical treadmill. Each gray trace indicates the averaged respiratory rate from one mouse (~ 2 h recording), and the red trace indicates group average. Time zero indicates onset of locomotion. (**b**) As in (**a**) but for mice (n = 4) running on the rotating disk.

Supplementary Fig. 7

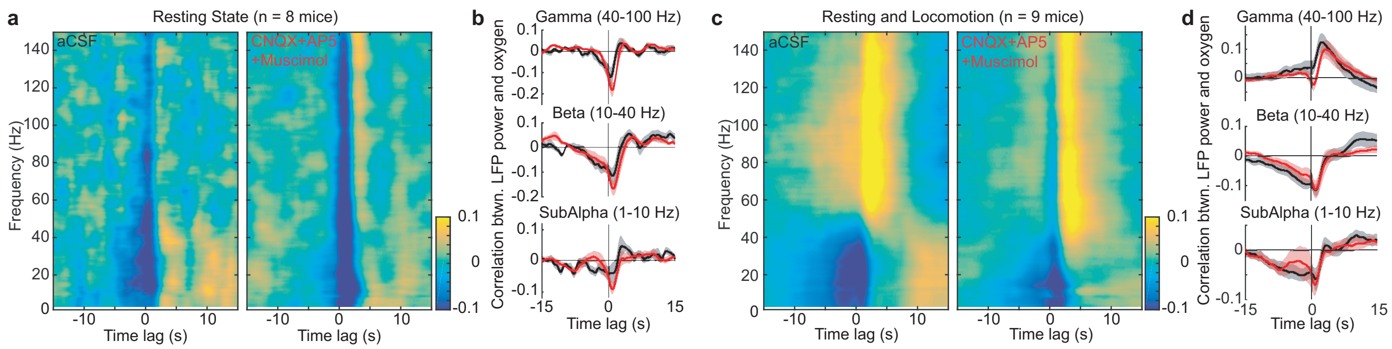

Suppressing vasodilation does not change the correlation between oxygenation and neural activity.

Related to **Fig.4**a-e. (**a**) Group average (n = 8 mice) of cross-correlation between PtO_2 and LFP at various frequency band during periods of rest after aCSF (left) and CNQX/AP5/muscimol (right) administration. (**b**) Cross-correlation between PtO_2 and LFP at different frequency band during periods of rest after aCSF (black) and CNQX/AP5/muscimol (red) administration. The shaded region shows the population standard error of the mean (n = 8 mice). (**c**) As in (**a**) but for periods including both rest and locomotion (n = 9 mice). (**d**) As in (**b**) but for periods of both rest and locomotion (n = 9 mice). Data are shown as mean ± SEM in (**b**) and (**d**).

Supplementary Fig. 8

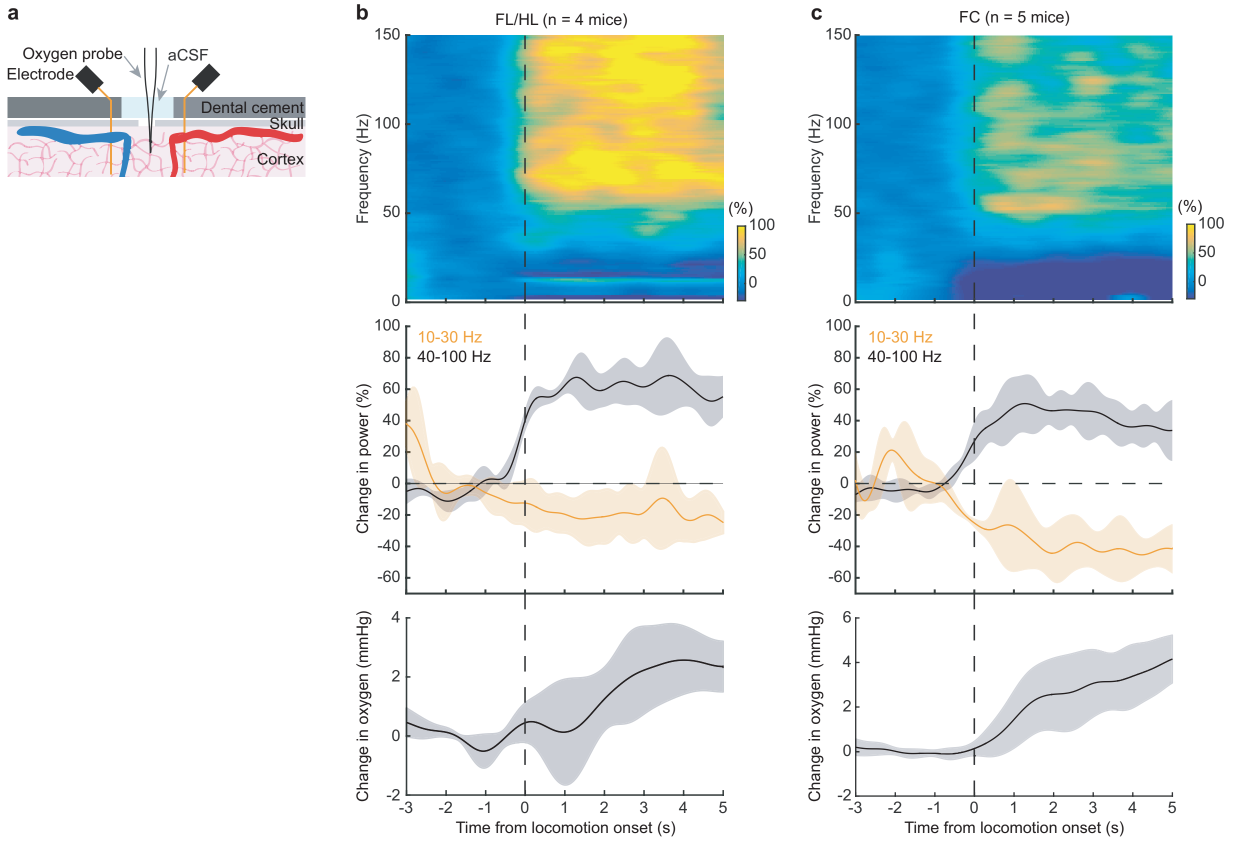

Locomotion-evoked LFP power changes in both FL/HL and FC.

(**a**) Schematic of experimental setup for simultaneous tissue oxygenation and electrophysiology measurement. (**b**) Top, time-frequency representation of locomotion-evoked changes in LFP power in FL/HL (n = 4 mice). Middle, locomotion-evoked changes of gamma-band (40-100 Hz, black) and beta-band (10-30 Hz, orange) power. Bottom, locomotion-evoked changes of tissue oxygenation. Data were shown as mean ± SD. (**c**) As (**b**) but for FC (n = 5 mice). The data used in (**b**) and (**c**) were from the same group of mice shown in **Fig.3**g-h with the craniotomy superfused with aCSF. Data are shown as mean ± SD in (**b**) and (**c**).

Supplementary Fig. 9

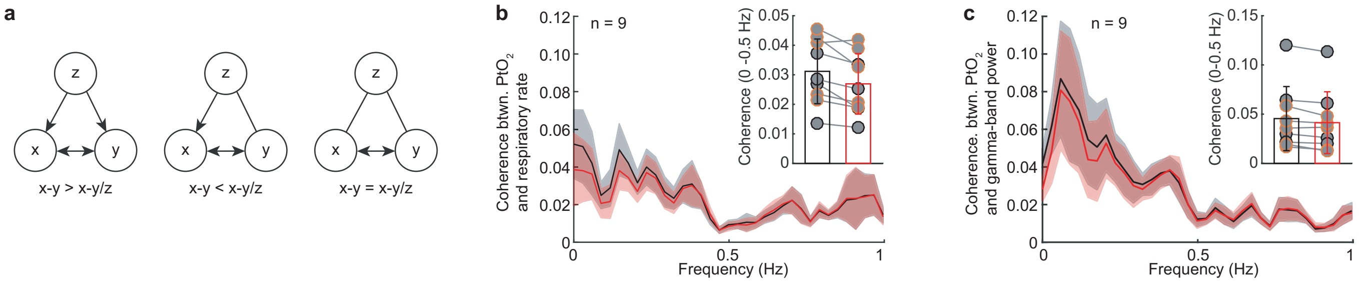

Respiration and neural activity modulate tissue oxygenation independently.

Related to **Fig.4**. (**a**) Schematic showing different patterns of coupling between three signals (x, y and z) that can be revealed by the partialization technique. Partialization with z (x-y/z) may decrease the x-y coherence if they are both connected to z, or even completely block the coherence if they are exclusively drive by signal z (left). Partialization with z may increase the x-y coherence if x and y are affected by z in an asymmetric manner (middle). Partialization with z may not affect the x-y coherence if x and y are not connected to z (right). (**b**) Group average of coherence between respiratory rate and PtO_2 before (black) and after (red) partializing the effect of neural activity. The inset denotes that group average of coherence within the frequency range of 0 to 0.5 Hz before (black) and after (red) partialization. (**c**) As in (**b**) but for coherence between gamma-band LFP power and PtO_2 before (black) and after (red) partializing the effect of respiratory rate. Solid lines and shaded area in (**b**) and (**c**) denote mean ± SEM, respectively. Data are shown as mean ± SD in all other graphs.

Supplementary Fig. 10

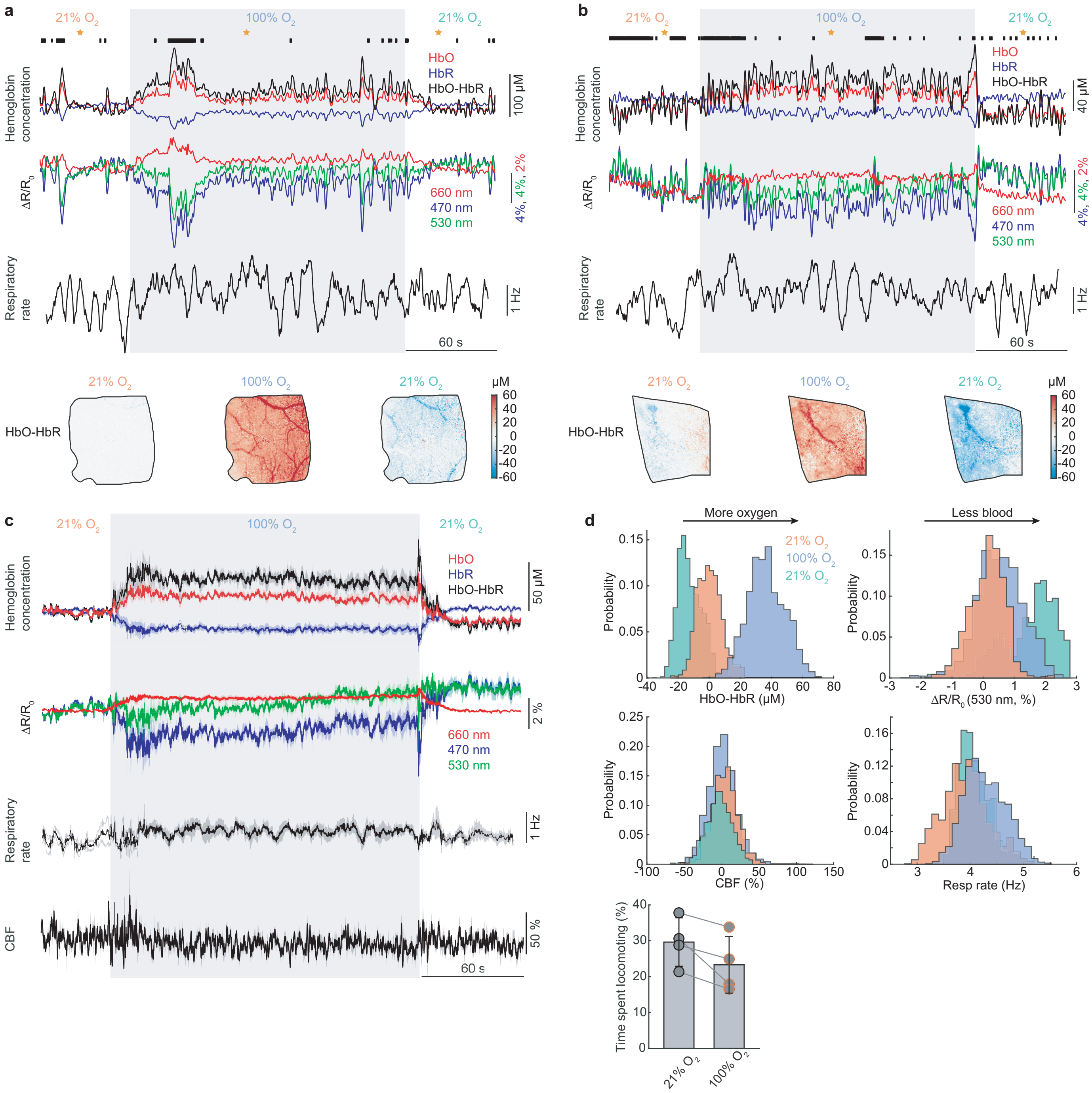

Cerebral oxygenation increases during oxygen challenge.

(**a**) Example trace showing the hemoglobin concentration (top) and $\Delta R/R_0$ (middle) change in the somatosensory cortex, as well as respiration rate (bottom) change for a single trial of 3 min 100% oxygen breathing (gray shaded area). Black ticks denote binarized locomotion events. Frames at the bottom of the figure showing the pixel-wise change of HbO-HbR before, during and after 100% oxygen breathing. The orange stars point to the resting period showing in each frame. (**b**) As in (**a**) but for a single trial in the frontal cortex. (**c**) Group average (n = 4 mice, 2 in the somatosensory cortex and 2 in the frontal cortex) of hemoglobin concentration, $\Delta R/R_0$, respiration

rate and CBF responses to 100% oxygen breathing. Shaded area indicates 1 SEM. (**d**) Histograms of HbO-HbR, $\Delta R/R_0$, CBF and respiration rate before, during and after 100% oxygen breathing. Compared to 21% oxygen breathing, HbO-HbR were greater (two-sample Kolmogorov-Smirnov test, $p < 0.0002$), $\Delta R/R_0$ (two-sample Kolmogorov-Smirnov test, $p = 0.9655$), CBF (two-sample Kolmogorov-Smirnov test, $p = 0.1350$) and respiration rate (two-sample Kolmogorov-Smirnov test, $p = 0.7710$) were not different during 100% oxygen breathing. Note that the time mice spent locomoting is similar between 21% oxygen breathing and 100% oxygen breathing (paired t-test, $p = 0.0584$). Data are shown as mean ± SD in (**d**).

Supplementary Fig. 11

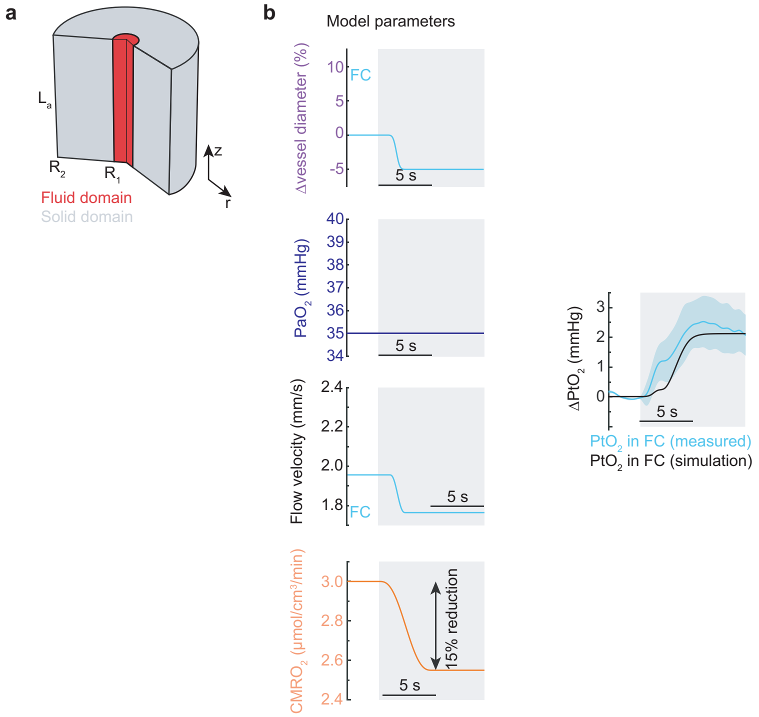

Locomotion-evoked tissue oxygenation increase in the frontal cortex cannot be explained by reduction of $CMRO_2$.

Related to **Fig.5**. (**a**) Schematic showing the model geometry. (**b**) To answer if only $CMRO_2$ reduction would be possible to induce the locomotion-evoked increase of PtO_2 in the frontal cortex, we kept the arterial oxygen tension constant and the arterial diameter is changed the same way as shown in **Fig.5**c, and asked how much $CMRO_2$ would need to drop to get the similar PtO_2 dynamics as observed experimentally. A 15% reduction in $CMRO_2$ is required to match the observed increase in oxygen tension in frontal cortex.

Supplementary Tables

Supplementary Table 1. Model parameters

Model parameter	Description	Value	Source
DO_2	Oxygen diffusivity	2800 $\mu m^2 s^{-1}$	1
R_1	vessel radius	9 μm	
R_2	Radius of tissue cylinder	50 μm	2,3
L_a	Axial length	100 μm	
μ_f	Blood viscosity	0.004 Pa·s	4
μ_s	Brain tissue shear modulus	4 kPa	5-7
$CMRO_{2,baseline}$	Resting $CMRO_2$	3 $\mu mole \cdot cm^{-3} \cdot min^{-1}$	8
$CMRO_{2,MAC}$	$CMRO_2$ after CNQX/AP5/muscimol application	1.2 $\mu mole \cdot cm^{-3} \cdot min^{-1}$	
$PO_{2,baseline}$	Resting arterial PO_2	35 mmHg	9
ρ	solubility coefficient for O_2	1.39 $\mu M \cdot mmHg^{-1}$	2,9,10
**Locomotion-evoked dynamics**			
$\mathbf{PO_{2,art}} = \begin{cases} \mathbf{PO_{2,baseline}} & if\ t < \tau \\ \mathbf{PO_{2,baseline}} \left(A \frac{(t-\tau)^{\alpha-1} \beta^{\alpha} e^{-\beta(t-\tau)}}{\Gamma(\alpha)} + 1 \right) & if\ t \geq \tau \end{cases}$			
A		1	
α		1.9	
β		0.3	
τ (time shift)		1 s	11
Locomotion-evoked change of vessel radius			
FL/HL		+10% (+0.9 μm)	
FC		-5% (-0.45 μm)	
CNQX/AP5/muscimol		-20% (-1.8 μm)	
Locomotion-evoked change of $CMRO_2$			
FL/HL		+15% (+0.45 $\mu mole/cm^3/min$)	12-14
FC		+4% (+0.12 $\mu mole/cm^3/min$)	

Supplementary References

1 Lamkin-Kennard, K. A., Buerk, D. G. & Jaron, D. Interactions between NO and O2 in the microcirculation: a mathematical analysis. *Microvasc Res* **68**, 38-50 (2004).

2 Sakadzic, S. *et al.* Two-photon microscopy measurement of cerebral metabolic rate of oxygen using periarteriolar oxygen concentration gradients. *Neurophotonics* **3**, 045005 (2016).

3 Linninger, A. A. *et al.* Cerebral microcirculation and oxygen tension in the human secondary cortex. *Ann Biomed Eng* **41**, 2264-2284 (2013).

4 Pries, A. R., Neuhaus, D. & Gaehtgens, P. Blood viscosity in tube flow: dependence on diameter and hematocrit. *Am J Physiol* **263**, H1770-1778 (1992).

5 Franceschini, G., Bigoni, D., Regitnig, P. & Holzapfel, G. A. Brain tissue deforms similarly to filled elastomers and follows consolidation theory. *Journal of the Mechanics and Physics of Solids* **54**, 2592-2620 (2006).

6 Budday, S. *et al.* Mechanical characterization of human brain tissue. *Acta Biomater* **48**, 319-340 (2017).

7 Weickenmeier, J. *et al.* Brain stiffens post mortem. *J Mech Behav Biomed Mater* **84**, 88-98 (2018).

8 Ni, R., Rudin, M. & Klohs, J. Cortical hypoperfusion and reduced cerebral metabolic rate of oxygen in the arcAbeta mouse model of Alzheimer's disease. *Photoacoustics* **10**, 38-47 (2018).

9 Sakadzic, S. *et al.* Two-photon high-resolution measurement of partial pressure of oxygen in cerebral vasculature and tissue. *Nat Methods* **7**, 755-759 (2010).

10 Goldman, D. Theoretical models of microvascular oxygen transport to tissue. *Microcirculation* **15**, 795-811 (2008).

11 Merkle, C. W. & Srinivasan, V. J. Laminar microvascular transit time distribution in the mouse somatosensory cortex revealed by Dynamic Contrast Optical Coherence Tomography. *Neuroimage* **125**, 350-362 (2016).

12 Germuska, M. *et al.* Dual-calibrated fMRI measurement of absolute cerebral metabolic rate of oxygen consumption and effective oxygen diffusivity. *Neuroimage* **184**, 717-728 (2018).

13 Leontiev, O., Dubowitz, D. J. & Buxton, R. B. CBF/CMRO2 coupling measured with calibrated BOLD fMRI: sources of bias. *Neuroimage* **36**, 1110-1122 (2007).

14 Lin, A. L. *et al.* Evaluation of MRI models in the measurement of CMRO2 and its relationship with CBF. *Magn Reson Med* **60**, 380-389 (2008).
