# Supplementary material for: Cerebral oxygenation during locomotion is modulated by respiration
Source: Nat Commun. 2019 Dec 4;10:5515. doi: 10.1038/s41467-019-13523-5 (PMC6893036; doi:10.1038/s41467-019-13523-5)
Supplement: Supplementary file 3 — Reporting Summary [file 41467_2019_13523_MOESM3_ESM.pdf]

Corresponding author(s): Patrick J. Drew

Last updated by author(s): Nov 8, 2019

Reporting Summary

Nature Research wishes to improve the reproducibility of the work that we publish. This form provides structure for consistency and transparency in reporting. For further information on Nature Research policies, see Authors & Referees and the Editorial Policy Checklist.

Statistics

For all statistical analyses, confirm that the following items are present in the figure legend, table legend, main text, or Methods section.

n/a	Confirmed	
☐	☒	The exact sample size (n) for each experimental group/condition, given as a discrete number and unit of measurement
☐	☒	A statement on whether measurements were taken from distinct samples or whether the same sample was measured repeatedly
☐	☒	The statistical test(s) used AND whether they are one- or two-sided<br>*Only common tests should be described solely by name; describe more complex techniques in the Methods section.*
☐	☒	A description of all covariates tested
☐	☒	A description of any assumptions or corrections, such as tests of normality and adjustment for multiple comparisons
☐	☒	A full description of the statistical parameters including central tendency (e.g. means) or other basic estimates (e.g. regression coefficient) AND variation (e.g. standard deviation) or associated estimates of uncertainty (e.g. confidence intervals)
☐	☒	For null hypothesis testing, the test statistic (e.g. F, t, r) with confidence intervals, effect sizes, degrees of freedom and P value noted<br>*Give P values as exact values whenever suitable.*
☒	☐	For Bayesian analysis, information on the choice of priors and Markov chain Monte Carlo settings
☒	☐	For hierarchical and complex designs, identification of the appropriate level for tests and full reporting of outcomes
☒	☐	Estimates of effect sizes (e.g. Cohen's d, Pearson's r), indicating how they were calculated

Our web collection on statistics for biologists contains articles on many of the points above.

Software and code

Policy information about availability of computer code

Data collection	Custom code written in LabVIEW (version 11.0, 64-bit Windows 7, National Instruments) was used for data collection.
Data analysis	Custom code written in Matlab (2015a) was used to analyze the data and is available for download.<br>Both COMSOL and Matlab was used for computational modeling of oxygen transport and consumption in blood and brain parenchyma.

For manuscripts utilizing custom algorithms or software that are central to the research but not yet described in published literature, software must be made available to editors/reviewers. We strongly encourage code deposition in a community repository (e.g. GitHub). See the Nature Research guidelines for submitting code & software for further information.

Data

Policy information about availability of data

All manuscripts must include a data availability statement. This statement should provide the following information, where applicable:

- Accession codes, unique identifiers, or web links for publicly available datasets
- A list of figures that have associated raw data
- A description of any restrictions on data availability

The data and code used to generate the figures in this paper is available at https://psu.app.box.com/v/Zhang-O2-during-behavior.

Field-specific reporting

Please select the one below that is the best fit for your research. If you are not sure, read the appropriate sections before making your selection.

☒ Life sciences ☐ Behavioural & social sciences ☐ Ecological, evolutionary & environmental sciences

Life sciences study design

All studies must disclose on these points even when the disclosure is negative.

Sample size	Sample sizes were chosen to be consistent with previous studies.
Data exclusions	Animals which did not show significant neural activity reduction following pharmacological infusion/superfusion were not analyzed.<br>For infusion experiments all data taken after any aberrant neural activity or CBV were omitted from analysis.<br>For experiments quantifying arterial oxygen tension change with the phase of respiratory cycle, measurements did not pass the phase randomization test were excluded.
Replication	All animals showed similar oxygen and CBV responses to locomotion.
Randomization	For tissue oxygenation measurements using polarographic electrodes across the cortex, the measurements is always from the surface to the deep layers of the cortex.<br>For drug infusion through the cannula, infusion orders were counter-balanced so all mice received all treatments in alternating orders to mitigate effects of imaging sessions on one treatment, therefore no randomization was performed.<br>For drug superfusion over the craniotomy, we applied aCSF and collected data, and then applied CNQX/AP5/muscimol and collected data.
Blinding	No blinding took place during all the experiments.<br>Analysis of the data was performed using an automated analysis script with minimal experimenter input.

Reporting for specific materials, systems and methods

We require information from authors about some types of materials, experimental systems and methods used in many studies. Here, indicate whether each material, system or method listed is relevant to your study. If you are not sure if a list item applies to your research, read the appropriate section before selecting a response.

Materials & experimental systems

n/a	Involved in the study
☒	☐ Antibodies
☒	☐ Eukaryotic cell lines
☒	☐ Palaeontology
☐	☒ Animals and other organisms
☒	☐ Human research participants
☒	☐ Clinical data

Methods

n/a	Involved in the study
☒	☐ ChIP-seq
☒	☐ Flow cytometry
☒	☐ MRI-based neuroimaging

Animals and other organisms

Policy information about studies involving animals; ARRIVE guidelines recommended for reporting animal research

Laboratory animals	Mice were C57BL6J and Thy1-GCaMP6f acquired from Jackson Laboratory. Both male and female mice were used between the ages of 3-12 months.
Wild animals	The study did not involve wild animals.
Field-collected samples	The study did not involve samples collected from the field.
Ethics oversight	All experimental procedures were approved by the Pennsylvania State University and INSERM Institutional Animal Care and Use Committee guidelines.

Note that full information on the approval of the study protocol must also be provided in the manuscript.
