# Supplementary material for: Cerebral oxygenation during locomotion is modulated by respiration
Source: Nat Commun. 2019 Dec 4;10:5515. doi: 10.1038/s41467-019-13523-5 (PMC6893036; doi:10.1038/s41467-019-13523-5)
Supplement: Supplementary file 2 — Peer Review [file 41467_2019_13523_MOESM2_ESM.pdf]

Editorial Note: Parts of this peer review file have been redacted as indicated to remove third-party material where no permission to publish could be obtained.

Reviewers' comments:

Reviewer #1 (Remarks to the Author):

The manuscript “Cerebral oxygenation during locomotion is modulated by respiration” by Zhang et al. is outstanding on several levels. The findings are highly significant with broad reaching implications to the study of cerebrovasculature, neurovascular coupling, brain metabolism and functional brain imaging techniques. The experimental approaches are thorough, multifaceted and elegant. The data presentation is beautiful, and the analysis of complex data sets advanced, powerful and convincing. I was surprised by the main finding that respiration rate is a key driver of cerebral oxygenation and that several fundamental concepts regarding gamma band, vasodilation and systemic cardiac output/blood pressure can operate independent from each other and that they are not the most important predictive variables in determining brain PO2 during locomotion. I have one major comment below for the authors to consider as it would improve the manuscript and a few minor points.

Major
Though the conclusions drawn from the data and analysis are logical and convincing, one short coming is that there are no experiments of necessity and sufficiency that respiration is a primary driver of brain parenchyma PO2. This paper elegantly rules out the other likely possibilities and provides a very strong case that it must be respiration, but the causal experiments are missing. Is it feasible to directly increase or decrease the respiration rate and measure the predicted changes in PO2? What about injecting an AAV Gi-DREADD into the brain stem in either the Pre-Botz or RTN and then during the physiology experiment give systemic CNO or Compound 21 to decrease the respiration rate? Perhaps the authors have reasons not to try such an experiment? Lack of expertise?

Minor:
The authors have a data set at the end of the paper where they state that 4 out of 7 arteries showed a relationship between PaO2 and the respiratory cycle (fig 4j). It sounds like the other 3 experiments did not show this relationship, but it was not clear from the writing. Is there a valid reason not to include these 3 here? Was it because these 3 did not have a regular breathing cycle and could not be analyzed? If not, and these 3 were included, and the full data set was examined, does the overall relationship/significance disappear? I am not comfortable with arbitrarily binarizing the data unless there is an objective reason to do so. If this dataset was removed from the paper, it would not limit my enthusiasm.

I would prefer the term “penetrating artery” be “penetrating arteriole”. Maybe this is a simple matter that the authors use the term ‘artery’ in a general sense, but given the size of the penetrating vessels in the neocortex they definitely qualify as arterioles.

The authors surmise that autoregulation at the circle of Willis would control brain blood flow increases in the FC when cardiac output increases. Why just the circle of Willis? Wouldn’t all resistance vasculature undergo a myogenic response when pressure increases? Perhaps this argument needs to be refined.

A significant portion of the discussion is redundant with the results text. I like having a little ‘discussion’ in the results to convince the reader as they are going through the data, but I think it is best not to repeat the same points in the discussion section. Can the authors instead add to the discussion by speculating on the significance of their finding towards functions functional imaging, like IOS or fMRI?

Related comment: it would be fascinating to know whether this phenomenon applies to larger organisms, as there may be significant implications for functional optical and magnetic-based imaging in humans and non-human primates. I know the authors argued that it will apply, but there is no data yet, as expected. This could be fascinating for future work.

Reviewer #2 (Remarks to the Author):

Zhang et al. investigate the influence of breathing on cerebral oxygenation during locomotion in awake head-fixed mice. Using imaging, spectroscopic, neurophysiological and polarographic approaches to investigate indices of cerebral perfusion, brain pO2, and neural activity, they found that locomotion increases oxygenation in neocortical regions irrespective of their involvement in locomotion, and in the olfactory bulb. The increase was independent of neural activity and of the associated cerebrovascular changes, but was correlated with the breathing pattern and arterial pO2. It is concluded that breathing is able to independently influence cerebral oxygenation.

This paper raises of number of concerns related to the lack of direct measurement of relevant physiological parameters that are critical for the interpretation of the findings. The lack of these critical measurements render data interpretation excessively speculative and uncertain.

1. Arterial blood pressure (AP) was not measured. As mentioned in the paper, AP changes with locomotion and its dynamic impact on cerebrovascular parameters has profound implication for oxygen delivery.
2. Although "vasodilation" is mentioned extensively in the paper, vascular diameter and RBC flux were not measured. Therefore, it is unclear whether the indirect spectrophotometric indices used to assess hemodynamic factors accurately reflect vascular variables.
3. Oxygen utilization and extraction fraction were not measured. These variables are critical for the interpretation of changes in interstitial pO2 and for the adjustments in oxygen delivery occurring during locomotion. Changes in O2 utilization could occur in the absence of changes in interstitial pO2.
4. Changes in pC02 were not recorded, which can have profound effects on blood flow and oxygen delivery.
5. Many of the observations were made in a limited number of mice (e.g., n=2) and considering the biological variability of the awake preparation more robust data is needed to draw conclusions in this experimental preparation.
6. Some of the interpretations do not seem to agree with the data. For example, it is said that locomotion decreases CBF and CBV in the frontal cortex (page 5, line 98), but the related figure (fig 1d) seem to show no significant changes in these variables.
7. Irrespective of these reservations, the conclusions of the study are rather expected: there is a global increase in brain oxygenation which is linked to the known variation in pO2 occurring with breathing.
8. "Breathing" may be more appropriate then "respiration".

Reviewer #3 (Remarks to the Author):

This is a very interesting manuscript that addresses the important link and dissociation between systemic oxygen delivery to the brain tissue caused by locomotion and respiration and hyperemia/neurovascular coupling induced changes in blood and tissue oxygenation. The authors present an impressive collection of results, which are based on multimodal experimental assays. The experiments are performed on the highest level, using top-notch technology in the awake behaving mouse. The manuscript follows a very stringent line of thought and the results are clear and well presented.

I have a few major concerns:

1. The work lacks an experimental approach in which brain oxygenation is increased without

locomotion. This is needed to nail down the paper's main claims. The breathing cycle experiment is very elegant and goes in that direction, but it is not sufficient. A good additional experiment would be 100% oxygen breathing. This would give the paper also a good translational twist, as this is used in humans (e.g. c.f. Fan et al., 2016. Neuroimage 125, 920-93.).

2. The paper neglects completely the implications for human fMRI and it also neglects all the work that has been done in humans using hyperoxic challenges (see above as an example). The introduction and discussion needs to be completely redone, with a clear focus on why these results are important for hemodynamically-based neuroimaging studies. I do not doubt the relevance, but the authors have simply not done a good job in linking the results with the wider field of functional neuroimaging. As it stands, it is hard to see for an outsider why this is important and why the experiments were done at all.

3. The paper concentrates on CBV and CBF should be considered equally too.

4. Depth variations of pO2 are obvious, but the authors do not correlate this to depth resolved hemodynamic measurements.

5. CBF aspects are not considered in the modeling. This is problematic because hemodynamic regulation through constrictions/dilations come with changes in CBF and a pure O2 diffusion model comes short.

6. Does the tissue cylinder stay constant in case of vessel diameter changes? This might be problematic, because a diameter increase/decrease will lead to a tissue cylinder decrease/increase and hence a decreased/increased O2 need. Please check.

7. Please discuss the fact that the different readouts potentially probe different cortical depths. IOS has a surface bias, etc.

8. Figure 1d. The authors mention a CBF decrease in FC, but I see an increase in FL/HL and much less so also in FC. Please clarify.

Reviewers' comments:

Reviewer #1 (Remarks to the Author):

The manuscript “Cerebral oxygenation during locomotion is modulated by respiration” by Zhang et al. is outstanding on several levels. The findings are highly significant with broad reaching implications to the study of cerebrovasculature, neurovascular coupling, brain metabolism and functional brain imaging techniques. The experimental approaches are thorough, multifaceted and elegant. The data presentation is beautiful, and the analysis of complex data sets advanced, powerful and convincing. I was surprised by the main finding that respiration rate is a key driver of cerebral oxygenation and that several fundamental concepts regarding gamma band, vasodilation and systemic cardiac output/blood pressure can operate independent from each other and that they are not the most important predictive variables in determining brain PO2 during locomotion. I have one major comment below for the authors to consider as it would improve the manuscript and a few minor points.

Figure A, (adapted from Yackle, Science 2017). The respiratory rhythm generator projects to Cdh9/Dbx+ neurons, that then project to the locus coeruleus (LC). Any perturbation of activity in the pre-BotC will cause brain-wide changes in neural activity, metabolism, and blood flow. Stimulation VRG premotor neurons will conflict with the rhythmic drive coming out of the preBotC, so is not a candidate for stimulation.

Major
Though the conclusions drawn from the data and analysis are logical and convincing, one short coming is that there are no experiments of necessity and sufficiency that respiration is a primary driver of brain parenchyma PO2. This paper elegantly rules out the other likely possibilities and provides a very strong case that it must be respiration, but the causal experiments are missing. Is it feasible to directly increase or decrease the respiration rate and measure the predicted changes in PO2? What about injecting an AAV Gi-DREADD into the brain stem in either the Pre-Botz or RTN and then during the physiology experiment give systemic CNO or Compound 21 to decrease the respiration rate? Perhaps the authors have reasons not to try such an experiment? Lack of expertise?

We agree with the reviewer that manipulations of the activity of the respiratory-driving preBotzinger complex (Pre-BotC) would be a “killer” experiment. However, there are issues that would make the results of such an experiment difficult, if not impossible, to interpret. Recent work (Yackle et al., 2017, Science; Yang and Feldman, J. Comp Neuro 2018) has elegantly shown that the respiratory CPG drives a subset of pre-BotC neurons that directly excite the locus coeruleus (see **Figure A** below, adapted from Yackle, Figure 4H for schematic), which then drives brain-wide changes in neural activity and other changes in metabolism. Stimulation of the locus coeruleus (LC) releases norepinephrine, which has a multitude of effects, including activation of astrocytes (Paukert et al. 2014, Neuron), changes in neural activity and blood flow (Toussay et al., 2014 J. Neurosci.), permeability of the blood brain barrier (Raichle et al., 1975, PNAS), and causes large changes in metabolism and glucose uptake (Craik et al, 1987, Brain research bulletin; Abraham et al., Brain research 1979). Thus, manipulating the activity of pre-BotC neurons (or the regions that project to it, such as RTN) would have the side effect of driving brain-wide changes in neural activity and metabolism in addition to the desired effects on breathing. It would be very hard to interpret the results of this experiment.

Finally, we note that we did not mean to imply respiration is the “primary driver” of parenchymal pO2, only that it was a contributor.

Minor:
The authors have a data set at the end of the paper where they state that 4 out of 7 arteries showed a relationship between PaO2 and the respiratory cycle (fig 4j). It sounds like the other 3 experiments did not

show this relationship, but it was not clear from the writing. Is there a valid reason not to include these 3 here? Was it because these 3 did not have a regular breathing cycle and could not be analyzed? If not, and these 3 were included, and the full data set was examined, does the overall relationship/significance disappear? I am not comfortable with arbitrarily binarizing the data unless there is an objective reason to do so. If this dataset was removed from the paper, it would not limit my enthusiasm.

The respiration was not as regular in some trials as others. This can be clarified in a revised version of the paper.

I would prefer the term “penetrating artery” be “penetrating arteriole”. Maybe this is a simple matter that the authors use the term ‘artery’ in a general sense, but given the size of the penetrating vessels in the neocortex they definitely qualify as arterioles.

Agreed. This can be easily fixed in a revised version of the paper.

The authors surmise that autoregulation at the circle of Willis would control brain blood flow increases in the FC when cardiac output increases. Why just the circle of Willis? Wouldn’t all resistance vasculature undergo a myogenic response when pressure increases? Perhaps this argument needs to be refined.

The reviewer is correct that the resistance arterioles could play a role (Willie et al., 2014, J Physiology), recent evidence has suggested in humans that only vessels in the Circle of Willis constrict (Warnert et al.,2016 JCBFM). The brevity in this aspect of the discussion can easily be corrected.

A significant portion of the discussion is redundant with the results text. I like having a little ‘discussion’ in the results to convince the reader as they are going through the data, but I think it is best not to repeat the same points in the discussion section. Can the authors instead add to the discussion by speculating on the significance of their finding towards functions functional imaging, like IOS or fMRI?

This can be corrected in a revised version of the paper.

Related comment: it would be fascinating to know whether this phenomenon applies to larger organisms, as there may be significant implications for functional optical and magnetic-based imaging in humans and non-human primates. I know the authors argued that it will apply, but there is no data yet, as expected. This could be fascinating for future work.

We completely agree with the reviewer on this point.

Reviewer #2 (Remarks to the Author):

Zhang et al. investigate the influence of breathing on cerebral oxygenation during locomotion in awake head-fixed mice. Using imaging, spectroscopic, neurophysiological and polarographic approaches to investigate indices of cerebral perfusion, brain pO_2, and neural activity, they found that locomotion increases oxygenation in neocortical regions irrespective of their involvement in locomotion, and in the olfactory bulb. The increase was independent of neural activity and of the associated cerebrovascular changes, but was correlated with the breathing pattern and arterial pO_2. It is concluded that breathing is able to independently influence cerebral oxygenation.

This paper raises of number of concerns related to the lack of direct measurement of relevant physiological parameters that are critical for the interpretation of the findings. The lack of these critical measurements render data interpretation excessively speculative and uncertain.

1. Arterial blood pressure (AP) was not measured. As mentioned in the paper, AP changes with locomotion and its dynamic impact on cerebrovascular parameters has profound implication for oxygen delivery.

The question here is not whether blood pressure changes during locomotion (it does), but whether a change in blood pressure could account for the increase in oxygenation that we see in the frontal cortex, where there is

no increase in blood flow. Through a series of experiments, we showed that the change in oxygenation cannot be accounted for by blood pressure changes.

The blood flow of the brain will depend on the resistance of the brain vasculature, the blood pressure, and the resistance of the rest of the body's vasculature. If blood pressure rises without any changes in vessel diameter, the flow of the blood to the brain (as measured with laser Doppler) will rise. Thus there is the possibility that increases in arterial blood pressure could raise oxygenation in the tissue, but the pressure-induced increase in oxygenation is mediated by an increase in local blood flow.

The ways to test if blood flow in the brain is affected by changes in arterial blood pressure are to 1) measure blood flow directly and 2) manipulate blood pressure to disrupt the blood flow mediated changes in oxygenation. **We reported the results of both of these experiments in the manuscript**. Using laser Doppler flowmetry, a measure of blood flow (Shih et al., JCBFM 2009), we found that there was no increase in blood flow to the frontal cortex. This categorically rules out the possibility that blood flow increases mediated by blood pressure drive the increase in oxygenation we see. The lack of flow increase is likely because blood vessels at the level of the circle of Willis constrict during exercise (Warnert et al, 2016, JCBFM), buffering the effects of increased blood pressure in the brain, though there are likely other mechanism that contribute. Secondly, we pharmacologically increased and decreased blood pressure with atenolol and glycopyrrolate respectively (which do not cross the blood brain barrier) and saw no change in oxygenation (**Supplementary Figure 5**), replicating previous results from our lab which showed that modulations of systemic blood pressure do not affect blood flow in the frontal cortex (Huo, Green and Drew, Neuroimage 2015, Figure 3). Again, this result is completely inconsistent with the hypothesis that the blood pressure fluctuations have any impact on brain oxygenation.

Lastly, we observed increases in both tissue and arterial oxygenation when the respiratory rate varies in a stationary animal (Fig 4b and 4f). These results cannot be explained by blood pressure variations.

We discussed this possibility in the discussion, quoted below:
*"Respiration is not the only physiological change that accompanies exercise, and it bears considering other mechanisms that could account for the cerebral and arterial oxygenation changes seen here. Exercise causes large changes in cardiac output and blood pressure, and can be accompanied by changes in blood CO_2 and lactate levels, but we think they are unlikely to be the cause of the nonspecific increase in cerebral oxygenation that we saw here. First, for the increases in cardiac output to raise global oxygenation in the cortex (independent of any changes in systemic oxygenation), it would need to drive an increase in cerebral blood flow. Our laser Doppler experiments show that blood flow does not rise in the frontal cortex, as they are likely buffered by autonomic regulation of the circle of Willis. Additionally, when heart rate and blood pressure increases during locomotion were blocked (with the beta blocker atenolol, which does not cross the blood brain barrier) or occluded (with the muscarinic receptor antagonist glycopyrrolate which also does not cross the blood brain barrier), there was no change in the locomotion-evoked CBV change (**Supplementary Fig. 5**, see also[49]). Therefore, systemic cardiac output increase cannot explain the increases in cerebral oxygenation seen during locomotion."*

The reviewer's critique does not acknowledge any of the several experiments presented in the paper, or arguments present at length in the submitted manuscript.

2. Although "vasodilation" is mentioned extensively in the paper, vascular diameter and RBC flux were not measured. Therefore, it is unclear whether the indirect spectrophotometric indices used to assess hemodynamic factors accurately reflect vascular variables.

We measured RBC flux with laser Doppler flowmetry (Fig 1C and D, Supplementary Figure 1). This is a standard technique (Shih et al., JCBFM 2012).

Hemoglobin is the strongest absorber of visible light in the brain. Intrinsic signal imaging using one or more wavelengths of light is widely used to assay vessel dilation. As dilating a vessel will increase the local

concentration of hemoglobin, and hemoglobin concentration is far and away the largest determinant of light reflectance from the brain.

Intrinsic imaging has been used by multiple labs to measure vessel dilation (with validation with 2-photon microscopy) in work by the Kleinfeld lab (Mateo et al., 2017, Neuron), Elizabeth Hillman's lab (Ma et al., 2016, PNAS) and by ourselves (Gao, Huo, Drew, Neuroimage 2015). We have shown that arterial and venous dilations during locomotion (as measured directly with 2-photon microscopy) cause corresponding decreases in reflectance in green light in the intrinsic optical signal (Gao, Huo, Drew, Neuroimage 2015). We have also made extensive measurements of vascular diameters with two photon microscopy during locomotion and during other conditions (Drew et al., 2011, PNAS, Gao and Drew, 2016 J Neuroscience; Winder et al. 2017 Nature Neuroscience), and these measurements very closely match intrinsic optical signal measurements. *Thus, changes in reflectance have been validated by multiple labs to be robust measures of vascular dilation.*

The correspondence between vascular dilation and the intrinsic signal imaging was addressed in our manuscript (page 4):
"When the brain is illuminated with 530 nm light, reflectance decreases report dilations of arteries, capillaries and veins, which correspond with increases in cerebral blood volume (CBV). This reflectance change observed with IOS closely tracks measurements of vessel diameter made with two-photon microscopy[26]. The consistency with microscopic measurements of vessel diameter, combined with its very high signal-to-noise ratio[25], and spatial resolution (less than 200 μm[27]), makes IOS suitable for detecting hemodynamic responses to locomotion."

3. Oxygen utilization and extraction fraction were not measured. These variables are critical for the interpretation of changes in interstitial pO2 and for the adjustments in oxygen delivery occurring during locomotion. Changes in O2 utilization could occur in the absence of changes in interstitial pO2.

For changes in oxygen utilization to explain the increase in tissue oxygenation we see during locomotion, the increase in neural activity (Figure 1 and Supplementary Figure 2) would have to be accompanied by a ***decrease*** in metabolic activity. This would make no sense, and we know of no publication in the brain metabolism literature supporting the idea that increase in neural activity are associated with a decrease in metabolic rate. It is well established that increases in neural activity cause increases in oxygen use (Logothetis, Nature, 2008; Buxton, Neuroimage, 2012). Unless the reviewer is proposing that the *increases* in neural activity we observed during locomotion can drive *decreases* in neural metabolism, there is no way that oxygen utilization can explain our observations.

The oxygen extraction fraction (OEF) is the fraction of oxygen extracted from the blood in its transit through the brain, and is determined by the metabolic rate and blood flow (Buxton, Neuroimage, 2012; Buxton, Frontiers in Neuroenergetics, 2010). It is dimensionless parameter that relates the oxygenation of the veins (which is what the BOLD contrast mechanism is sensitive to) to the oxygenation of the arteries. OEF is not measured directly, but rather can be calculated (with many assumptions) by measuring cerebral blood flow and oxygen use (cerebral metabolic rate of oxygen-CMRO2) using positron emission tomography (Buxton, Frontiers in Neuroenergetics, 2010). The OEF does not tell us anything quantitative about the quantities of interest, specifically the absolute values of arterial and tissue oxygenation, and it is not relevant to the experiments done here. This is why we made direct, quantitative measures of tissue and arterial oxygenation.

4. Changes in pC02 were not recorded, which can have profound effects on blood flow and oxygen delivery.

CO2 is a vasodilator (Cohen et al., JCBFM, 2002), and fluctuations in CO2 levels can affect arterial diameter and thus blood flow. Thus, CO2 levels could affect brain tissue oxygenation, mediated by an increase in blood flow. However, changes in pCO2 cannot explain the increases in oxygenation we see. First, CO2-mediated changes in vessel diameter are very slow, taking tens of seconds to occur, even when driven by inhalation of high levels of CO2 (Ngai and Winn, Amer. Journal Physiology, 1996), far too slow to account for the dilations that occur within a second of locomotion onset. Secondly, CO2 levels in the blood *fall* with the onset of exercise in rodents (Fregosy and Dempsy, Journal of Applied Physiology, 1985). This exercise associated decrease of CO2 would drive vasoconstriction, decreased blood flow, and consequently cause a *decrease* in

tissue oxygenation, the opposite of what we observed. Thus, changes in arterial CO2 *cannot* account for any of the oxygenation changes we see.

This issue was addressed in the discussion of the submitted manuscript:
"Second, while CO_2 is a strong vasodilator, and can drive increases in cerebral oxygenation under hypercapnia conditions by dilating blood vessels, rodents become hypocapnic during sustained exercise[70]. Exercise-evoked changes in CO_2 would tend to cause cerebral vasoconstriction and would tend to drive a deoxygenation. Again, this mechanism could not drive the observed increase in blood and tissue oxygenation in the frontal cortex without corresponding flow increases and vasodilation."

5. Many of the observations were made in a limited number of mice (e.g., n=2) and considering the biological variability of the awake preparation more robust data is needed to draw conclusions in this experimental preparation.

We used three different techniques (polarography, spectroscopy, and two-photon phosphorescence lifetime measurements of oxygen sensors) in a total of 78 mice used in our experiments. The smallest n in our paper is n=4 (for measurements of arterial O2 using 2-photon phosphorescence and some of our local intracranial infusion experiments). For Figure 3i, we measured from 5 surface arterioles in 4 mice (two large feeding arteries in the olfactory bulb and three in the somatosensory cortex, which we analyzed as a group as they have very similar oxygen responses). For this group, as we see a locomotion-induced oxygenation increase of ~7 mmHg above baseline, which has standard deviation of ~2 mmHg, this conservatively gives us an effect size of ~3. Post hoc calculation of power (with G*Power software, Faul et al, Behav. Research Methods, 2007) with an alpha of 0.05 gives us a 1-beta (power) of 0.99, more than the standard power of 0.8. Even with an effect size of 2 (consistent with what we see with the infusion experiments (Winder et al., 2017 Nature Neuro)) we still have a power > 0.90.

The so-called "variability" ascribed to the awake preparation is due to un-monitored behavior (locomotion, arousal fluctuations, grooming, whisking), as we and others have recently shown (see Winder et al., 2017 Nature Neuro; Drew, Winder, Zhang, 2019 Neuroscientist; Stringer et al., 2019 Science; Chang … Duyn, 2016 PNAS). These spontaneous movements drive neural activity and vasodilation. In our experiments, we carefully monitored behavior (locomotion and respiration), and aligned our responses to locomotion, so variability is minimal.

6. Some of the interpretations do not seem to agree with the data. For example, it is said that locomotion decreases CBF and CBV in the frontal cortex (page 5, line 98), but the related figure (fig 1d) seem to show no significant changes in these variables.

Because natural locomotion is of varying durations, we quantified CBF and CBV in two different ways. The first way was using the locomotion-triggered average, in which the responses to locomotion events between 5 and 10 seconds in duration separated from any previous locomotion event by 7 seconds are averaged together. This selects a subset of the total locomotion events, and is shown in Figure 1. The average locomotion-evoked CBF increase was not significantly greater than 0 ($p<0.22$). The second way is to calculate the hemodynamic response function (HRF), the linear filter that relates locomotion to CBF and CBV to locomotion (just as we do for oxygenation in Figure 2). The HRF method allows us to quantify the CBF and CBV responses to all locomotion events (short and long) together, and this is shown in Supplementary Figure 1. In principle, the HRF method is superior, but conceptually more complicated than the locomotion-triggered average. The HRF method shows a significant decrease in CBF and CBV in frontal cortex (Supplementary Figure 1). We apologize that our description of the differences between these methods was not clear, and this could be rectified in a resubmission.

7. Irrespective of these reservations, the conclusions of the study are rather expected: there is a global increase in brain oxygenation which is linked to the known variation in pO2 occurring with breathing.

The assumption in fMRI and neurovascular coupling studies is that the arterial blood is completely saturated with oxygen (Kim and Ogawa, 2012 JCBFM; Buxton, 2012, Rep. Prog Phys; Hillman, 2014, Annual Review of Neuroscience). If the arterial blood is saturated, then increasing respiration should not cause an increase in

arterial oxygenation, as it simply cannot take on any more oxygen. Our works shows this is not the case, at least for rodent models, which will be surprising to anyone familiar with the assumptions in the field.

8. "Breathing" may be more appropriate then "respiration".

This is not a major concern.

Reviewer #3 (Remarks to the Author):

This is a very interesting manuscript that addresses the important link and dissociation between systemic oxygen delivery to the brain tissue caused by locomotion and respiration and hyperemia/neurovascular coupling induced changes in blood and tissue oxygenation. The authors present an impressive collection of results, which are based on multimodal experimental assays. The experiments are performed on the highest level, using top-notch technology in the awake behaving mouse. The manuscript follows a very stringent line of thought and the results are clear and well presented.

I have a few major concerns:

1. The work lacks an experimental approach in which brain oxygenation is increased without locomotion. This is needed to nail down the paper's main claims. The breathing cycle experiment is very elegant and goes in that direction, but it is not sufficient. A good additional experiment would be 100% oxygen breathing. This would give the paper also a good translational twist, as this is used in humans (e.g. c.f. Fan et al., 2016. Neuroimage 125, 920-93.).

Although we observed increases in arterial oxygenation in the absence of locomotion (Figure 4f) and a significant correlation between tissue oxygenation and respiration rate in rest periods lacking locomotion (Figure 4b), we agree with the reviewer that this would be an interesting experiment that would add to the manuscript very much. This is a straightforward experiment that we have done before and would be happy to include to a resubmitted manuscript. Below is an example figure for our oxygen challenge results in a single animal showing that oxygen challenge increases oxygenation.

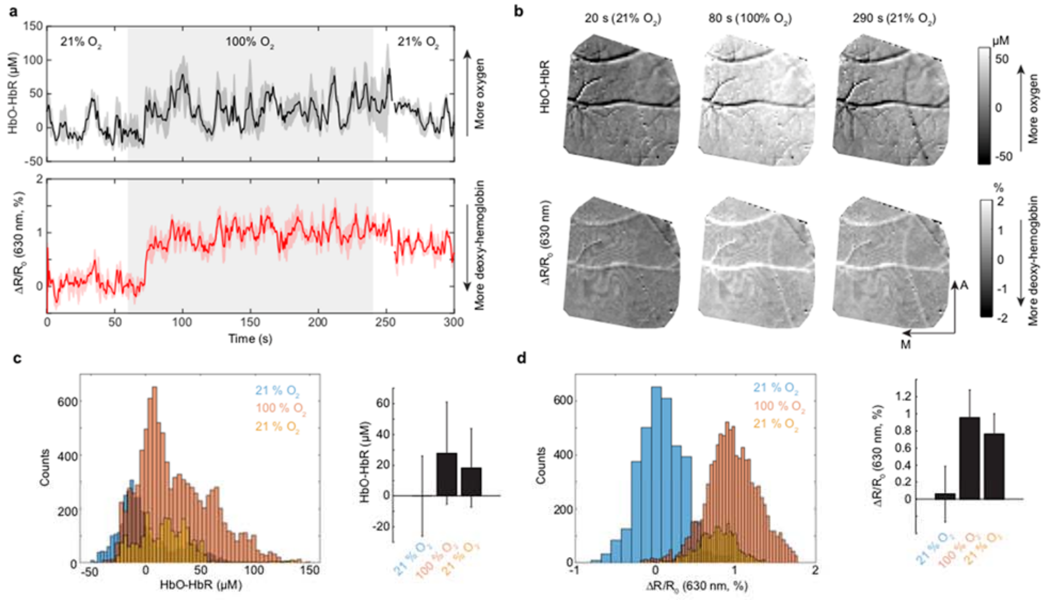

Figure B- Response to oxygen challenge. a) Average (4 trials) response to oxygen challenge (100% oxygen). Top, difference between oxy- and deoxy-hemoglobin concentration (HbO-HbR) response to oxygen challenge. Bottom, reflectance of 630 nm light (which is absorbed by HbR) change in response to oxygen challenge. b) Spatial distribution of HbO-HbR (top) and 630 nm light intensity change (bottom) before (20 s), during (80 s) and after (290 s) oxygen challenge. c) Left, histogram of HbO-HbR before (21% O2, blue), during (100% O2) and after (21% O2, orange) oxygen challenge. Right, average HbO-HbR before (21% O2, blue), during (100% O2) and after (21% O2, orange) oxygen challenge. Note the HbO-HbR increased ~30 uM during oxygen challenge. d) As (c) but for 630 nm light intensity change. Note that during oxygen challenge there is ~1% increase in intensity, indicates decreased deoxy-hemoglobin concentration (i.e., increased oxygenation).

2. The paper neglects completely the implications for human fMRI and it also neglects all the work that has been done in humans using hyperoxic challenges (see above as an example). The introduction and discussion needs to be completely redone, with a clear focus on why these results are important for hemodynamically-based neuroimaging studies. I do not doubt the relevance, but the authors have simply not done a good job in linking the results with the wider field of functional neuroimaging. As it stands, it is hard to see for an outsider why this is important and why the experiments were done at all.

As our experiments were done in mice, and as we did not use BOLD fMRI, we tried to be cautious in extrapolating our work to human fMRI work. Adding sections on the relevance to human fMRI to the introduction and discussion can easily be incorporated into a resubmitted manuscript.

3. The paper concentrates on CBV and CBF should be considered equally too.

4. Depth variations of pO2 are obvious, but the authors do not correlate this to depth resolved hemodynamic measurements.

Both of these aspects can be treated in a resubmitted manuscript.

5. CBF aspects are not considered in the modeling. This is problematic because hemodynamic regulation through constrictions/dilations come with changes in CBF and a pure O2 diffusion model comes short.

We have made models that incorporate CBF flow changes and these can be included in a resubmission.

6. Does the tissue cylinder stay constant in case of vessel diameter changes? This might be problematic,

because a diameter increase/decrease will lead to a tissue cylinder decrease/increase and hence a decreased/increased O2 need. Please check.

The tissue cylinder dilates with the vessel, so there is change in O2 demand due to changes in size of the tissue cylinder. Note that the arterial dilation is small (<2 micrometer change in diameter, Table 1).

7. Please discuss the fact that the different readouts potentially probe different cortical depths. IOS has a surface bias, etc.

Though we measured oxygenation at all levels in the cortex (and the dynamics at all layers are similar), the reviewer is correct that the IOS signal detects vasodilation primarily at the surface. Because the arterial and capillary networks are electrically connected (Hillman, Annual Review of Neuroscience, 2014) vasodilation at the surface reflects vasodilation deeper below. Several papers have shown that vasodilation initiates deep in the parenchyma (Tian, et al, PNAS, 2010; Rungta et al., Neuron, 2018) and propagates up to the surface in a few hundred milliseconds. The dilation at the surface always reflects dilations originating deeper down. We can add a discussion of this to revision.

8. Figure 1d. The authors mention a CBF decrease in FC, but I see an increase in FL/HL and much less so also in FC. Please clarify.

Because natural locomotion is of varying durations, we quantified CBF and CBV in two different ways. The first way was using the locomotion-triggered average, in which the responses to locomotion events between 5 and 10 seconds in duration separated from any previous locomotion event by 7 seconds are averaged together. This selects a subset of the total locomotion events, and is shown in Figure 1. The average locomotion-evoked CBF increase was not significantly greater than 0 ($p<0.22$). The second way is to calculate the hemodynamic response function (HRF), the linear filter that relates locomotion to CBF and CBV to locomotion (just as we do for oxygenation in Figure 2). The HRF method allows us to quantify the CBF and CBV responses to all locomotion events (short and long) together, and this is shown in Supplementary Figure 1. In principle, the HRF method is superior, but conceptually more complicated than the locomotion-triggered average. The HRF method shows a significant decrease in CBF and CBV in frontal cortex (Supplementary Figure 1). We apologize that our description of the differences between these methods was not clear, and this could be rectified in a resubmission.

We would like to thank the reviewers for their detailed criticisms, and the editors for the opportunity to submit a revised version of our manuscript.

In response to the reviewers' comments, we have added hyperoxia challenge experiments, additional measurements of respiration-related oxygen fluctuations in arteries, simulations of tissue oxygen dynamics that incorporate blood flow changes, and added more discussion of the relevance and implications of our results for fMRI.

Reviewer 1 and 3 were impressed with the novelty and quality of our study calling it "surprising", "interesting", "important", and the experiments are described as "thorough, multifaceted and elegant". Reviewer 1 has an excellent suggestion for a mechanistic experiment, but unfortunately this experiment cannot be cleanly interpreted because alterations of activity in the pre-Botzinger area drive changes in activity in the locus coeruleus (Yackle et al., 2017, Science), which cause downstream changes in cortical activity, blood flow and metabolism. Reviewer 2 was concerned that systemic changes, other than an increase in arterial oxygenation, could underly the increase in oxygenation in frontal cortex, and we have expanded the discussion and added simulations on why this is unlikely to be the case. Reviewer 3 thought there should be more focus on the relevance of our results to fMRI and a hyperoxia experiment added, both of which we have addressed.

Below we address the Reviewers' criticisms in detail.

Reviewers' comments:

Reviewer #1 (Remarks to the Author):

The manuscript "Cerebral oxygenation during locomotion is modulated by respiration" by Zhang et al. is outstanding on several levels. The findings are highly significant with broad reaching implications to the study of cerebrovasculature, neurovascular coupling, brain metabolism and functional brain imaging techniques. The experimental approaches are thorough, multifaceted and elegant. The data presentation is beautiful, and the analysis of complex data sets advanced, powerful and convincing. I was surprised by the main finding that respiration rate is a key driver of cerebral oxygenation and that several fundamental concepts regarding gamma band, vasodilation and systemic cardiac output/blood pressure can operate independent from each other and that they are not the most important predictive variables in determining brain PO2 during locomotion. I have one major comment below for the authors to consider as it would improve the manuscript and a few minor points.

Major
Though the conclusions drawn from the data and analysis are logical and convincing, one short coming is that there are no experiments of necessity and sufficiency that respiration is a primary driver of brain parenchyma PO2. This paper elegantly rules out the other likely possibilities and provides a very strong case that it must be respiration, but the causal experiments are missing. Is it feasible to directly increase or decrease the respiration rate and measure the predicted changes in PO2? What about injecting an AAV Gi-DREADD into the brain stem in either the Pre-Botz or RTN and then during the physiology experiment give systemic CNO or Compound 21 to decrease the respiration rate? Perhaps the authors have reasons not to try such an experiment? Lack of expertise?

We agree with the reviewer that manipulations of the activity of the respiratory-driving pre-Botzinger complex (pre-BotC) would be a "killer" experiment. Though technically difficult, such an experiment is in principle possible. However, there are issues that would make the results of such an experiment difficult, if not impossible, to interpret. Recent work (Yackle et al., 2017, Science; Yang and Feldman, J. Comp Neuro 2018) has elegantly shown that the respiratory central pattern generator drives a subset of pre-BotC neurons that directly excite the locus coeruleus (see **Figure A** below, adapted from Yackle et al 2017, Figure 4H), which then drives brain-wide changes in neural activity and other changes in metabolism. Stimulation of the locus coeruleus (LC) releases norepinephrine, which has a multitude of effects, including activation of astrocytes (Paukert et al. 2014, Neuron), changes in neural activity and blood flow (Toussay et al., 2014 J. Neurosci.), permeability of the blood brain barrier (Raichle et al., 1975, PNAS), and causes large changes in metabolism and glucose uptake (Craik et al, 1987, Brain research bulletin; Abraham et al., Brain research 1979). Thus, manipulating the activity of pre-BotC

neurons (or the regions that project to it, such as RTN) would have the side effect of driving brain-wide changes in neural activity and metabolism in addition to the desired effects on breathing. It would be very hard to interpret the results of this experiment.

Minor:

The authors have a data set at the end of the paper where they state that 4 out of 7 arteries showed a relationship between PaO2 and the respiratory cycle (fig 4j). It sounds like the other 3 experiments did not show this relationship, but it was not clear from the writing. Is there a valid reason not to include these 3 here? Was it because these 3 did not have a regular breathing cycle and could not be analyzed? If not, and these 3 were included, and the full data set was examined, does the overall relationship/significance disappear? I am not comfortable with arbitrarily binarizing the data unless there is an objective reason to do so. If this dataset was removed from the paper, it would not limit my enthusiasm.

Figure A, (adapted from Yackle, Science 2017). The respiratory rhythm generator projects to Cdh9/Dbx+ neurons, that then project to the locus coeruleus (LC). Any perturbation of activity in the pre-BotC will cause brain-wide changes in neural activity, metabolism, and blood flow. Stimulation VRG premotor neurons will conflict with the rhythmic drive coming out of the pre-BotC, so is not a candidate for stimulation.

We thank Reviewer 1 for pointing that the relationship between PaO2 and the respiratory cycle was poorly described, in both the Results and Methods sections. Briefly, our analysis method will not work if the respiration is not very regular. Therefore, we set a selection criterion using phase randomization test (see Methods). In response to the reviewer's concern, we have added two more experiments (bringing the total to nine arteries, of which six arteries show a significant phase relationship with respiration, shown in Fig.4h-j). We better explain with details about our selection criteria in the Methods section (Line 1108-1118), and re-emphasize this in the Results section (Line 328-335).

Line 1108-1118: *Arterial oxygen tension changes during the respiration cycle.* To evaluate the arterial oxygen tension change within the respiration cycle, we selected oxygen measurements during periods with regular respiratory rate (average frequency 2.5 Hz, SD ≤ 0.6 Hz, selected based on the criterion that the maximum respiration frequency was 5 Hz). The phosphorescent decays were aligned according to their place in the phase of the respiratory cycle (**Fig.4**g) and the average PaO_2 over the respiratory cycle was calculated. To further determine whether the fluctuations of oxygen tension was induced by respiration, we performed a phase randomization test: we calculated the power spectrum of arterial oxygen tension, and determined the peak frequency in the power spectrum, and checked that it was within the respiration frequency range. Statistical significance of this peak was calculated by reshuffling the arterial oxygen measurements[128], and the 95% confidence interval was calculated using 10000 reshuffled trials.

Line 328-335: In animals (n = 5) with long bouts of highly regular respiration rate (average frequency 2.5 Hz, SD ≤ 0.6 Hz, average frequency/SD > 4)，we tested whether fluctuations of PaO_2 tracked the respiratory cycle with a phase randomization test (see Methods). Six arteries (3 in the cortex, and 3 in the olfactory bulb) out of 9 had significant PaO_2 fluctuations synchronized with the respiratory cycle (**Fig.4**h-j). Note that even though 3 arteries did not pass the phase randomization test and were excluded, the plot in **Fig.4**j remained significant when including all 9 arteries (Wilcoxon signed rank test, p = 0.0039).

I would prefer the term "penetrating artery" be "penetrating arteriole". Maybe this is a simple matter that the authors use the term 'artery' in a general sense, but given the size of the penetrating vessels in the neocortex they definitely qualify as arterioles.

We have changed "penetrating artery" to "penetrating arteriole".

The authors surmise that autoregulation at the circle of Willis would control brain blood flow increases in the FC

when cardiac output increases. Why just the circle of Willis? Wouldn't all resistance vasculature undergo a myogenic response when pressure increases? Perhaps this argument needs to be refined.

The reviewer is correct that the resistance arterioles could play a role (Willie et al., 2014, J Physiology), recent evidence has suggested in humans that only vessels in the Circle of Willis constrict during exercise (Warnert et al.,2016 JCBFM). We have added the following sentence to the Results section.

Line 121-123: This lack of non-specific flow increase in the cortex during locomotion is likely because of autoregulation of the feeding arteries at the level of the circle of Willis and larger resistance arteries, as well as increased blood flow to the muscles[44].

A significant portion of the discussion is redundant with the results text. I like having a little 'discussion' in the results to convince the reader as they are going through the data, but I think it is best not to repeat the same points in the discussion section. Can the authors instead add to the discussion by speculating on the significance of their finding towards functions functional imaging, like IOS or fMRI?

In response to concerns from Reviewers 1 and 3, we have added more discussion of the relevance of our results to fMRI in the introduction (Line 61-71) and discussion (Line 446-466):

Line 61-71: In addition to the importance of understanding oxygen dynamics in the brain to basic physiology, a better grasp of natural oxygen dynamics in the brain will greatly aid in the interpretation of functional MRI signals[27], which allow non-invasive imaging of neural activity. Previous work in awake primates has shown that tissue oxygen signals correspond well with the changes observed with blood-oxygen level dependent (BOLD) fMRI[5,6]. Neurally-driven BOLD signals are generated by vessel dilation[28]. However, in addition to the BOLD fMRI signals of a neural origin (i.e., those generated by neurovascular coupling), BOLD signals can arise from sources that are not directly linked to underlying neural activity, such as pure vascular effects, respiration[29-32], cardiac pulse rate[33-35], and autonomous hemodynamic regulation[36]. Resolving the nature of these non-neural sources of BOLD contrast has been an area of active research[30,31,37], and will be helpful in better spatially resolving the neurally-generated BOLD signals[38].

Line 446-466: While our studies were performed in mice, there are respiration-driven fluctuations in the arterial blood of ungulates[76,77], suggesting it is a general property of mammals. Our finding may have useful implications for human fMRI work. Though the effects of respiration rate on CO_2 levels (which will cause changes in arterial diameter and blood flow on the scale of tens of seconds to minutes) have been appreciated in human neuroimaging[29,32,94,95], changes in systemic oxygenation due to respiration changes will be more rapid (of the order of a few seconds). While it is generally presumed that arterial blood is saturated in humans[59,70,96] (but see[21]), arterial oxygen tension decreases substantially with age[97] and acutely during sleep[98], and oxygen challenge in humans and monkeys[99,100] raises blood oxygenation just as in our mice (**Supplementary Fig.10**). This suggests that respiration may play a more important role in cerebral oxygenation in humans than is currently appreciated, particularly as respiration rate is actively modulated during cognitive tasks[101-103]. Respiration in humans is known to be increased following auditory or visual stimulation, and patterns of respiration differ from individual to individual, which might play a role in cerebral oxygen dynamics[104]. Recent work has shown that respiration is actively modulated during cognitive tasks in humans, and respiration dynamics predict task performance[105]. There is an emerging consensus that global brain activity is coordinated with respiration phase and rate in both animals and humans[106-108]. Because respiration will be modulated by tasks, there may be spurious, spatially distributed, non-neuronal BOLD signals locked to the stimulus driven by respiration changes. Fortunately, these artifacts can be removed by regressing out the effects of respiration, either using measures of systemic blood oxygenation, or measures of respiration itself[29,32,95,109-111].

Related comment: it would be fascinating to know whether this phenomenon applies to larger organisms, as there may be significant implications for functional optical and magnetic-based imaging in humans and non-human primates. I know the authors argued that it will apply, but there is no data yet, as expected. This could be fascinating for future work.

We are in complete agreement with the reviewer on this point and we hope that labs with expertise in human and non-human primate imaging will investigate this possibility.

Reviewer #2 (Remarks to the Author):

Zhang et al. investigate the influence of breathing on cerebral oxygenation during locomotion in awake head-fixed mice. Using imaging, spectroscopic, neurophysiological and polarographic approaches to investigate indices of cerebral perfusion, brain pO2, and neural activity, they found that locomotion increases oxygenation in neocortical regions irrespective of their involvement in locomotion, and in the olfactory bulb. The increase was independent of neural activity and of the associated cerebrovascular changes, but was correlated with the breathing pattern and arterial pO2. It is concluded that breathing is able to independently influence cerebral oxygenation.

This paper raises of number of concerns related to the lack of direct measurement of relevant physiological parameters that are critical for the interpretation of the findings. The lack of these critical measurements render data interpretation excessively speculative and uncertain.

1. Arterial blood pressure (AP) was not measured. As mentioned in the paper, AP changes with locomotion and its dynamic impact on cerebrovascular parameters has profound implication for oxygen delivery.

The question here is not whether blood pressure changes during locomotion (it does), but whether a change in blood pressure could account for the increase in oxygenation that we see in the frontal cortex, where there is no increase in blood flow. Through a series of experiments, we showed that the change in oxygenation cannot be accounted for by blood pressure changes.

The blood flow of the brain will depend on the resistance of the brain vasculature, the blood pressure, and the resistance of the rest of the body's vasculature. If blood pressure rises without any changes in vessel diameter, the flow of the blood to the brain (as measured with laser Doppler) will rise. Thus, there is the possibility that increases in arterial blood pressure could raise oxygenation in the tissue, but the pressure-induced increase in oxygenation is mediated by an increase in local blood flow.

The ways to test if blood flow in the brain is affected by changes in arterial blood pressure are to: 1) measure blood flow directly and 2) manipulate blood pressure to disrupt the blood flow-mediated changes in oxygenation. *We report the results of both of these experiments in the manuscript.* Using laser Doppler flowmetry, a measure of blood flow (Shih et al., JCBFM 2009), we found that there was no increase in blood flow to the frontal cortex. This categorically rules out the possibility that blood flow increases mediated by blood pressure drive the increase in oxygenation we see. The lack of flow increase is likely because blood vessels at the level of the circle of Willis constrict during exercise (Warnert et al, 2016, JCBFM), buffering the effects of increased blood pressure in the brain, though there are likely other mechanism that contribute.

Secondly, we pharmacologically increased and decreased blood pressure with atenolol and glycopyrrolate respectively (which do not cross the blood brain barrier) and saw no change in oxygenation (**Supplementary Figure 5**), replicating previous results from our lab which showed that modulations of systemic blood pressure do not affect blood flow in the frontal cortex (Huo, Green and Drew, Neuroimage 2015, Figure 3). If blood pressure played a role in driving the oxygenation changes, then atenolol (which reduces blood pressure) and/or glycopyrrolate (which elevates resting heart rate and blood pressure) should respectively block and occlude the oxygen changes in the cortex. However, we saw no differences in the blood oxygenation changes signals after injection of glycopyrrolate/atenolol and vehicle. Again, this result is inconsistent with the hypothesis that the blood pressure fluctuations have any impact on brain oxygenation.

Lastly, we observed increases in both tissue and arterial oxygenation when the respiratory rate varies in a stationary animal (Fig 4b and 4f), and that the blood oxygenation fluctuations tracked the respiratory cycle (Fig 4h-j). These results cannot be explained by blood pressure variations.

These issues are explained in the discussion section (Line 421-429):

Line 421-429: First, for the increases in cardiac output to raise global oxygenation in the cortex (independent of any changes in systemic oxygenation), it would need to drive an increase in cerebral blood flow. Our laser Doppler experiments show that blood flow does not rise in the frontal cortex, as they are likely buffered by resistance arterioles and autonomic regulation of the circle of Willis[90] (but see[91]). Additionally, when heart rate and blood pressure increases during locomotion were blocked (with the beta blocker atenolol, which does not cross the blood brain barrier) or occluded (with the muscarinic receptor antagonist glycopyrrolate which also does not cross the blood brain barrier), there was no change in the locomotion-evoked CBV change (**Supplementary Fig.5**, see also[64]).

2. Although "vasodilation" is mentioned extensively in the paper, vascular diameter and RBC flux were not measured. Therefore, it is unclear whether the indirect spectrophotometric indices used to assess hemodynamic factors accurately reflect vascular variables.

We measured cerebral blood flow with laser Doppler flowmetry (LDF, Fig 1C and D, Supplementary Figure 1). This is a standard technique that combines the velocity and backscatter signals to measure RBC flux in a ~1mm^3 volume with very high (up to ~5Hz) temporal resolution (Shih et al., JCBFM 2012). Doppler flowmetry has been extensively validated against quantitative measures of blood flow (such as Xe^{133}, see Tonnesen, Exp Physiology 2005 and references therein) and the flow increases measured with LDF match those made in single vessels with confocal microscopy (Barfod, Acta Physiol Scand 1997).

Hemoglobin is the strongest absorber of visible light in the brain, and imaging with light at an isobestic point of hemoglobin (which is absorbed equally well by deoxy- and oxy-hemoglobin) will report vessel dilation. Intrinsic signal imaging using one or more wavelengths of light is widely used to assay vessel dilation (see Sirotin and Das, 2009, Nature; Brennan et al., 2007, Journal of Neurophysiology). As dilating a vessel will increase the local concentration of hemoglobin, and hemoglobin concentration is far and away the largest determinant of light reflectance from the brain.

Intrinsic imaging has been used by multiple labs to measure vessel dilation (with validation with 2-photon microscopy) in work by the Kleinfeld lab (Mateo et al., 2017, Neuron), Elizabeth Hillman's lab (Ma et al., 2016, PNAS) and by ourselves (Gao, Huo, Drew, Neuroimage 2015). We have shown that arterial and venous dilations during locomotion (as measured directly with 2-photon microscopy) cause corresponding decreases in reflectance in green light in the intrinsic optical signal (see Huo, Gao, Drew, Neuroimage 2015 for an extensive discussion of this). We have also made extensive measurements of vascular diameters with two photon microscopy during locomotion and during other conditions (Drew et al., 2011, PNAS, Gao and Drew, 2016 J Neuroscience; Winder et al. 2017 Nature Neuroscience), and these measurements very closely match intrinsic optical signal measurements. Thus, changes in reflectance have been validated by multiple labs to be robust measures of vascular dilation.

To better show that the changes in the intrinsic signal report vessel dilation, we have added panel to **Supplementary Figure 1** showing concurrent measurements of vessel diameters and reflectance. The arteries dilate (~15%) at the onset of locomotion within 1-2 seconds, while the venous dilations are smaller and slower. These dilations are very similar to 2-photon measurements of arteries and veins in response to sensory stimulation (Drew et al., 2011, PNAS) and locomotion (Huo, Gao, and Drew, NeuroImage; Gao and Drew, 2016, J Neuroscience). Importantly, the measured dilations track the simultaneously measured changes in reflectance, showing that decreases in the IOS signal at 530nm report increases in vascular diameter.

3. Oxygen utilization and extraction fraction were not measured. These variables are critical for the interpretation of changes in interstitial pO2 and for the adjustments in oxygen delivery occurring during locomotion. Changes in O2 utilization could occur in the absence of changes in interstitial pO2.

For changes in oxygen utilization to explain the *increase* in tissue oxygenation in both the frontal and FL/HL cortices we see during locomotion, the increase in neural activity (Figure 1 and Supplementary Figure 2) would have to be accompanied by a ***decrease*** in metabolic activity (CMRO2). We know of no reports of increases in neural activity that are associated with a decrease in metabolic rate, as increases in neural activity cause increases in oxygen use (Logothetis, Nature, 2008; Buxton, Neuroimage, 2012). To address this issue, we performed simulations of oxygen dynamics in the frontal cortex where we decreased CMRO2 to see if we could

recreate the increase in tissue oxygen seen in our experiments (Supplementary Figure 11). We found that CMRO2 would have to *decrease 15%* in order to explain the ~2 mmHg increase in tissue oxygenation. Given that increases in firing rates, which we observe in FC (Figure 1 and Supplementary Figure 2) robustly increase CMRO2 (Sanganahalli et al., JCBFM 2016), it is hard to see how a decrease in CMRO2 could plausibly explain the observed increase in oxygenation in the frontal cortex accompanying locomotion.
Note that a decrease in CMRO2 would also not explain the correlation between oxygenation and respiration rate (Figure 4).

The oxygen extraction fraction (OEF) is the fraction of oxygen extracted from the blood in its transit through the brain, and is determined by the metabolic rate and blood flow (Buxton, Neuroimage, 2012; Buxton, Frontiers in Neuroenergetics, 2010). OEF is dimensionless parameter that relates the oxygenation of the veins (which is what the BOLD contrast mechanism is sensitive to) to the oxygenation of the arteries. OEF is not measured directly for a section of brain tissue, but rather can be calculated (with many assumptions) by measuring cerebral blood flow and oxygen use using, for example, positron emission tomography or fMRI (Buxton, Frontiers in Neuroenergetics, 2010). The OEF does not tell us anything quantitative about the quantities of interest, specifically the absolute values of arterial and tissue oxygenation, and it is not relevant to the experiments done here. This is why we made direct, quantitative measures of tissue and arterial oxygenation.

4. Changes in pC02 were not recorded, which can have profound effects on blood flow and oxygen delivery.

CO2 is a vasodilator (Cohen et al., JCBFM, 2002), and fluctuations in CO2 levels can affect arterial diameter and thus blood flow. Thus, CO2 levels could affect brain tissue oxygenation by dilating vessels. However, changes in pCO2 cannot explain the increases in oxygenation we see. First, CO2-mediated changes in vessel diameter are very slow, taking tens of seconds to occur, even when driven by inhalation of high levels of CO2 (Ngai and Winn, Amer. Journal Physiology, 1996), far too slow to account for the dilations that occur within a second of locomotion onset when breathing atmospheric levels of CO2. Secondly, CO2 levels in the blood *fall* with the onset of exercise in rodents (Fregosy and Dempsy, Journal of Applied Physiology, 1985). This exercise associated decrease of CO2 would drive vasoconstriction, decreased blood flow, and consequently cause a *decrease* in tissue oxygenation, the opposite of what we observed. Finally, for CO2 to drive the increases in oxygenation in the frontal cortex, it would need to dilate the vessels in the frontal cortex, which is the opposite of the constriction we observe there. Thus, changes in arterial CO2 *cannot* account for any of the oxygenation changes we see.

We have tried to better clarify these issues in the revised manuscript:

Line 431-436: <u>Second, while CO_2 is a strong vasodilator, and can drive increases in cerebral oxygenation under hypercapnia conditions by dilating blood vessels, rodents become hypocapnic during sustained exercise[92]. Exercise-evoked changes in CO_2 would tend to cause cerebral vasoconstriction and would tend to drive a deoxygenation. Again, this mechanism could not drive the observed increase in blood and tissue oxygenation in the frontal cortex without corresponding flow increases and vasodilation.</u>

5. Many of the observations were made in a limited number of mice (e.g., n=2) and considering the biological variability of the awake preparation more robust data is needed to draw conclusions in this experimental preparation.

We used three different techniques (polarography, spectroscopy, and two-photon phosphorescence lifetime measurements of oxygen sensors) in a total of 83 mice used in our experiments. The smallest animal n for an experiment in our paper is n=4 for some of our local intracranial infusion experiments. This is comparable to typical infusion experiment n's (Raposo et al., Nature Neuroscience 2014; Otchy et al., Nature, 2015). With a paired t-test with the standard alpha of 0.05 and a power (1-beta) of 0.8, an n=4 will allow us to detect effect size (mean difference/standard deviation) of 1.6 (using G*Power 3.1, Faul et al., Behav Res Methods, 2007). Given that the effect sizes we and others observe with these sorts of physiological experiments (in the range of ~3), an n=4 is enough.

The so-called "variability" frequently ascribed to the awake preparation is due to un-monitored behavior (locomotion, arousal fluctuations, grooming, whisking), as we and others have recently shown (see Winder et al., 2017 Nature Neuroscience; Drew, Winder, Zhang, 2019 Neuroscientist; Chang … Duyn, 2016 PNAS; Musall

et al., BioRxiv doi: 10.1101/308288;Salkoff et al., BioRxiv doi: 10.1101/709642; Stringer et al., 2019, Science). These spontaneous movements drive both neural activity and vasodilation. For example, the variability in arterial dilation latency evoked by locomotion in the awake animal are less than those evoked by somatosensory stimulation in the anesthetized animal (Gao, Greene, Drew, NeuroImage 2015). In our experiments, we carefully monitored behavior (locomotion and respiration), and aligned our responses to locomotion, so variability was minimal. This can be seen clearly in Figure 1b, where there is a ±1% variability in reflectance in the FL/HL area when the mouse is still, and a 10% change with sustained locomotion. If behavior was not monitored in this case, the signal would (erroneously) appear to be very unstable.

6. Some of the interpretations do not seem to agree with the data. For example, it is said that locomotion decreases CBF and CBV in the frontal cortex (page 5, line 98), but the related figure (fig 1d) seem to show no significant changes in these variables.

Because natural locomotion is of varying durations, we quantified both CBF and CBV in two different ways. The first way was using the locomotion-triggered average, in which the responses to locomotion events between 5 and 10 seconds in duration separated from any previous locomotion event by 7 seconds are averaged together. This selects a subset of the total locomotion events and is shown in Figure 1. The average locomotion-evoked CBF increase was not significantly greater than 0 ($p<0.22$). The second way is to calculate the hemodynamic response function (HRF), a linear filter that relates locomotion to CBF and CBV to locomotion (just as we do for oxygenation in Figure 2). The HRF method allows us to quantify the CBF and CBV responses to all locomotion events (short and long) together, and this is shown in Supplementary Figure 1. In principle, the HRF method is superior, but conceptually more complicated than the locomotion-triggered average. The HRF method shows a significant decrease in both CBF and CBV in frontal cortex (Supplementary Figure 1). We apologize that our description of the differences between these two approaches was not clear, and have clarified this in the revision:

Line 105-121: We quantified how locomotion affected both CBV and CBF in two complimentary ways. We calculated the locomotion-triggered average, generated by aligning the IOS or laser Doppler signals to the onset of locomotion (see Methods) using only locomotion events ≥ 5 seconds in duration (**Fig.1**d). The locomotion-triggered average showed no significant change in CBF in the FC ($n = 5$ mice, Wilcoxon signed-rank test, $p = 0.22$), and a large increase in FL/HL ($n = 5$ mice, Wilcoxon signed-rank test, $p = 0.03$). The locomotion-triggered average showed significant increase in optical intensity in FC ($n = 11$ mice, Wilcoxon signed-rank test, $p < 0.001$) while decrease in optical intensity in FL/HL ($n = 11$ mice, Wilcoxon signed-rank test, $p = 0.0122$). We also calculated the hemodynamic response function (HRF)[39,43], which is the linear kernel relating locomotion events to observed changes in CBV and CBF (**Supplementary Fig.1**), using all locomotion events. When we quantified the net CBF using the HRFs, locomotion actually drove a significant decrease in flow in FC (Wilcoxon signed-rank test, $p = 0.03$) and increase in FL/HL (Wilcoxon signed-rank test, $p = 0.03$, **Supplementary Fig.1**). Using the HRFs to quantify the net CBV, we obtained same conclusions as derived from locomotion-triggered average. This shows that locomotion and the accompanying cardiovascular changes do not drive global increases in CBF/CBV, rather CBF/CBV increases are under local control.

7. Irrespective of these reservations, the conclusions of the study are rather expected: there is a global increase in brain oxygenation which is linked to the known variation in pO2 occurring with breathing.

The assumption in fMRI and neurovascular coupling studies is that the arterial blood is completely saturated with oxygen (see for example: Kim and Ogawa, 2012 JCBFM; Buxton, 2012, Rep. Prog Phys; Hillman, 2014, Annual Review of Neuroscience) and can be treated as a “constant”. If the arterial blood is saturated, then increasing respiration should not cause an increase in arterial oxygenation, as it cannot take on any more oxygen. To our knowledge, there has been no previous study examining the interactions between respiration and brain oxygenation in a behaving animal. This is an important area of interest, as many animal and human studies have shown that brain-wide electrical signal and behavior are locked to the respiration cycle (Moore et al., Nature 2013; Moberly et al., 2018 Nature Communications; see also Tort et. al, 2018 Trends in Neuroscience for a review). We also know of no models where the blood oxygenation varies dynamically on the timescales seen here, oxygen saturation is invariably set to a constant and not changed.

8. "Breathing" may be more appropriate then "respiration".
In the literature, respiration and breathing are both commonly used. We have added “breathing” to the abstract.

Reviewer #3 (Remarks to the Author):

This is a very interesting manuscript that addresses the important link and dissociation between systemic oxygen delivery to the brain tissue caused by locomotion and respiration and hyperemia/neurovascular coupling induced changes in blood and tissue oxygenation. The authors present an impressive collection of results, which are based on multimodal experimental assays. The experiments are performed on the highest level, using top-notch technology in the awake behaving mouse. The manuscript follows a very stringent line of thought and the results are clear and well presented.

I have a few major concerns:

1. The work lacks an experimental approach in which brain oxygenation is increased without locomotion. This is needed to nail down the paper's main claims. The breathing cycle experiment is very elegant and goes in that direction, but it is not sufficient. A good additional experiment would be 100% oxygen breathing. This would give the paper also a good translational twist, as this is used in humans (e.g. c.f. Fan et al., 2016. Neuroimage 125, 920-93.).

We agree with the reviewer’s criticism, and have added additional experiments where the mice breath 100% oxygen (Supplementary Figure 10). Consistent with the human literature, we observe a substantial increase in blood oxygenation. This result shows that blood oxygenation can rise without locomotion.
The details of oxygen challenge experiments (Line 996-1006) and results (Line 340-354) can be found in the manuscript.

2. The paper neglects completely the implications for human fMRI and it also neglects all the work that has been done in humans using hyperoxic challenges (see above as an example). The introduction and discussion needs to be completely redone, with a clear focus on why these results are important for hemodynamically-based neuroimaging studies. I do not doubt the relevance, but the authors have simply not done a good job in linking the results with the wider field of functional neuroimaging. As it stands, it is hard to see for an outsider why this is important and why the experiments were done at all.

As our experiments were done in mice, and as we did not use BOLD fMRI, we tried to be cautious in extrapolating our work to human fMRI work. We have added sections to the introduction (Line 61-71) and discussion (Line 446-466) on the relevance of this work for human fMRI:

Line 61-71: In addition to the importance of understanding oxygen dynamics in the brain to basic physiology, a better grasp of natural oxygen dynamics in the brain will greatly aid in the interpretation of functional MRI signals[27], which allow non-invasive imaging of neural activity. Previous work in awake primates has shown that tissue oxygen signals correspond well with the changes observed with blood-oxygen level dependent (BOLD) fMRI[5,6]. Neurally-driven BOLD signals are generated by vessel dilation[28]. However, in addition to the BOLD fMRI signals of a neural origin (i.e., those generated by neurovascular coupling), BOLD signals can arise from sources that are not directly linked to underlying neural activity, such as pure vascular effects, respiration[29-32], cardiac pulse rate[33-35], and autonomous hemodynamic regulation[36]. Resolving the nature of these non-neural sources of BOLD contrast has been an area of active research[30,31,37], and will be helpful in better spatially resolving the neurally-generated BOLD signals[38].

Line 446-466: While our studies were performed in mice, there are respiration-driven fluctuations in the arterial blood of ungulates[76,77], suggesting it is a general property of mammals. Our finding may have useful implications for human fMRI work. Though the effects of respiration rate on CO_2 levels (which will cause changes in arterial diameter and blood flow on the scale of tens of seconds to minutes) have been appreciated in human neuroimaging[29,32,94,95], changes in systemic oxygenation due to respiration changes will be more rapid (of the order of a few seconds). While it is generally presumed that arterial blood is saturated in humans[59,70,96] (but see[21]), arterial oxygen tension decreases substantially with age[97] and acutely during sleep[98], and oxygen challenge in humans and monkeys[99,100] raises blood oxygenation just as in our mice (**Supplementary Fig.10**). This suggests that respiration may play a more important role in cerebral oxygenation in humans than is currently appreciated, particularly as respiration rate is actively modulated during cognitive tasks[101-103]. Respiration in

humans is known to be increased following auditory or visual stimulation, and patterns of respiration differ from individual to individual, which might play a role in cerebral oxygen dynamics[104]. Recent work has shown that respiration is actively modulated during cognitive tasks in humans, and respiration dynamics predict task performance[105]. There is an emerging consensus that global brain activity is coordinated with respiration phase and rate in both animals and humans[106-108]. Because respiration will be modulated by tasks, there may be spurious, spatially distributed, non-neuronal BOLD signals locked to the stimulus driven by respiration changes. Fortunately, these artifacts can be removed by regressing out the effects of respiration, either using measures of systemic blood oxygenation, or measures of respiration itself[29,32,95,109-111].

3. The paper concentrates on CBV and CBF should be considered equally too.

We have added simulations taking into account dynamic changes in CBF (see Line 1146-1216 for details of the model), added better explanation of the Doppler measurements of CBF, and have tried to emphasize throughout the manuscript that the dilations (increases in CBV) we observe will drive increases in CBF. We have emphasized this in the results section where the CBV changes are discussed:

Line 95-97: Finally, local changes in cerebral blood flow (CBF) are intimately linked with vessel diameter[40], as dilations of vessels will reduce the resistance of the vascular network, increasing the blood flow through it.

4. Depth variations of pO2 are obvious, but the authors do not correlate this to depth resolved hemodynamic measurements.

We have added more analysis of baseline oxygen levels and two more panels to Supplementary Figure 4 where we quantify the onset time and time to peaks for the oxygen HRFs obtained in different cortical layers. We find that oxygen levels are comparable across the FC and FL/HL, and the HRF onset (but not peak) was faster in the FC than in the FL/HL. The laminar tissue oxygenation signal should qualitatively match the laminar BOLD signal. This is summarized in the results:

Line 166-179: We observed a laminar-dependence of resting PtO_2 in awake mice, with lower oxygenation in layer I than other layers in both FL/HL and FC (**Supplementary Fig.4**a, b, see also[4,24]), though this is probably too thin a section of brain to be distinguishable with laminar fMRI. Resting PtO_2 levels were similar at each cortical depth in both FL/HL and FC (**Supplementary Fig.4**a, b). No difference in onset time or peak time was observed in the HRFs of PtO_2 across layers, though the onset time was shorter in the FC (**Supplementary Fig.4**c, d), consistent with fMRI measurements using ultrashort stimuli[61]. Note that because we are measuring the tissue oxygenation in this case, any oxygen dynamics in the vasculature will be temporally blurred by the diffusion dynamics of oxygen in the tissue. These results, together with the observation that resting PtO_2 is similar in somatosensory cortex and the olfactory bulb glomerular layer[4], indicate that the spatial distribution of oxygen in the brain under normal (non-anesthetized) physiological condition is relatively homogenous. This quantification of laminar tissue oxygen dynamics should aid interpreting the complicated dynamics of laminar fMRI signals[62].

5. CBF aspects are not considered in the modeling. This is problematic because hemodynamic regulation through constrictions/dilations come with changes in CBF and a pure O2 diffusion model comes short.

We agree with the reviewer's concern. In response, we have made a new model (see Line 1146-1216 for details of the model) that includes dilation-evoked CBF changes (shown in a new Figure 5). When we include changes in CBF, the model results closely match the observed changes in tissue oxygenation.

6. Does the tissue cylinder stay constant in case of vessel diameter changes? This might be problematic, because a diameter increase/decrease will lead to a tissue cylinder decrease/increase and hence a decreased/increased O2 need. Please check.

The tissue cylinder dilates with the vessel, so there is no change in O2 demand due to changes in size of the tissue cylinder. Note that the arterial dilation is small (<2 micrometer change in diameter, Table 1).

7. Please discuss the fact that the different readouts potentially probe different cortical depths. IOS has a surface bias, etc.

Though we measured oxygenation at all levels in the cortex (and the dynamics at all layers are similar), the reviewer is correct that the IOS signal detects vasodilation primarily at the surface. Because the arterial and capillary networks are electrically connected (Hillman, Annual Review of Neuroscience, 2014) vasodilation at the surface reflects vasodilation deeper below. Vasodilation initiates in the parenchyma (Rungta et al., Neuron, 2018) and propagates up to the surface in a few hundred milliseconds. The dilation at the surface always reflects dilations originating deeper down.

To address this, we have added the following to the discussion:

Line 409-416: Note that our measures have different depth specificity. Visible light will primarily assay the upper few hundred micrometers of cortex, while laser Doppler uses infrared light which should sample CBF flow through the upper millimeter or so of cortex. The entire depth of cortex was sampled with oxygen-sensitive and neural activity electrodes. However, we found that tissue oxygen dynamics were relatively homogenous across layers. This is consistent with previous work showing the dilation signal initiates in the parenchyma causing a nearly instantaneous, electrically conducted dilation of the arteriolar tree[70,89], so that the dilations of surface vessels in general reflect the dilation dynamics within the brain.

8. Figure 1d. The authors mention a CBF decrease in FC, but I see an increase in FL/HL and much less so also in FC. Please clarify.

Because natural locomotion is of varying durations, we quantified CBF and CBV in two different ways. The first way was using the locomotion-triggered average, in which the responses to locomotion events between 5 and 10 seconds in duration separated from any previous locomotion event by 7 seconds are averaged together. This selects a subset of the total locomotion events, and is shown in Figure 1. The average locomotion-evoked CBF increase was not significantly greater than 0 ($p<0.22$). The second way is to calculate the hemodynamic response function (HRF), the linear filter that relates locomotion to CBF and CBV to locomotion (just as we do for oxygenation in Figure 2). The HRF method allows us to quantify the CBF and CBV responses to all locomotion events (short and long) together, and this is shown in Supplementary Figure 1. In principle, the HRF method is superior, but conceptually more complicated than the locomotion-triggered average. The HRF method shows a significant decrease in CBF and CBV in frontal cortex (Supplementary Figure 1). We apologize that our description of the differences between these methods was not clear and we have clarified these results in the revised manuscript:

Line 105-121: We quantified how locomotion affected CBV and CBF in two complimentary ways. We calculated the locomotion-triggered average, generated by aligning the IOS or laser Doppler signals to the onset of locomotion (see Methods) using only locomotion events ≥ 5 seconds in duration (**Fig.1**d). The locomotion-triggered average showed no significant change in CBF in the FC (n = 5 mice, Wilcoxon signed-rank test, $p = 0.22$), and a large increase in FL/HL (n = 5 mice, Wilcoxon signed-rank test, $p = 0.03$). The locomotion-triggered average showed significant increase in optical intensity in FC (n = 11 mice, Wilcoxon signed-rank test, $p < 0.001$) while decrease in optical intensity in FL/HL (n = 11 mice, Wilcoxon signed-rank test, $p = 0.0122$). We also calculated the hemodynamic response function (HRF)[39,43], which is the linear kernel relating locomotion events to observed changes in CBV and CBF (**Supplementary Fig.1**), using all locomotion events. When we quantified the net CBF using the HRFs, locomotion actually drove a significant decrease in flow in FC (Wilcoxon signed-rank test, $p = 0.03$) and increase in FL/HL (Wilcoxon signed-rank test, $p = 0.03$, **Supplementary Fig.1**). Using the HRFs to quantify the net CBV, we obtained same conclusions as derived from locomotion-triggered average. This shows that locomotion and the accompanying cardiovascular changes do not drive global increases in CBF/CBV, rather CBF/CBV increases are under local control.

REVIEWERS' COMMENTS:

Reviewer #1 (Remarks to the Author):

I have no further concerns. The authors have adequately addressed my queries with more information, more experiments and new discussion elements. This is an interesting and novel manuscript and in my opinion worthy of Nat Comm.

Reviewer #2 (Remarks to the Author):

The revised paper addressed the comments of the referees with new data and analyses, as well as changes in the text. The data show that ventilation-associated increases in cerebral arteriolar blood oxygenation modulate the oxygenation of the brain tissue globally.

The revised paper addresses all the technical concerns raised in the previous review, but the question of the novelty and impact of the observations remains unanswered.

The response in the rebuttal letter states that "The assumption in fMRI and neurovascular coupling studies is that the arterial blood is completely saturated with oxygen…and can be treated as a "constant". If the arterial blood is saturated, then increasing respiration should not cause an increase in arterial oxygenation, as it cannot take on any more oxygen."

However, this does not seem to be the case because in the revised paper it is demonstrated that ventilation with 100% O_2 increased cerebral arteriolar oxygenation and brain oxygenation. Thus, the paper states that "These results above indicate that the blood was not saturated with oxygen at rest." Therefore, it would appear that increases in ventilation lead to changes in arterial oxygenation which then result increased in brain oxygenation.

The critical question that remains unanswered is whether such increase in oxygenation during locomotion occurs only in neocortical arterioles, in which PaO_2 was measured with the phosphorescent probe, or also in extracerebral vessels feeding the brain. If there is a systemic increase in blood oxygenation during locomotion, as reflected by arterial blood gasses measurement in the periphery, then the findings are expected, i.e., the increase in systemic blood oxygenation leads to a global increase in brain oxygenation. However, if there is no increase in systemic pO_2 but there is an increase in brain arteriolar pO_2, then the finding would be novel and exciting because they would suggest that brain vessels have the unique capacity to increase blood oxygenation. However, the mechanisms of this effect would need to be investigated to provide an explanation for the observation.

Reviewer #3 (Remarks to the Author):

The authors closely followed my suggestions and I believe the manuscript's quality has improved considerably during the revision process. From my point of view, the paper can be published.

REVIEWERS' COMMENTS:

Reviewer #1 (Remarks to the Author):

I have no further concerns. The authors have adequately addressed my queries with more information, more experiments and new discussion elements. This is an interesting and novel manuscript and in my opinion worthy of Nat Comm.

We thank the reviewer for their constructive comments that helped improve the manuscript.

Reviewer #2 (Remarks to the Author):

The revised paper addressed the comments of the referees with new data and analyses, as well as changes in the text. The data show that ventilation-associated increases in cerebral arteriolar blood oxygenation modulate the oxygenation of the brain tissue globally.

The revised paper addresses all the technical concerns raised in the previous review, but the question of the novelty and impact of the observations remains unanswered.

The response in the rebuttal letter states that "The assumption in fMRI and neurovascular coupling studies is that the arterial blood is completely saturated with oxygen…and can be treated as a "constant". If the arterial blood is saturated, then increasing respiration should not cause an increase in arterial oxygenation, as it cannot take on any more oxygen."

However, this does not seem to be the case because in the revised paper it is demonstrated that ventilation with 100% O_2 increased cerebral arteriolar oxygenation and brain oxygenation. Thus, the paper states that "These results above indicate that the blood was not saturated with oxygen at rest." Therefore, it would appear that increases in ventilation lead to changes in arterial oxygenation which then result increased in brain oxygenation.

The critical question that remains unanswered is whether such increase in oxygenation during locomotion occurs only in neocortical arterioles, in which PaO_2 was measured with the phosphorescent probe, or also in extracerebral vessels feeding the brain. If there is a systemic increase in blood oxygenation during locomotion, as reflected by arterial blood gasses measurement in the periphery, then the findings are expected, i.e., the increase in systemic blood oxygenation leads to a global increase in brain oxygenation. However, if there is no increase in systemic pO_2 but there is an increase in brain arteriolar pO_2, then the finding would be novel and exciting because they would suggest that brain vessels have the unique capacity to increase blood oxygenation. However, the mechanisms of this effect would need to be investigated to provide an explanation for the observation.

We thank the reviewer for raising this question of whether such an increase in oxygenation during locomotion occurs only in the cortex. Our work has shown that brain oxygenation dynamics follows the systemic oxygenation change, and the brain has no unique capacity to increase blood oxygenation. This is clearly shown by the oxygen levels in the arteries tracking respiration cycle. The detailed information addressing this can be found in the main text, as follows:
"As the blood in these arteries will have minimal time to exchange oxygen in their transit through the heart and carotid artery to the brain, the oxygen levels in these arteries will track systemic oxygenation levels."

“Taken together, these measurements are consistent with an increase in systemic blood oxygenation that leads to a brain-wide increase of oxygenation in the tissue and vascular compartments during locomotion.”

In response to the reviewer’s comments regarding the novelty and importance of this work, our findings have suggested an independent pathway to regulate brain oxygenation besides neurovascular coupling, which has been overlooked in functional brain imaging studies. To our knowledge, there has been no study showing that normal respiration rate dynamics can alter the oxygenation in the brain in awake animals independent of vasodilation. The reviewer has provided no citations to back up the assertion of a lack of novelty, and the other two reviewers perceived it as novel.
Our direct measurement of brain oxygenation and quantification of its relationship with respiration cycle have not been done before. Our findings clearly advanced this research, which provides evidence of brain oxygen dynamics regulated by the respiration on a cycle-to-cycle basis. Using awake behaving animals, we also provided a brain oxygenation reference under physiological conditions, which will provide a reference for future studies of cerebral vasculature, neurovascular coupling, brain metabolism and functional brain imaging techniques.

Reviewer #3 (Remarks to the Author):

The authors closely followed my suggestions and I believe the manuscript's quality has improved considerably during the revision process. From my point of view, the paper can be published.

We thank the reviewer for their constructive comments that helped improve the manuscript.
